# Structural geology data and 3-D subsurface models of the Budgell Harbour Stock and associated dykes, Newfoundland, Canada

**DOI:** 10.1016/j.dib.2018.10.072

**Published:** 2018-10-25

**Authors:** Alexander L. Peace, J. Kim Welford, Meixia Geng, Hamish Sandeman, Brant D. Gaetz, Sarah S. Ryan

**Affiliations:** a Memorial University of Newfoundland, Department of Earth Sciences, St. John’s, NL, Canada; b Institute of Geophysics and Geomatics, China University of Geosciences, China; c Department of Natural Resources – Geological Survey, Government of Newfoundland and Labrador, St. John’s, NL, Canada

## Abstract

The data presented in this article are primarily related to the Tectonophysics research article “Rift-related magmatism on magma-poor margins: Structural and potential field analyses of the Mesozoic Notre Dame Bay intrusions, Newfoundland, Canada and their link to North Atlantic Opening” Peace et al. (2018). The present data article contains structural geology data from lamprophyre dykes and surrounding country rock in proximity to the Mesozoic gabbroic Budgell Harbour Stock (BHS), Newfoundland, Canada, in addition to sub-surface density and susceptibility models of the main igneous body. The structural geology data include: dyke locations, orientations, thickness and marginal lineations, in addition to country rock bedding and kinematic data from nearby faults. The 3-D sub-surface density and susceptibility models were derived from the inversion of magnetic and full tensor gradiometry (FTG) data, respectively, using a probabilistic approach to inversion described in the Society of Exploration Geophysicists (SEG) 2018 Technical Program Expanded Abstract “3-D inversion of airborne gravity gradiometry data for the Budgell Harbour Stock: A case history of using a probabilistic approach” Geng et al. (2018).

**Specifications table**

**Table:** 

Subject area	Earth and planetary science.
More specific subject area	Geophysics and structural geology.
Type of data	Spreadsheets (.xlsx), GoogleEarth (.kmz) and field geology photographs (.jpg).
How data were acquired	Field studies and modelling of potential field data.
Data format	Raw
Experimental factors	• The field structural geology data were acquired during geological fieldwork in spring and summer 2017. • The subsurface magnetic susceptibility and density models are the results of inversion of magnetic and full tensor gradiometry (FTG) data, respectively, using a probabilistic inversion approach described in [2]. • The original FTG data were acquired by Bell Geospace, collected on behalf of Celtic Minerals Ltd. [3]. • The original aeromagnetic data were extracted from a compilation made by the Newfoundland and Labrador Government Department of Natural Resources [4].
Experimental features	• Raw field geology data and sub-surface 3-D geophysical model results. • Structural geology data include: select field photographs, dyke locations, orientation, marginal lineations and thicknesses, as well as proximal bedding orientation and fault slickenlines. • Potential field data models include: sub-surface 3-D models of density and magnetic susceptibility, as well as Euler solutions using the magnetic and FTG data.
Data source location	Notre Dame Bay, northern-central Newfoundland, Canada.
Data accessibility	The data are with the article.
Related research articles	[1] A.L. Peace, J.K. Welford, M. Geng, H. Sandeman, B.D. Gaetz, S.S. Ryan, Rift-related magmatism on magma-poor margins: Structural, petrological and potential field analyses of the Mesozoic Notre Dame Bay intrusions, onshore Newfoundland, Canada and their link to North Atlantic Opening, *Tectonophysics*. 745 (2018) 24–45. doi:10.1016/j.tecto.2018.07.025.
	[2] M. Geng, J.K. Welford, C.G. Farquharson, A.L. Peace, 3-D inversion of airborne gravity gradiometry data for the Budgell Harbour Stock: A case history of using a probabilistic approach, in: *Society of Exploration Geophysicists (SEG),* October 14th-19th 2018, Anaheim, Ca, USA, 2018. doi: 10.1190/segam2018-2978510.1

**Value of the data**

• The Budgell Harbour Stock (BHS), located in northern-central Newfoundland (Fig. 1) is of scientific interest both to those working on rift processes [5] due to its reported cotemporaneous age with rifting prior to North Atlantic opening [6], [7], [8], [9], [10], [11] and also as a mineral prospect in its own right [3]. Thus, these data could prove useful for any future research on the BHS.
• In particular, the sub-surface models may be of use for mineral exploration of the intrusion, the dyke locations could be used to guide future sampling for petrology or further structural mapping, and the fault kinematic data may be useful as a starting point for palaeostress analysis.
• In addition, these data would also be suitable for comparison with other similar igneous bodies from different locations and geodynamic settings, or the results of analogue and numerical modelling.

## 1 Data

The datasets associated with this article were analysed in detail in [1] to study the structure, emplacement mechanisms and nature of the BHS and associated lamprophyre dykes. Here, structural geology data from the lamprophyre dykes and surrounding country rock are provided (Fig. 2) in addition to sub-surface density and susceptibility models derived from the inversion of magnetic and full tensor gradiometry (FTG) data, respectively (Fig. 3).

**Fig. 1:**
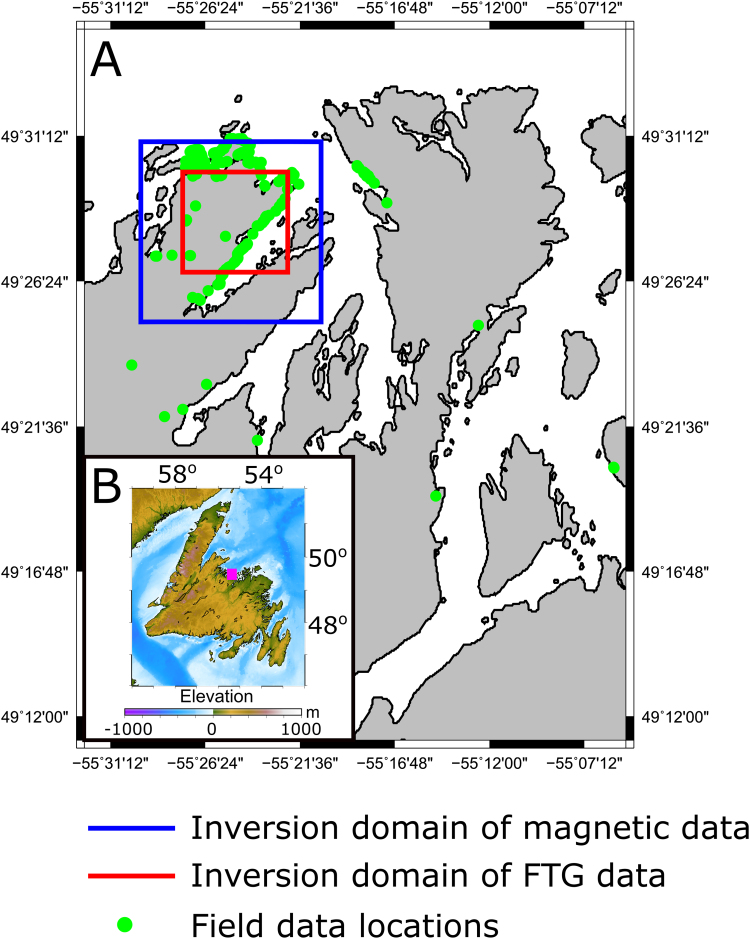
(A) Map of central Notre Dame Bay, Newfoundland, Canada showing: 1) the locations of field data, 2) modeled domain for the FTG data and 3) aeromagnetic data that resulted in the 3-D density and magnetic susceptibility models, respectively, provided herein. (B) Topographic map of Newfoundland showing the location of subfigure (A) using the topography of [14] V18.1.

**Fig. 2:**
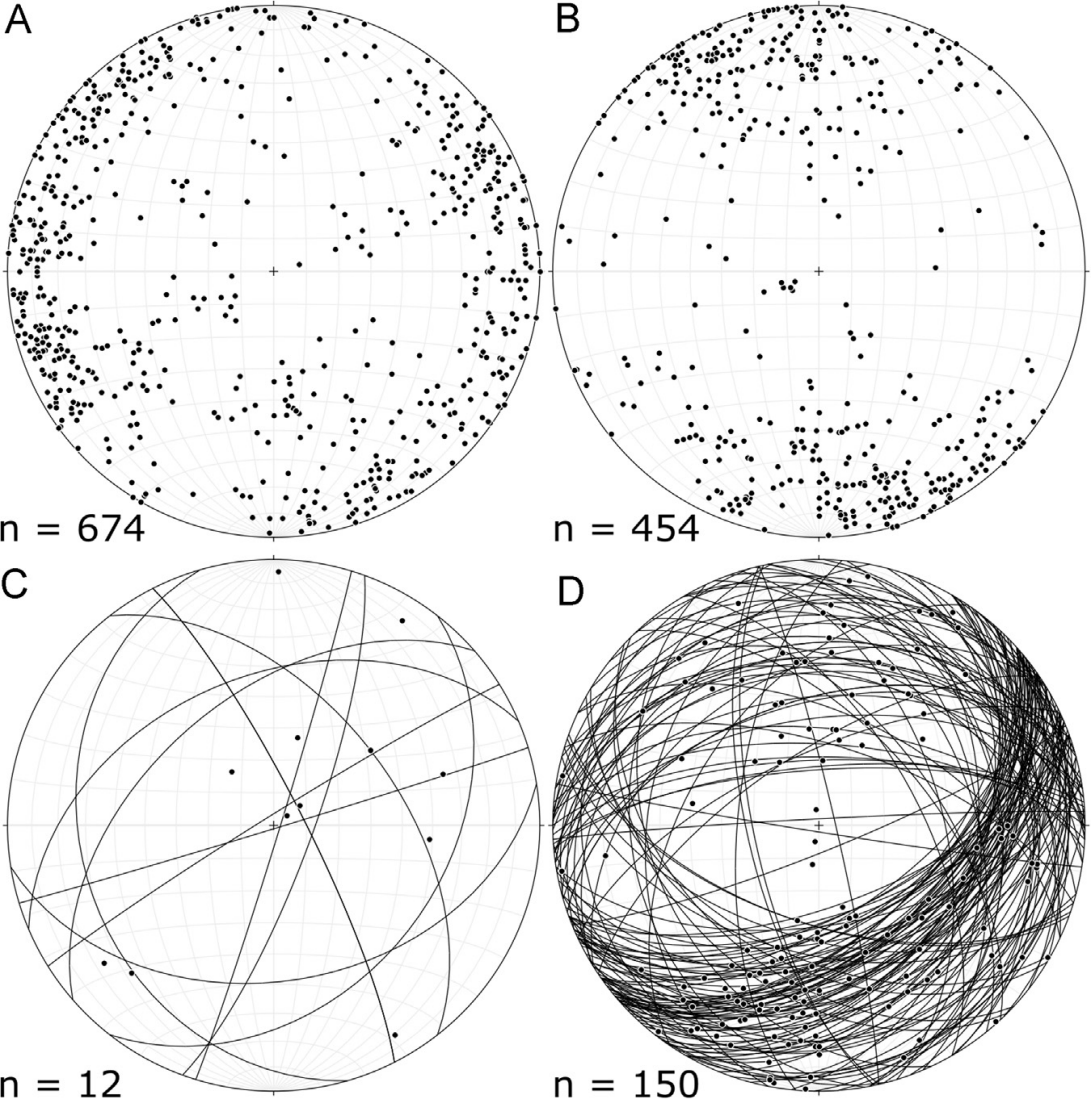
Stereographic projections of the structural data: (A) dyke margin measurements as poles, (B) bedding measurements as poles, (C) dyke margin lineations (poles) and the planes they lie on, and D) slickenlines (poles) and the planes they lie on.

**Fig. 3:**
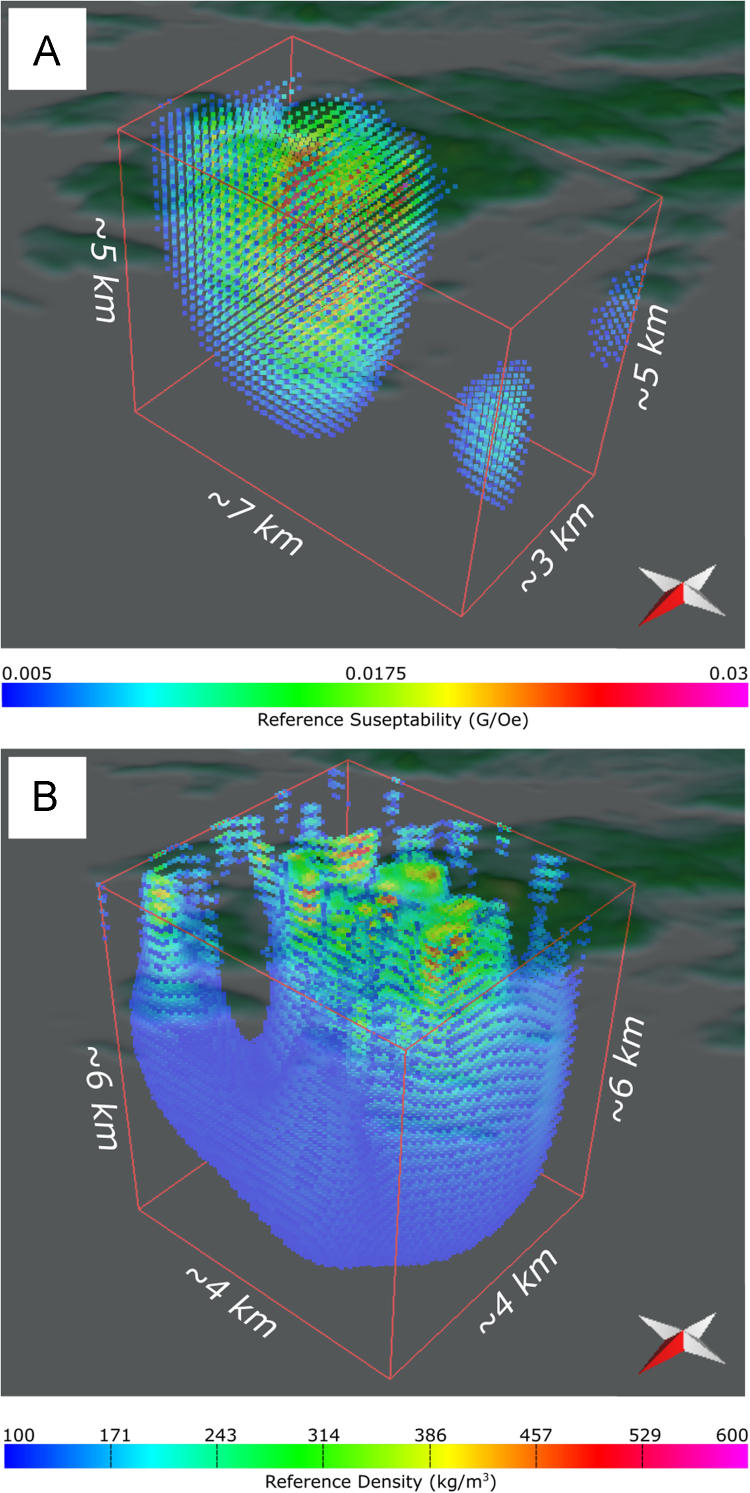
(A) The sub-surface susceptibility model with all reference susceptibilities < 0.005 G/Oe removed. (B) The sub-surface density model with all reference densities < 100 kg/m^3^ removed. Geospatial.

Specifically, the structural geology data obtained through the field-based study are provided as a spreadsheet (.xlsx). This spreadsheet contains: 1) dyke locations, orientation and thicknesses, 2) bedding orientations, 3) slickenline orientations (kinematic indicators), and 4) dyke margin lineations as separate sheets. In addition, a separate spreadsheet (.xlsx) is provided that contains details of the field photographs associated with the project, as well as a Google Earth file (.kmz) containing the locations provided in the main structural geology data spreadsheet.

The subsurface 3-D models from the inversion of potential field data are also included as a separate data spreadsheet (.xlsx). This spreadsheet contains four data sets: 1) 3-D density model, 2) 3-D magnetic susceptibility model, 3) the Euler depths obtained by modelling the FTG data, and 4) the Euler depths obtained by modelling the magnetic data.

## 2 Experimental design, materials and methods

### 2.1 Structural geology data acquisition

Geological field-work was conducted across Notre Dame Bay, Newfoundland during spring and summer 2017. To collect the data, this work involved mapping the locations, field relationships, orientations, and thicknesses of lamprophyre dykes associated with the BHS. A total of 292 dykes were recorded. From these, 655 measurements were made that include complete orientation and location data. In addition, 19 entries have incomplete orientation data (i.e. strike, dip or dip-direction not obtained) and a further 9 were recorded as location only. The vast majority of the measurements were obtained by direct measurement. However, some data were obtained indirectly from a small boat where landing the boat was considered impractical or unsafe. Due to the limited exposure in certain locations, it was sometimes difficult to determine if separate exposures of a dyke represented a single intrusion. Such cases are rare within the dataset and were recorded as separate dykes. In addition to the acquisition of structural data on the dykes data were obtained on the country rock including measurements of bedding in addition to kinematic data from faults.

### 2.2 Subsurface 3-D density and susceptibility models

The 3-D sub-surface density and susceptibility models were derived from the inversion of magnetic and FTG data, respectively, using a probabilistic approach described in [2]. Although many inversions were conducted and presented in [2], the data associated with this article are from the model result that is shown in [1]. The inversions used the FTG data obtained by Bell Geospace, on behalf of Celtic Minerals Ltd. [3] and provided through the Newfoundland and Labrador Department of Natural Resources [4]. These are the same data worked on by [12]. The grid spacing over these data in the study area is 100 m for all tensors. The aeromagnetic data used in this study were extracted from a compilation made by the Newfoundland and Labrador Government Department of Natural Resources [13], and were acquired by the Geological Survey of Canada between 1958 and 1972. The grid spacing of the aeromagnetic data over the study area is 200 m, which is coarser than the FTG data.

## Appendix A Supplementary material

Spreadsheet (.xlsx) with descriptions of supplementary field photographs.

.

Spreadsheet (.xlsx) containing the data for the subsurface models.

.

Spreadsheet (.xlsx) containing the field structural geology data.

.

Higher resolution version of field photographs (.jpg) contained in the Google Earth map file (.kmz).

## Appendix B Supplementary materials

The following KMZ files contain the Google Earth maps of the data acquisition locations and select field photographs.

Map KMZ file containing the Google Earth map of select field photographs.

Map KMZ file containing the Google Earth map of field data acquisition locations.
