# Supplementary material for: Structural geology data and 3-D subsurface models of the Budgell Harbour Stock and associated dykes, Newfoundland, Canada
Source: Data Brief. 2018 Oct 25;21:1690–6. doi: 10.1016/j.dib.2018.10.072 (PMC6249509; doi:10.1016/j.dib.2018.10.072)
Supplement: Supplementary file 1 — Transparency document for the data associated with this article. [file mmc1.doc]

Conflict of Interest and Authorship Conformation Form

Please check the following as appropriate:

- All authors have participated in (a) conception and design, or analysis and interpretation of the data; (b) drafting the article or revising it critically for important intellectual content; and (c) approval of the final version.
- This manuscript has not been submitted to, nor is under review at, another journal or other publishing venue.
- The authors have no affiliation with any organization with a direct or indirect financial interest in the subject matter discussed in the manuscript
- The following authors have affiliations with organizations with direct or indirect financial interest in the subject matter discussed in the manuscript:

Author’s name Affiliation

Alexander L. Peace Memorial University of Newfoundland, Earth Sciences, St. John’s, NL, Canada

J. Kim Welford Memorial University of Newfoundland, Earth Sciences, St. John’s, NL, Canada

Meixia Geng Memorial University of Newfoundland, Earth Sciences, St. John’s, NL, Canada

Institute of Geophysics and Geomatics, China University of Geosciences, China

Hamish Sandeman Department of Natural Resources – Geological Survey, Government of Newfoundland and Labrador, St. John’s, NL, Canada

Brant D. Gaetz Memorial University of Newfoundland, Earth Sciences, St. John’s, NL, Canada

Sarah S. Ryan Memorial University of Newfoundland, Earth Sciences, St. John’s, NL, Canada
