# Supplementary figures and images for: Structural geology data and 3-D subsurface models of the Budgell Harbour Stock and associated dykes, Newfoundland, Canada
Source: Data Brief. 2018 Oct 25;21:1690–6. doi: 10.1016/j.dib.2018.10.072 (PMC6249509; doi:10.1016/j.dib.2018.10.072)

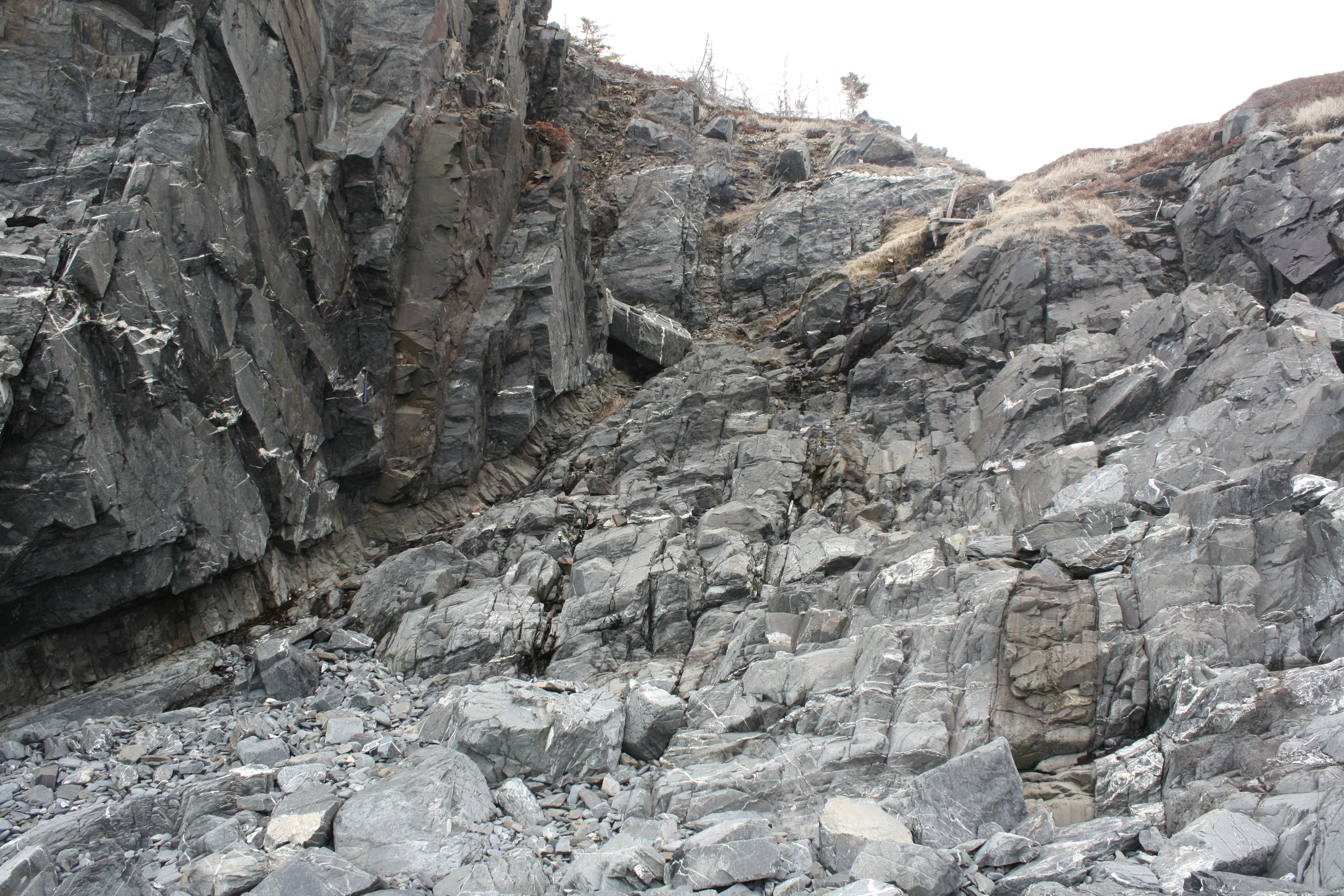

Supplement: Supplementary file 5 — Higher resolution version of field photographs (.jpg) contained in the Google Earth map file (.kmz). [file mmc6.zip › IMG_3862.JPG]

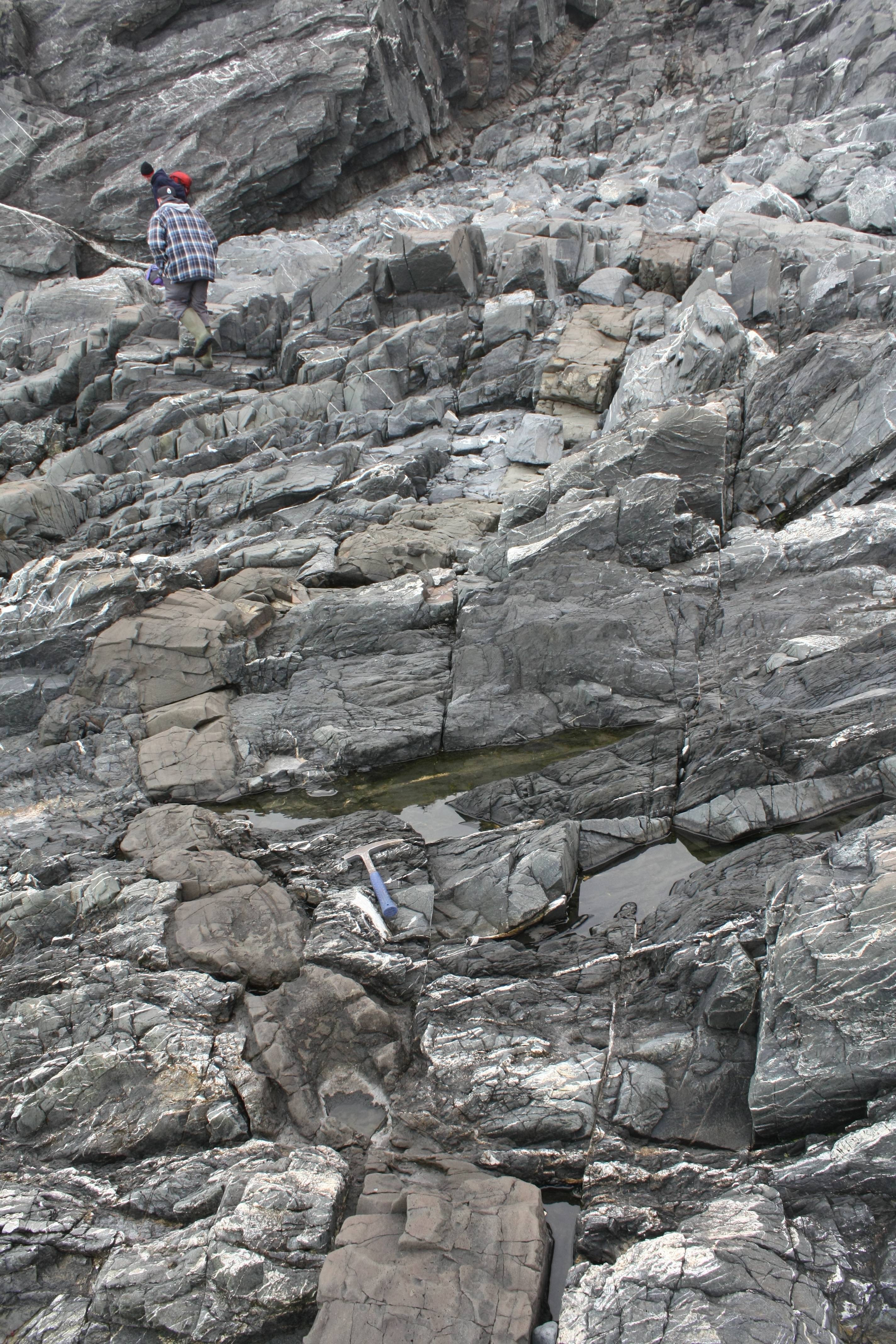

Supplement: Supplementary file 5 — Higher resolution version of field photographs (.jpg) contained in the Google Earth map file (.kmz). [file mmc6.zip › IMG_3879.JPG]

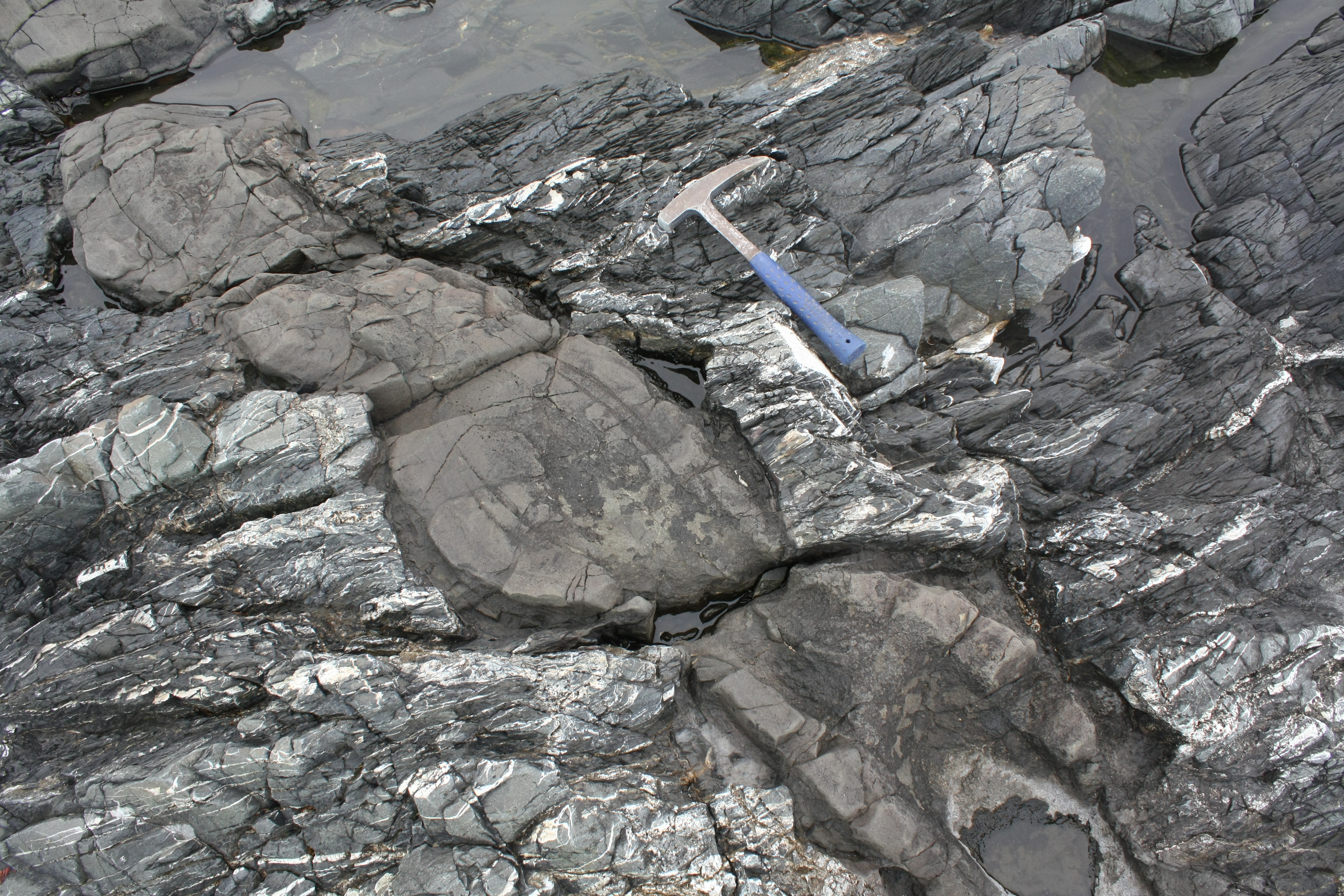

Supplement: Supplementary file 5 — Higher resolution version of field photographs (.jpg) contained in the Google Earth map file (.kmz). [file mmc6.zip › IMG_3882.JPG]

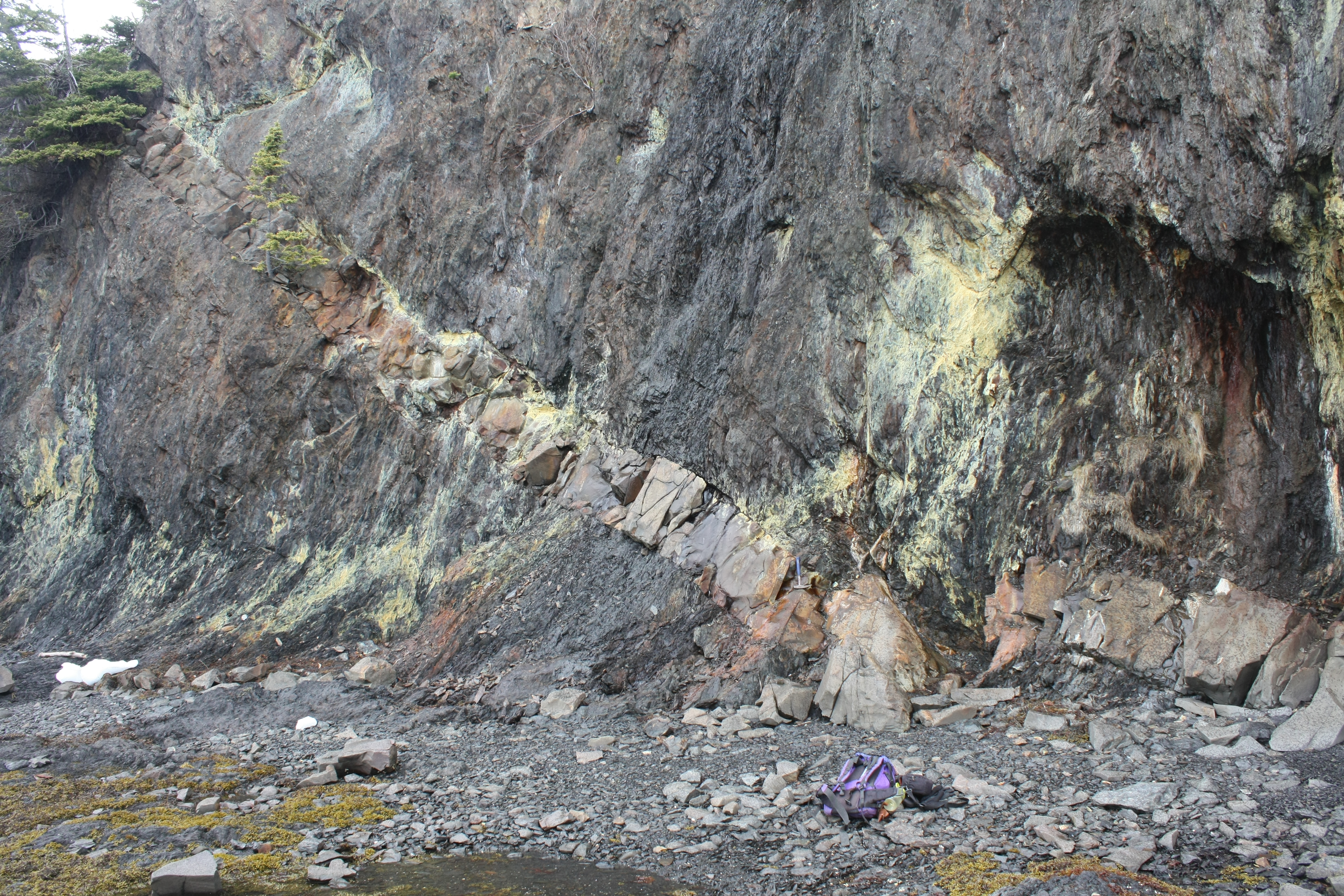

Supplement: Supplementary file 5 — Higher resolution version of field photographs (.jpg) contained in the Google Earth map file (.kmz). [file mmc6.zip › IMG_3885.JPG]

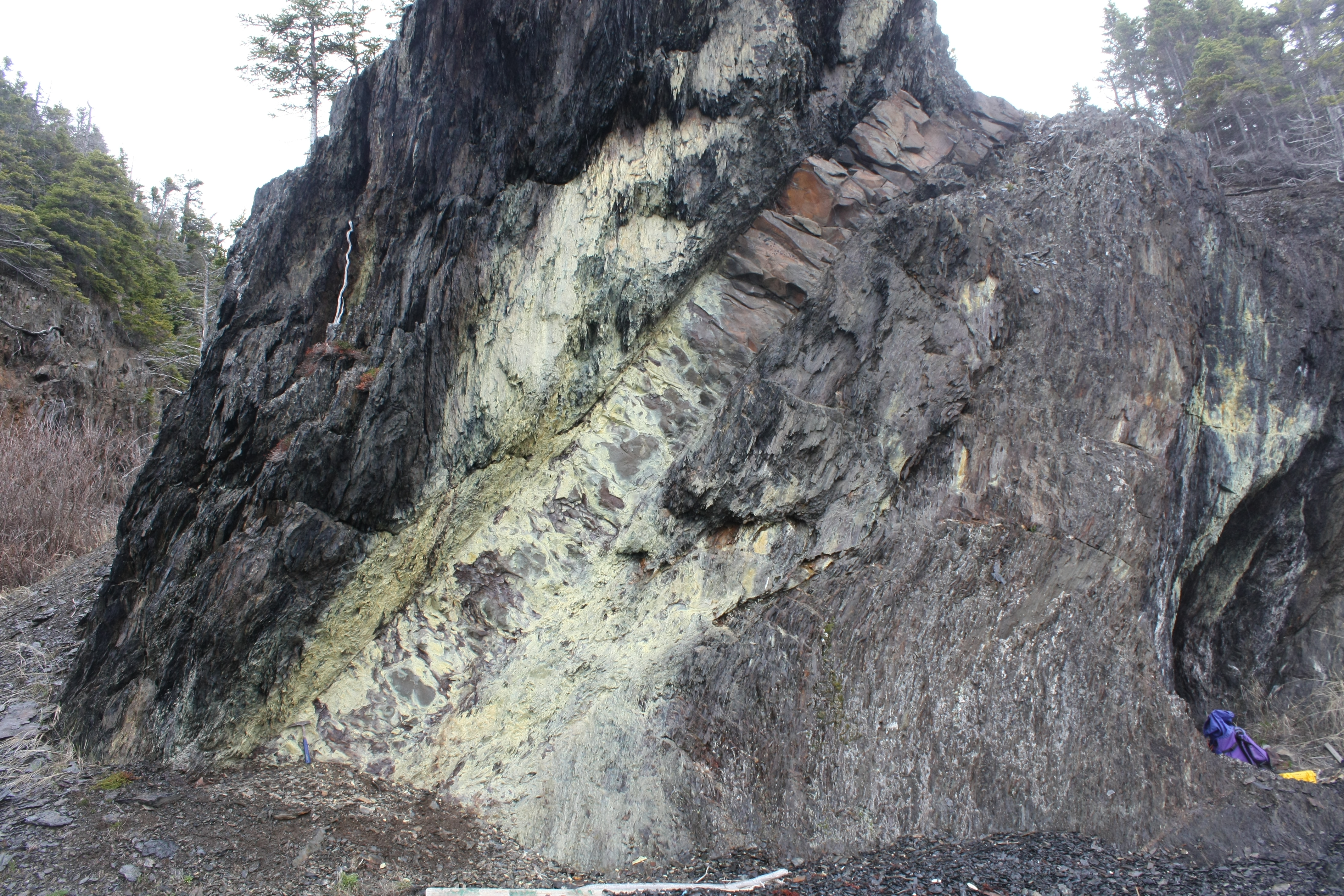

Supplement: Supplementary file 5 — Higher resolution version of field photographs (.jpg) contained in the Google Earth map file (.kmz). [file mmc6.zip › IMG_3911.JPG]

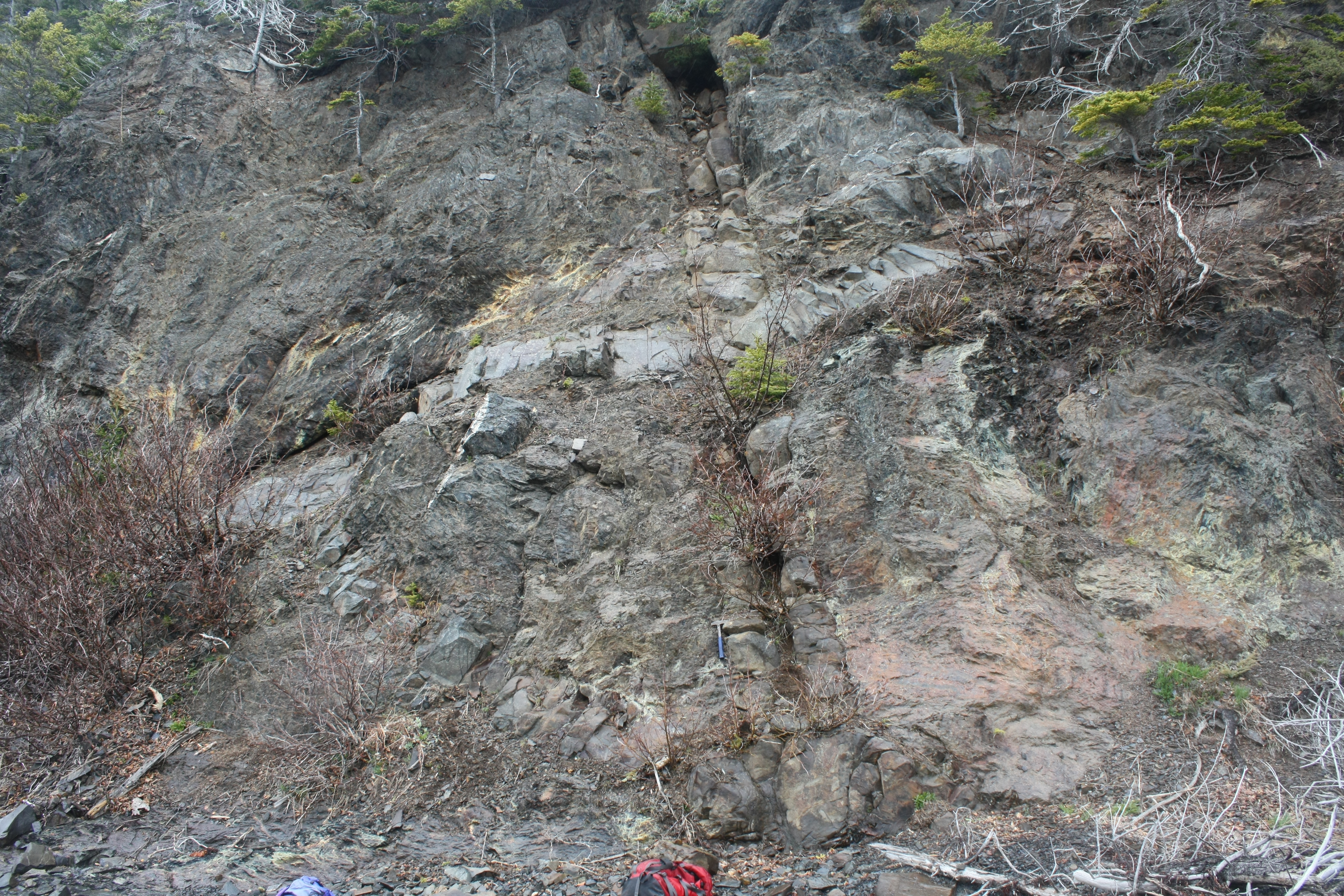

Supplement: Supplementary file 5 — Higher resolution version of field photographs (.jpg) contained in the Google Earth map file (.kmz). [file mmc6.zip › IMG_3959.JPG]

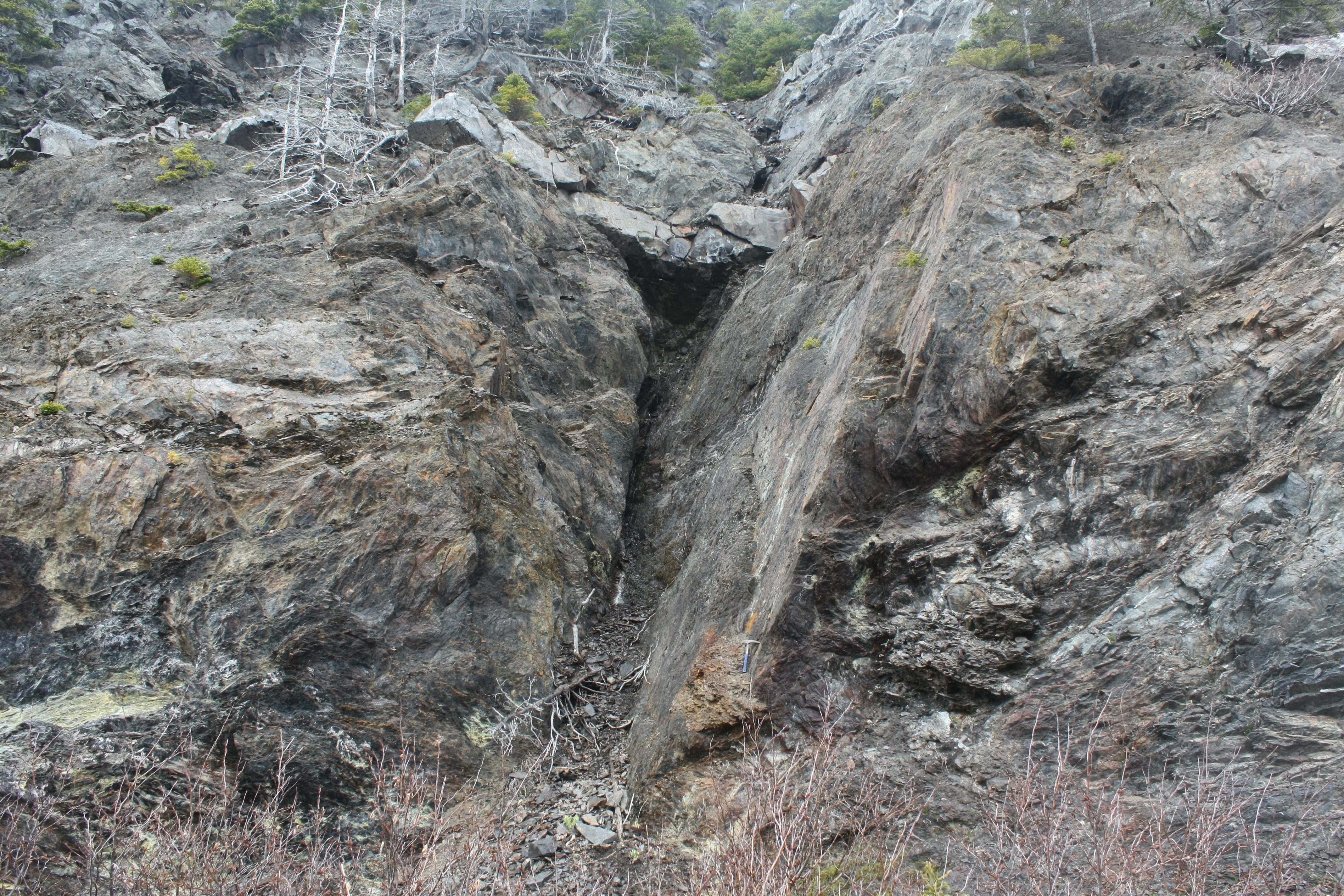

Supplement: Supplementary file 5 — Higher resolution version of field photographs (.jpg) contained in the Google Earth map file (.kmz). [file mmc6.zip › IMG_3965.JPG]

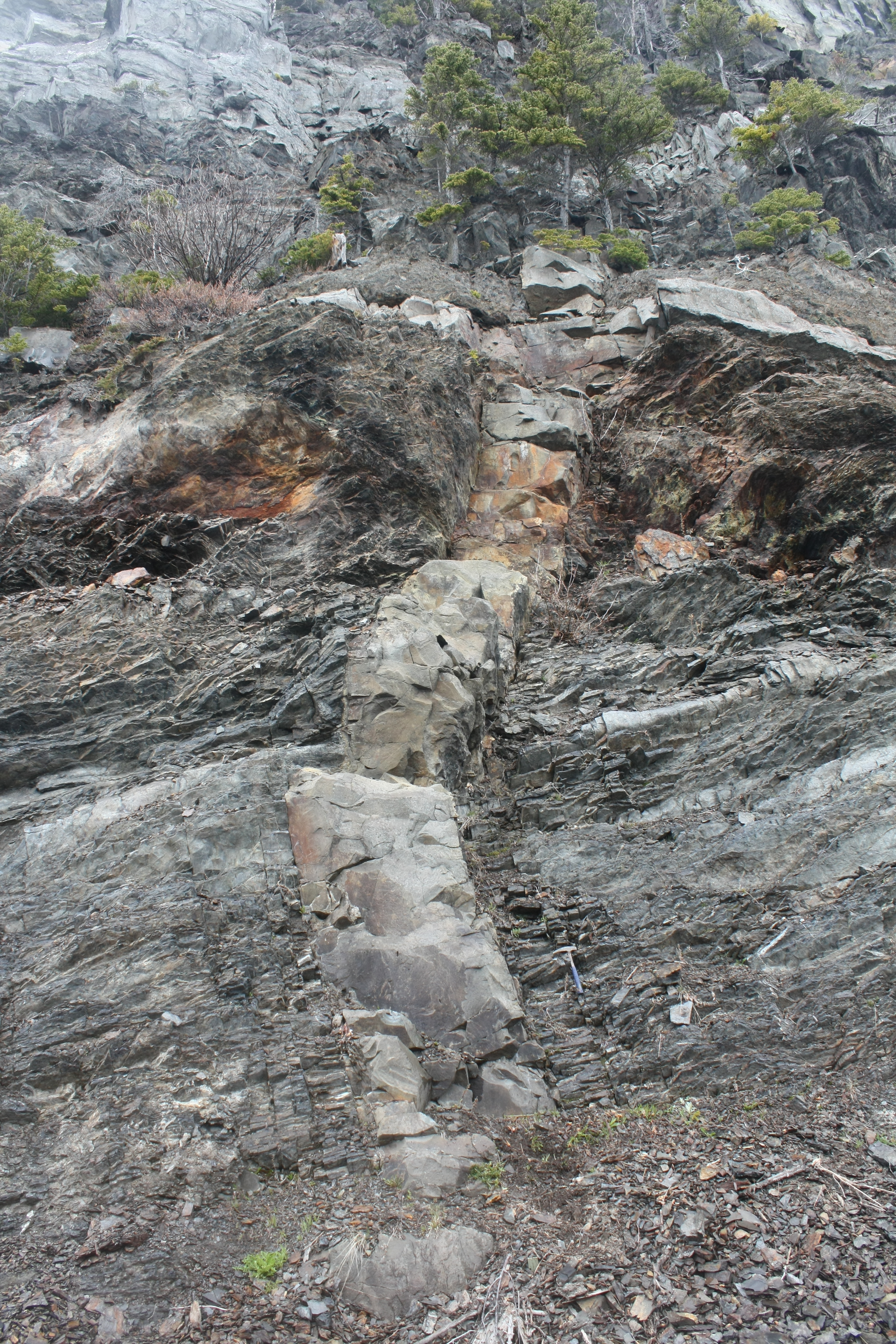

Supplement: Supplementary file 5 — Higher resolution version of field photographs (.jpg) contained in the Google Earth map file (.kmz). [file mmc6.zip › IMG_3971.JPG]

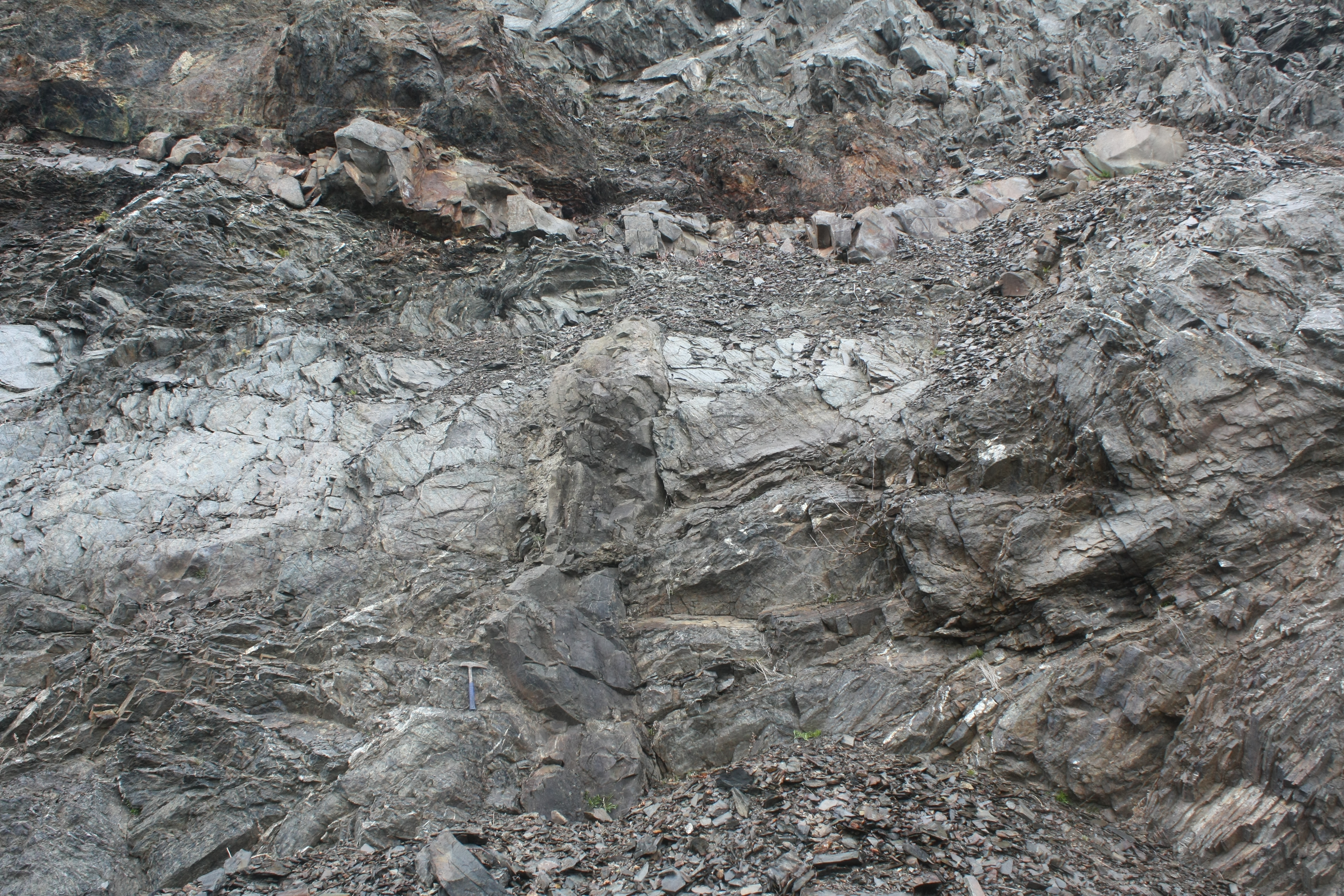

Supplement: Supplementary file 5 — Higher resolution version of field photographs (.jpg) contained in the Google Earth map file (.kmz). [file mmc6.zip › IMG_3982.JPG]

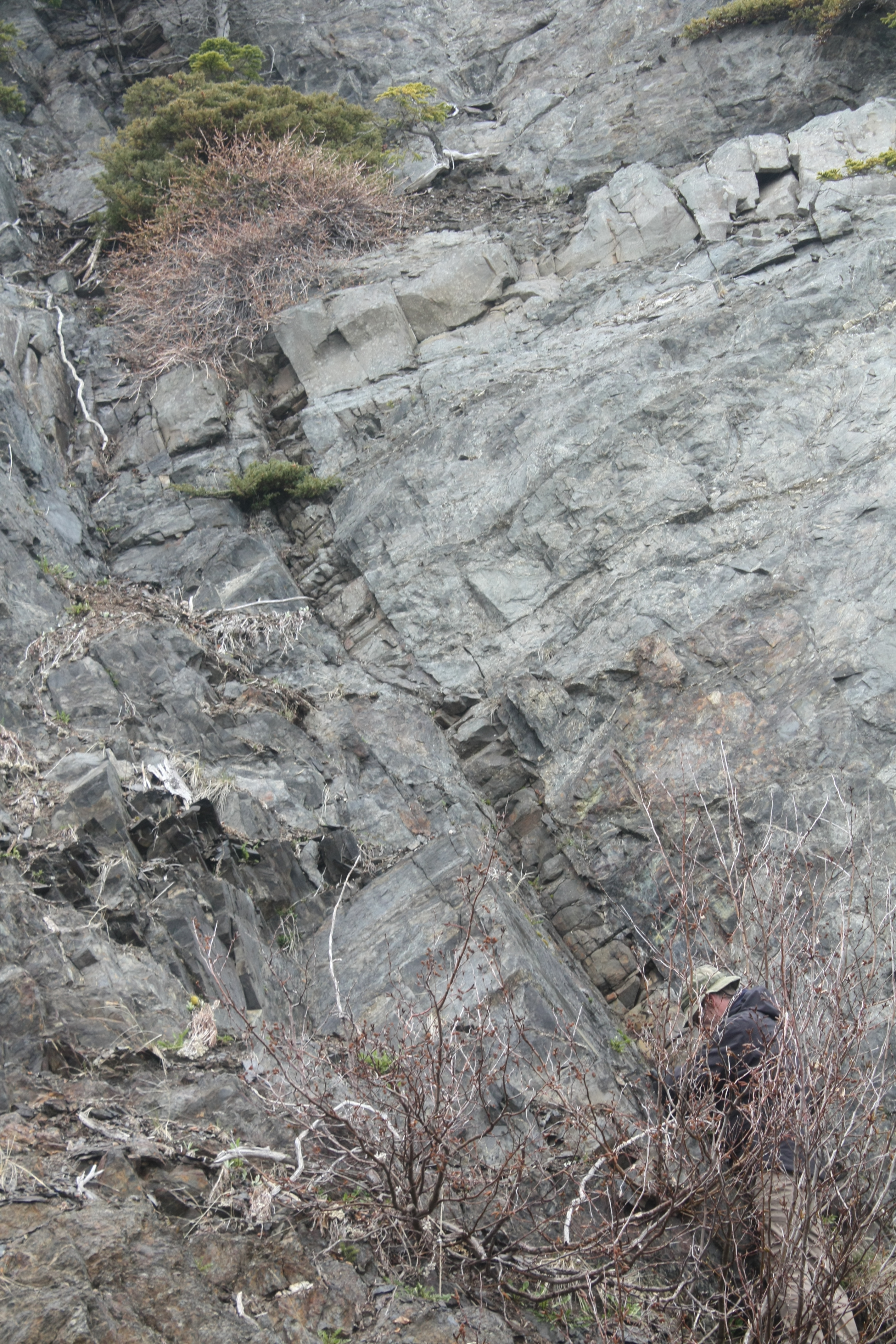

Supplement: Supplementary file 5 — Higher resolution version of field photographs (.jpg) contained in the Google Earth map file (.kmz). [file mmc6.zip › IMG_3986.JPG]

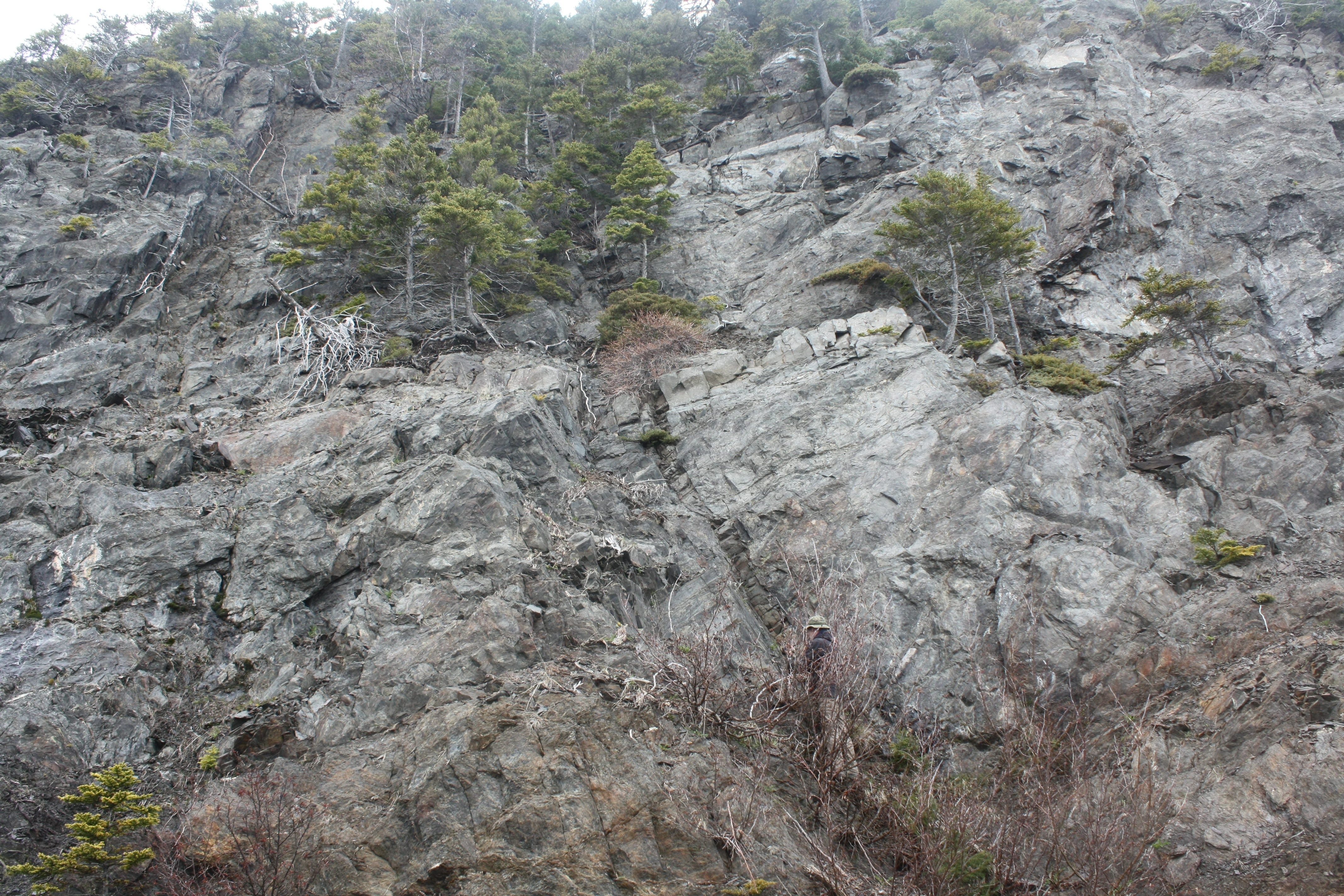

Supplement: Supplementary file 5 — Higher resolution version of field photographs (.jpg) contained in the Google Earth map file (.kmz). [file mmc6.zip › IMG_3990.JPG]

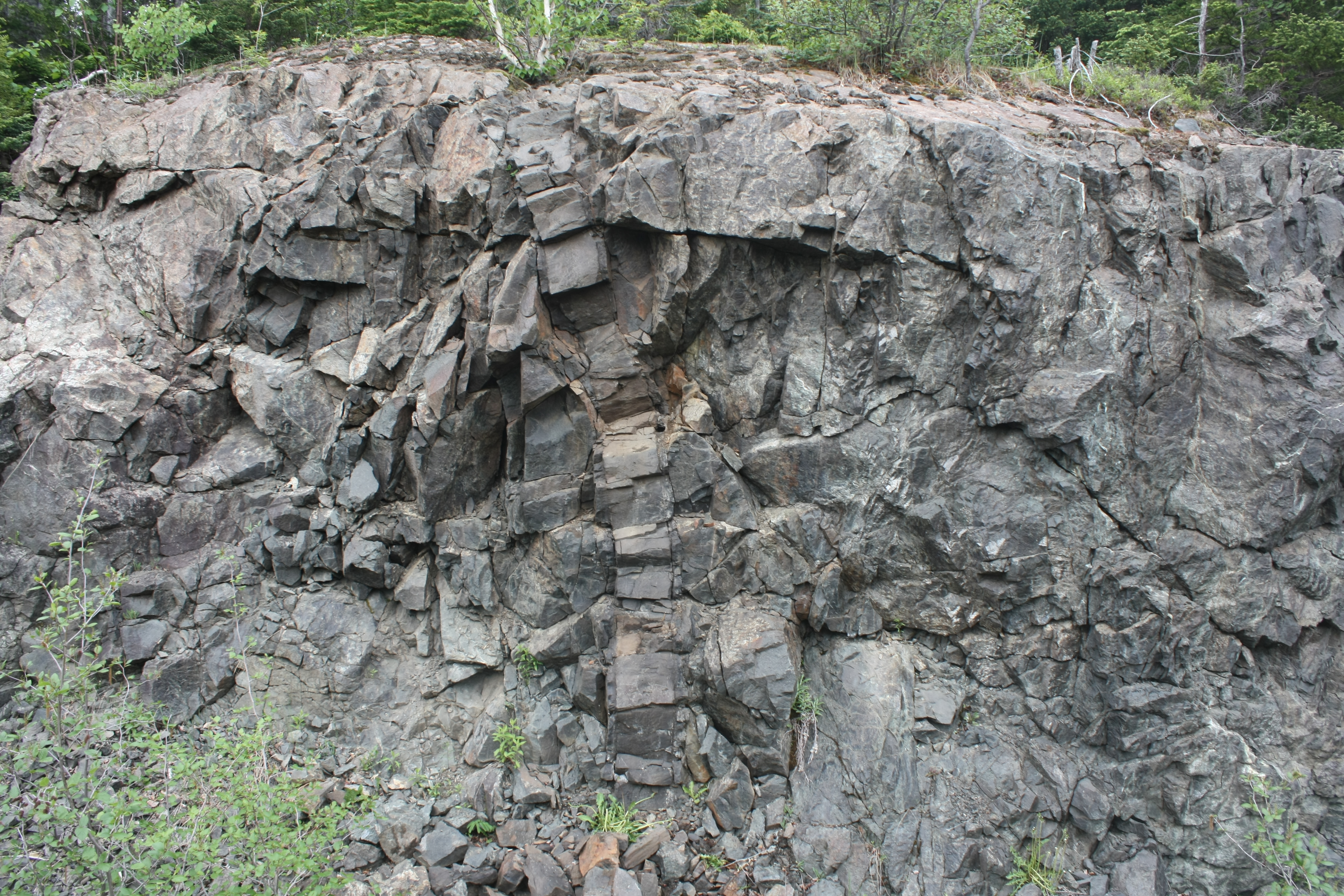

Supplement: Supplementary file 5 — Higher resolution version of field photographs (.jpg) contained in the Google Earth map file (.kmz). [file mmc6.zip › IMG_4051.JPG]

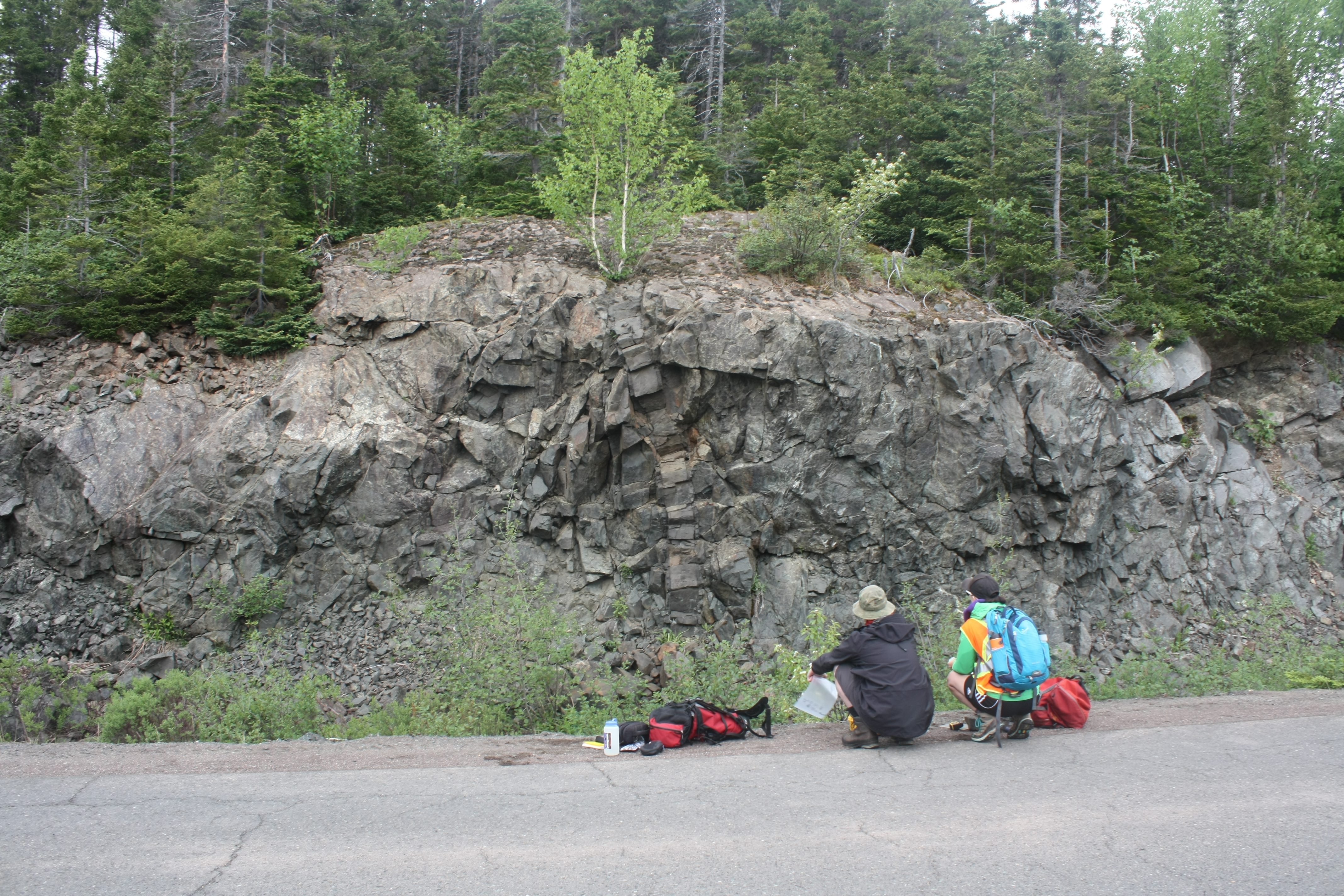

Supplement: Supplementary file 5 — Higher resolution version of field photographs (.jpg) contained in the Google Earth map file (.kmz). [file mmc6.zip › IMG_4054.JPG]

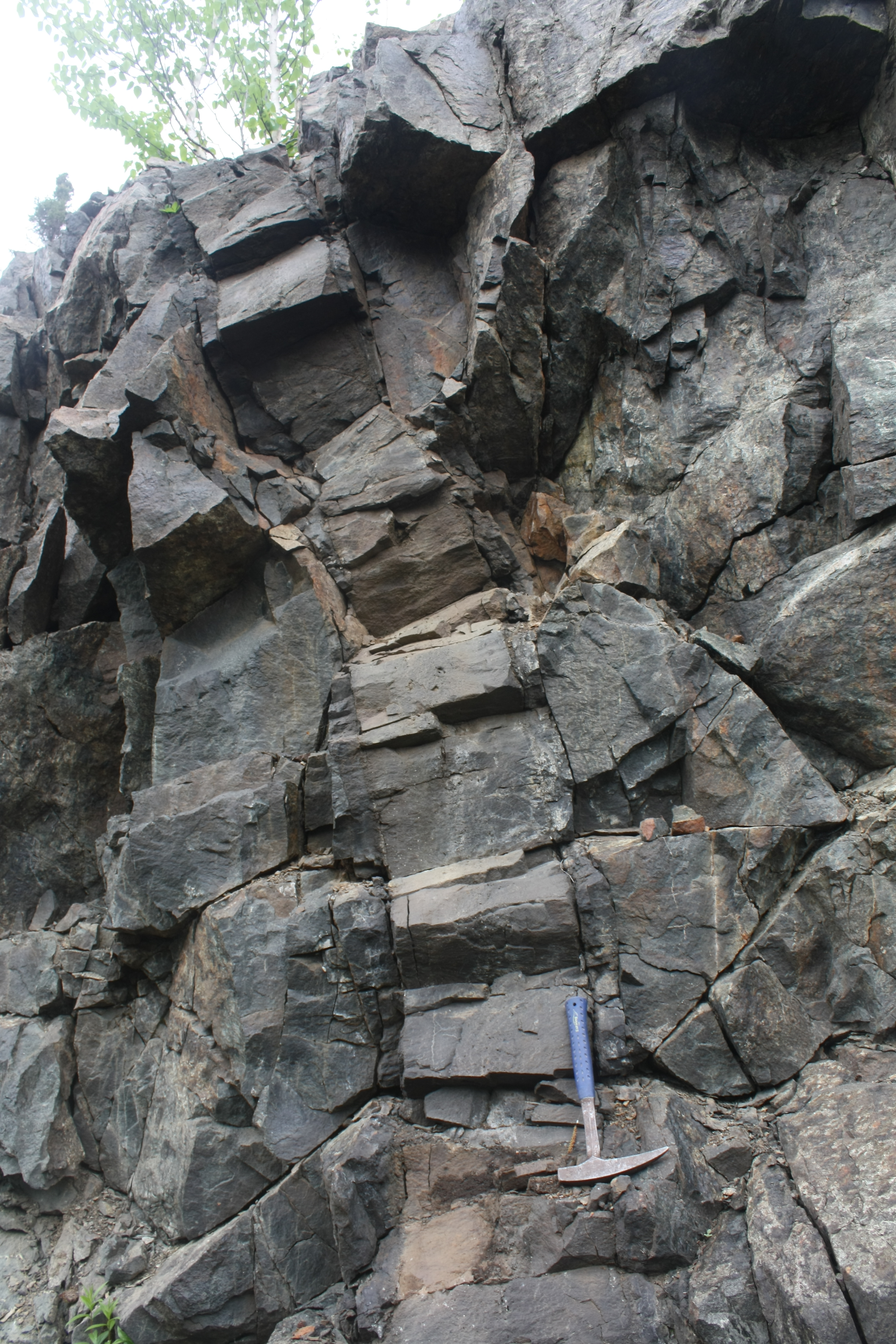

Supplement: Supplementary file 5 — Higher resolution version of field photographs (.jpg) contained in the Google Earth map file (.kmz). [file mmc6.zip › IMG_4059.JPG]

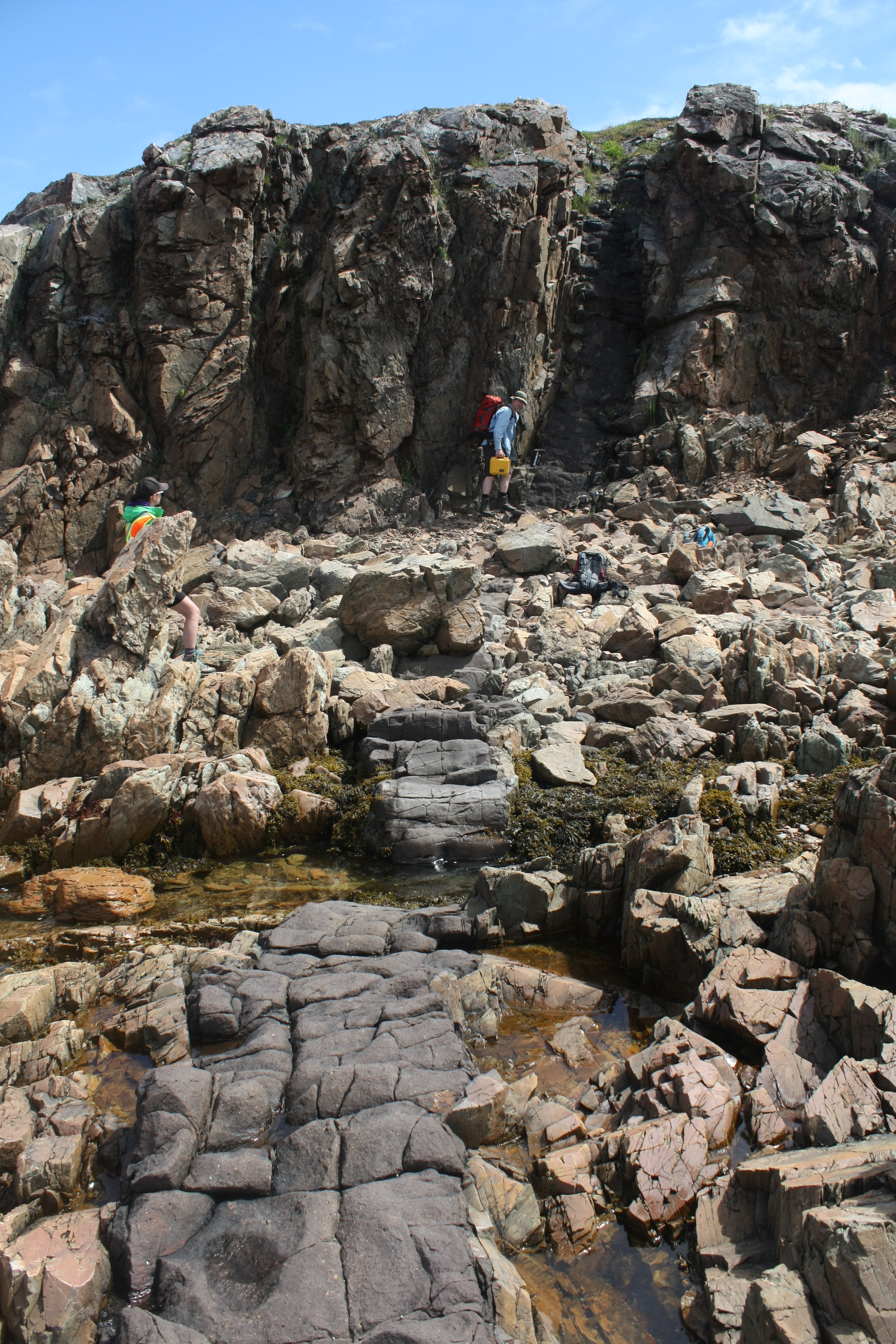

Supplement: Supplementary file 5 — Higher resolution version of field photographs (.jpg) contained in the Google Earth map file (.kmz). [file mmc6.zip › IMG_4068.JPG]

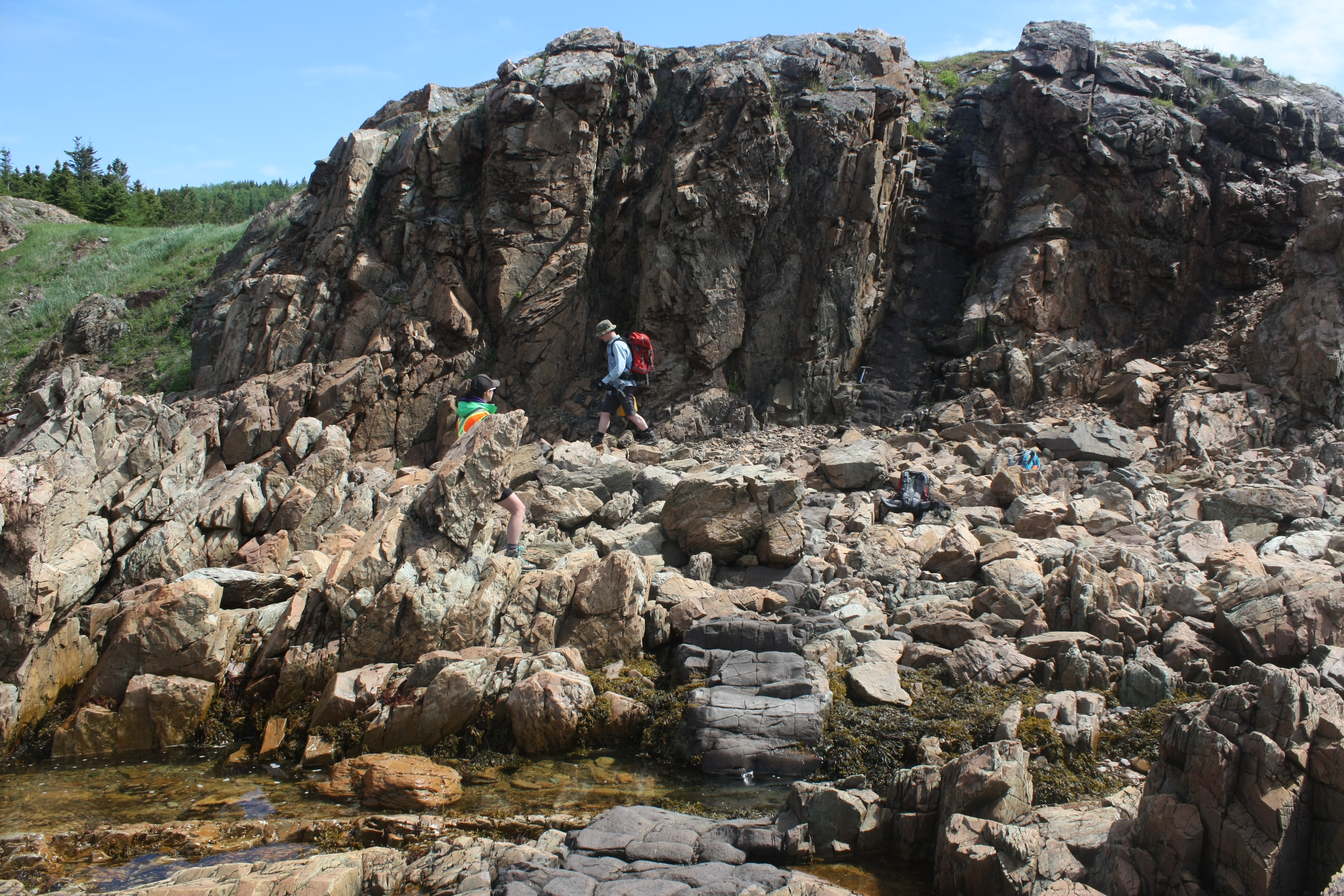

Supplement: Supplementary file 5 — Higher resolution version of field photographs (.jpg) contained in the Google Earth map file (.kmz). [file mmc6.zip › IMG_4070.JPG]

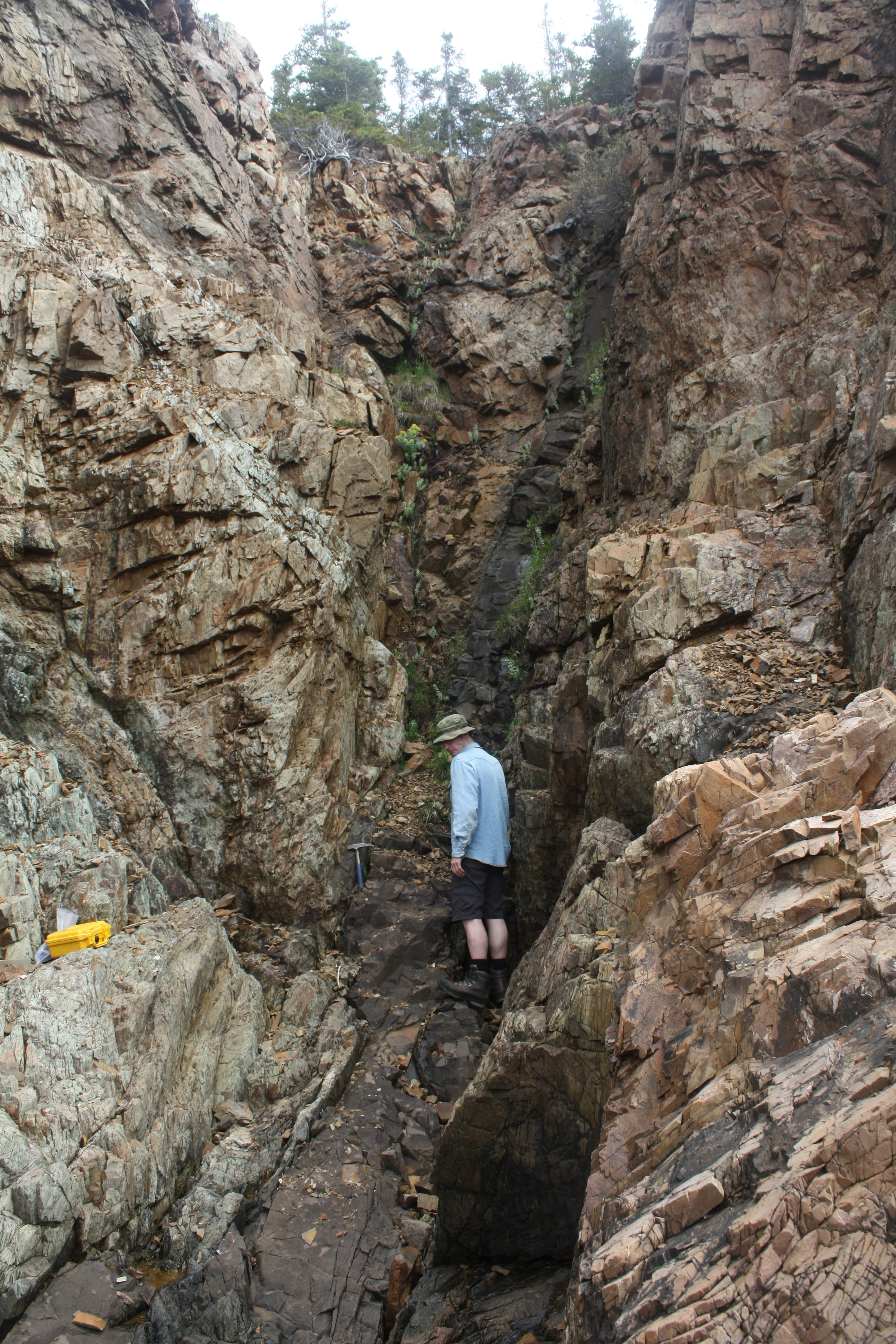

Supplement: Supplementary file 5 — Higher resolution version of field photographs (.jpg) contained in the Google Earth map file (.kmz). [file mmc6.zip › IMG_4093.JPG]

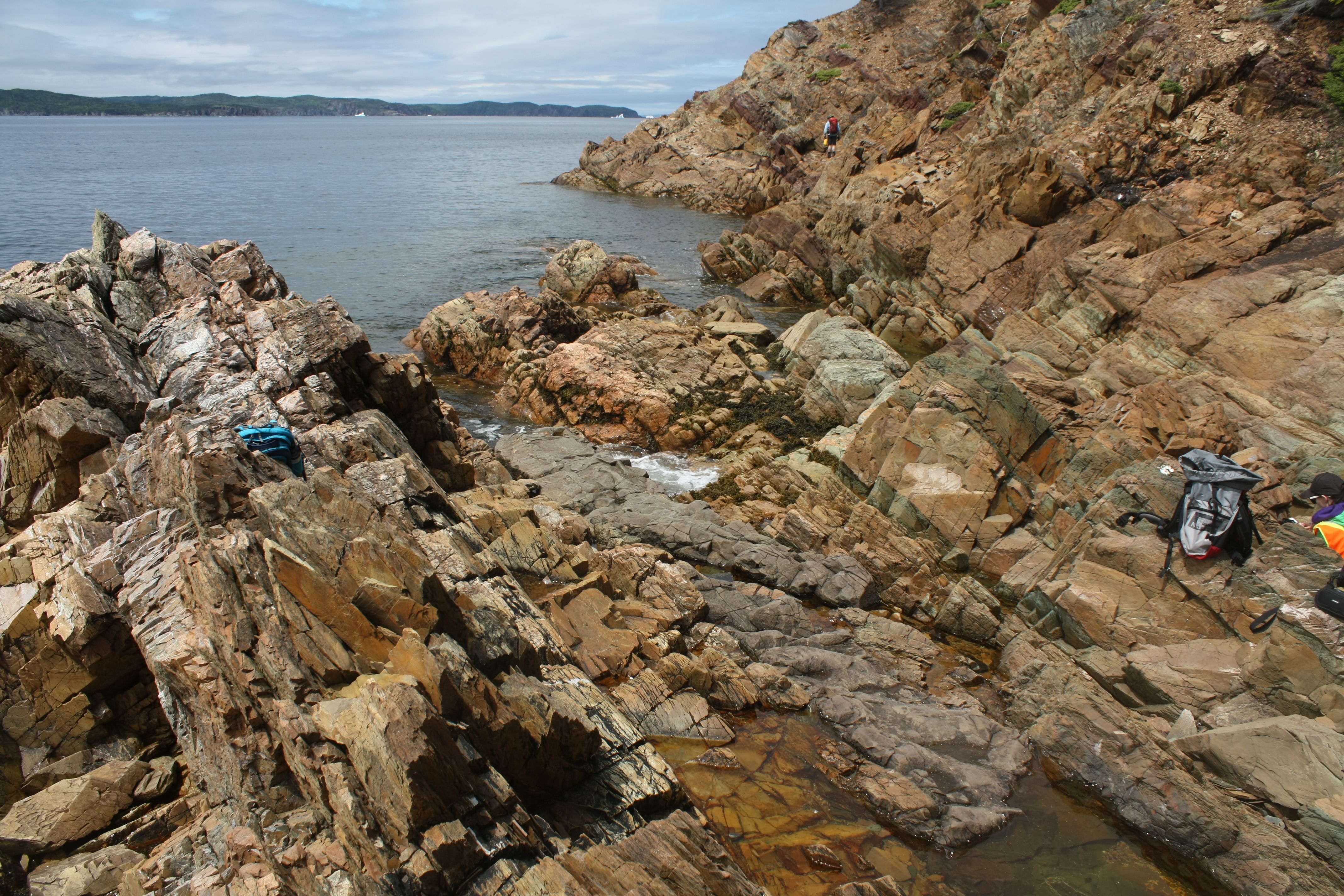

Supplement: Supplementary file 5 — Higher resolution version of field photographs (.jpg) contained in the Google Earth map file (.kmz). [file mmc6.zip › IMG_4103.JPG]

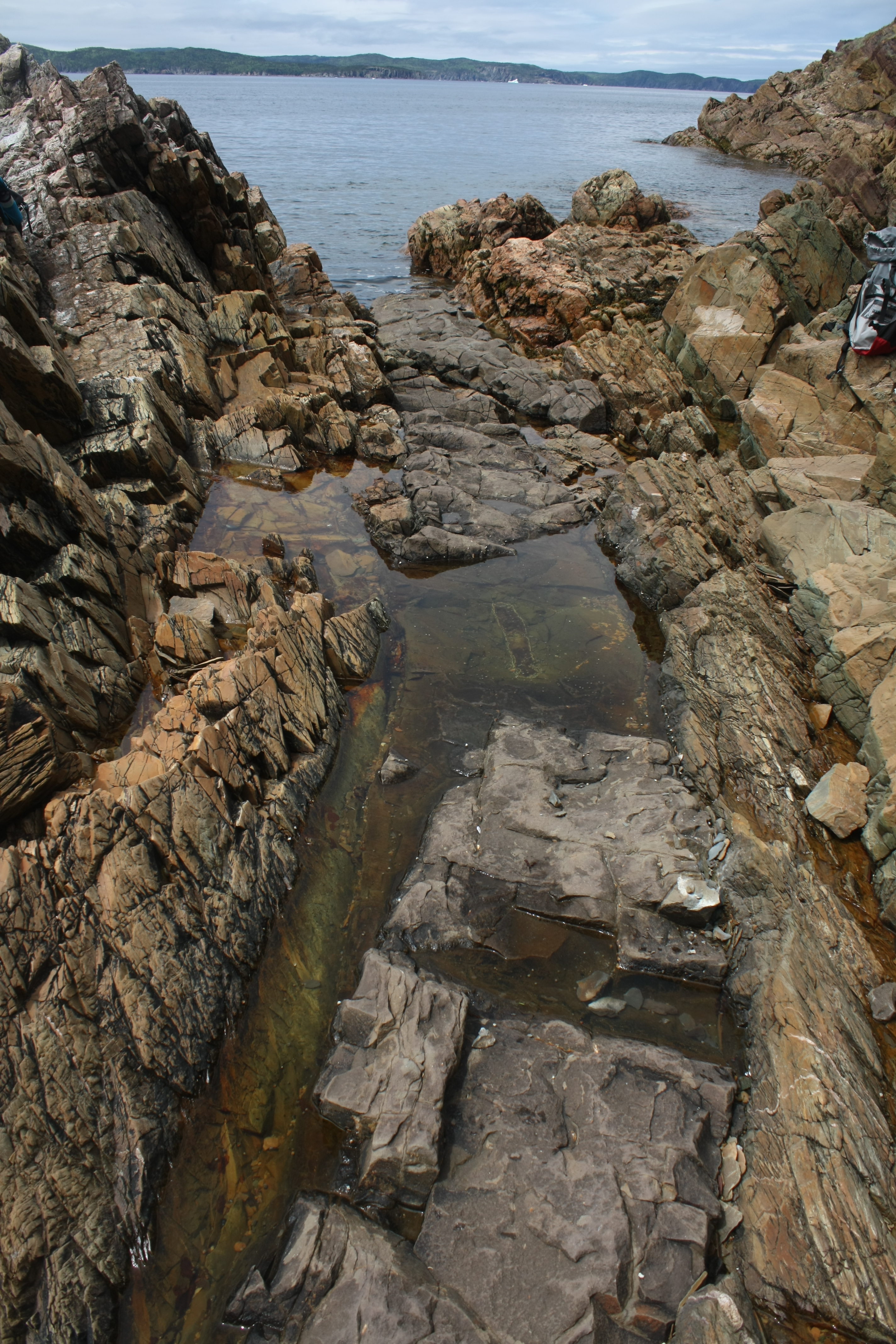

Supplement: Supplementary file 5 — Higher resolution version of field photographs (.jpg) contained in the Google Earth map file (.kmz). [file mmc6.zip › IMG_4106.JPG]

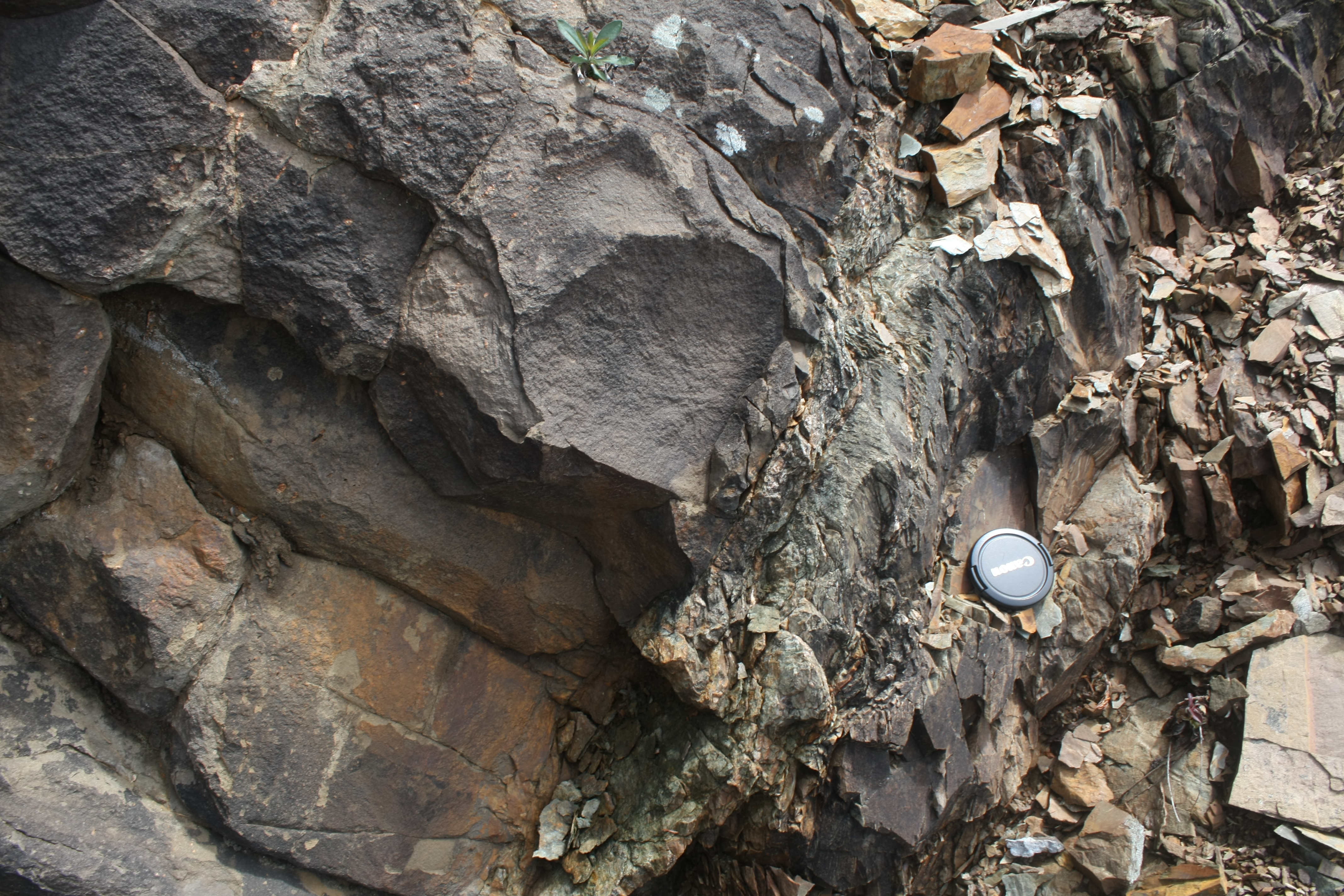

Supplement: Supplementary file 5 — Higher resolution version of field photographs (.jpg) contained in the Google Earth map file (.kmz). [file mmc6.zip › IMG_4113.JPG]

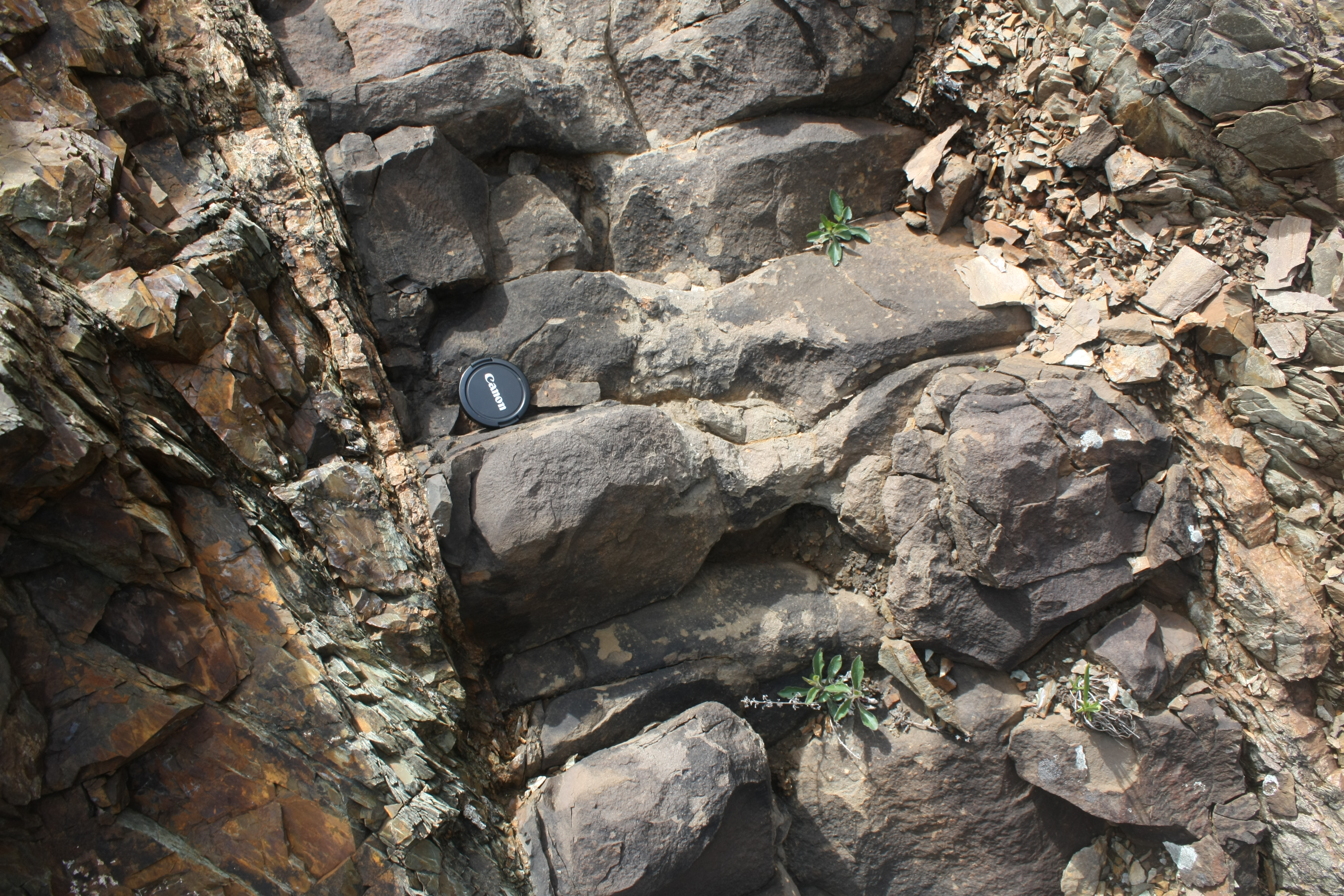

Supplement: Supplementary file 5 — Higher resolution version of field photographs (.jpg) contained in the Google Earth map file (.kmz). [file mmc6.zip › IMG_4117.JPG]

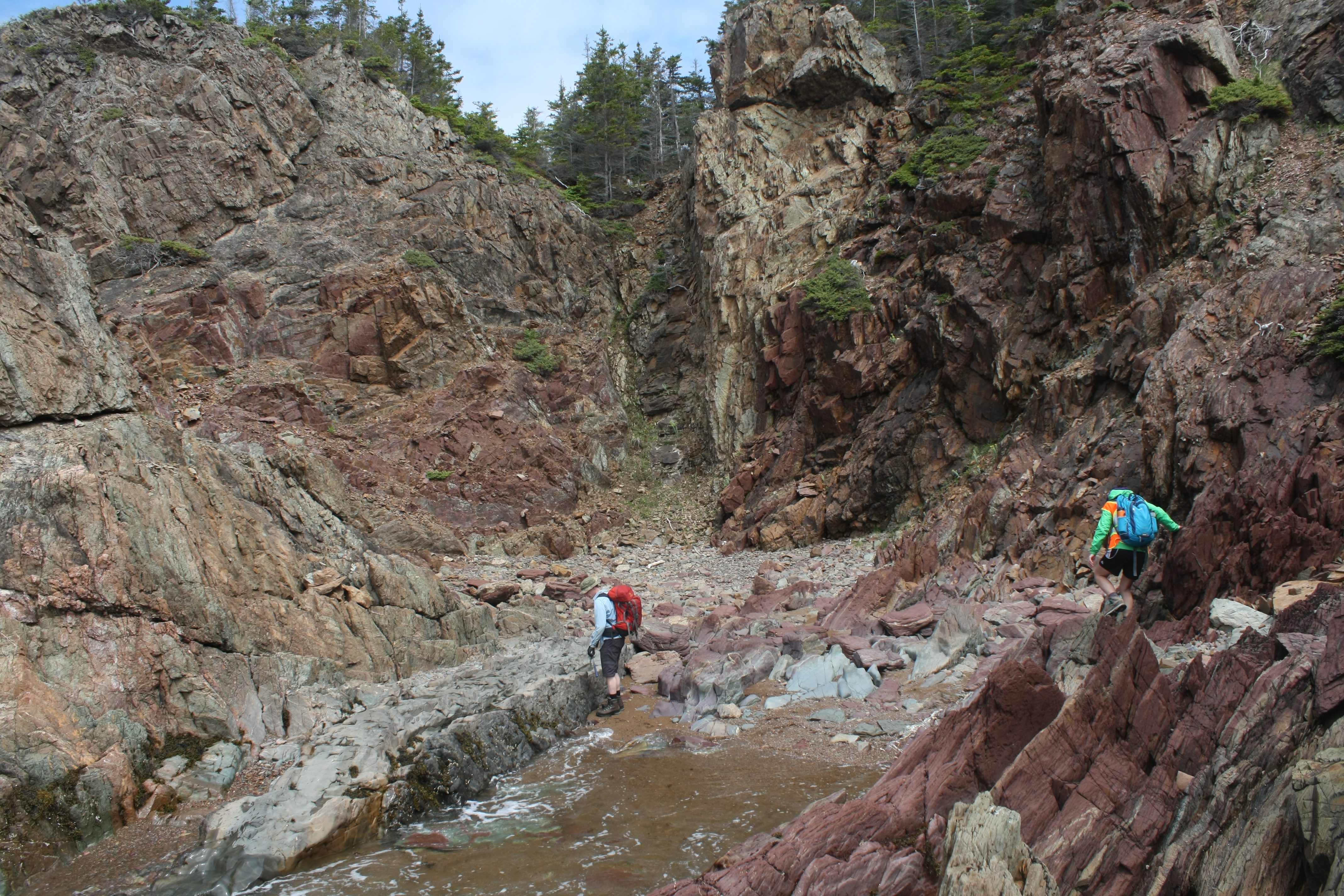

Supplement: Supplementary file 5 — Higher resolution version of field photographs (.jpg) contained in the Google Earth map file (.kmz). [file mmc6.zip › IMG_4123.JPG]

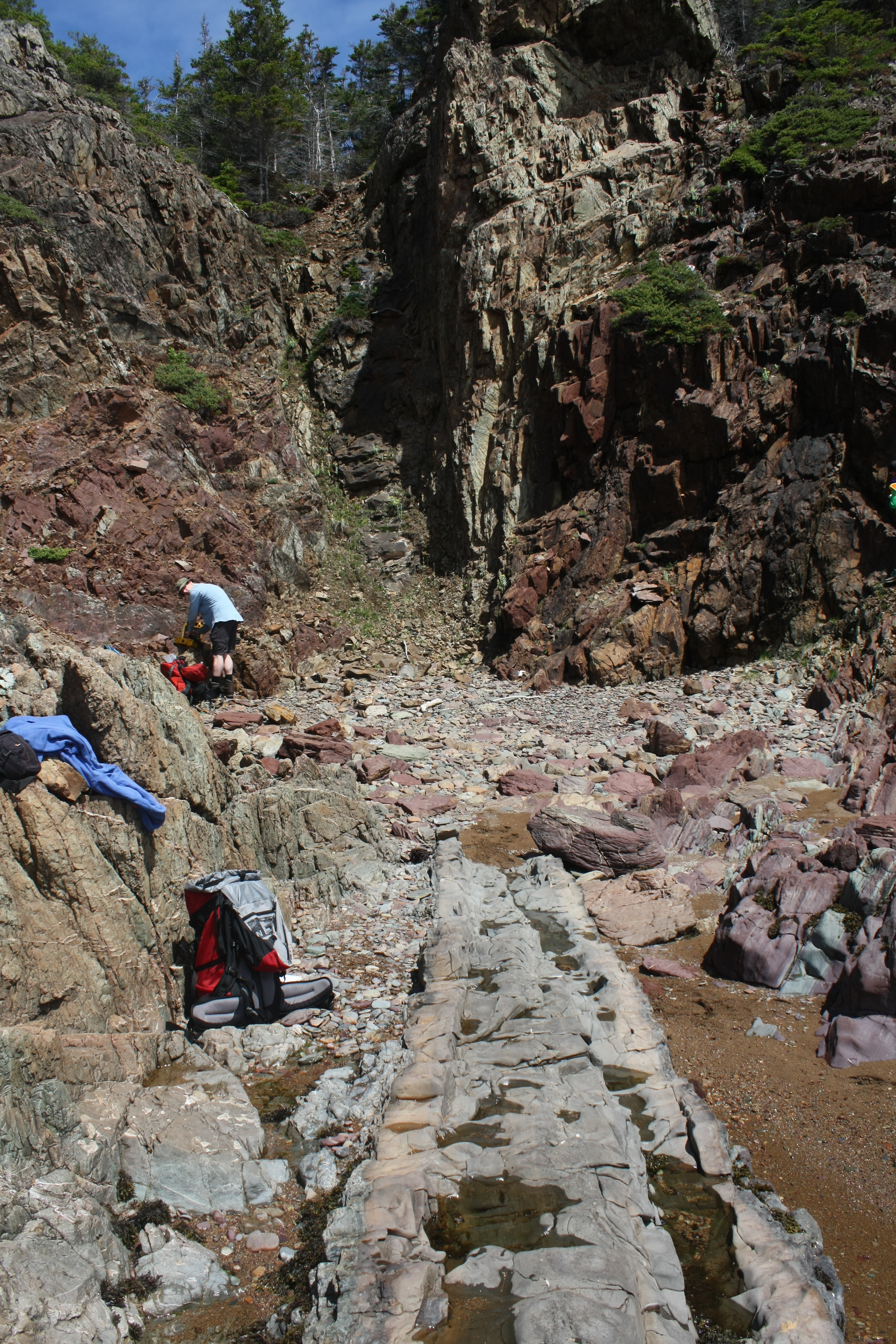

Supplement: Supplementary file 5 — Higher resolution version of field photographs (.jpg) contained in the Google Earth map file (.kmz). [file mmc6.zip › IMG_4129.JPG]

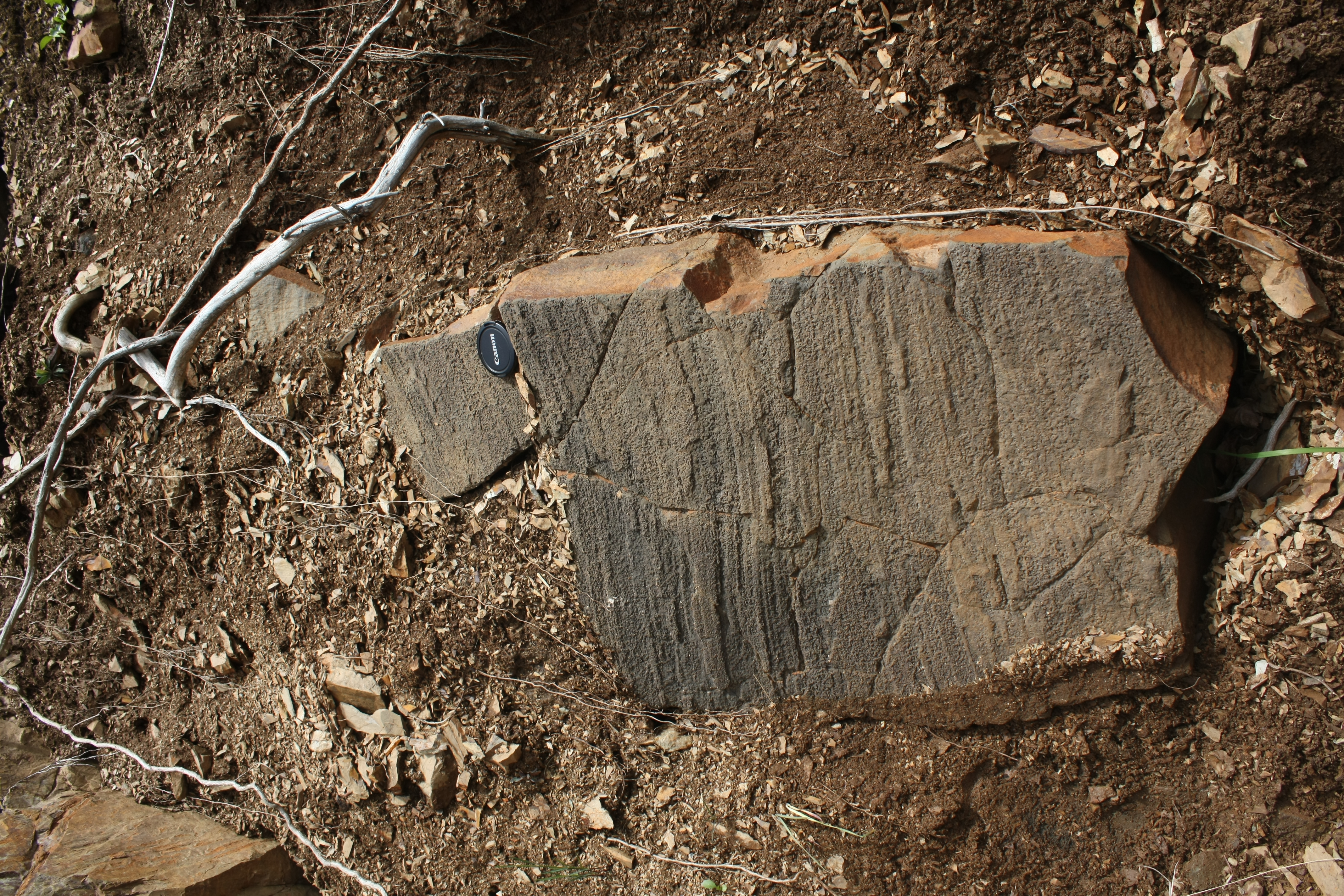

Supplement: Supplementary file 5 — Higher resolution version of field photographs (.jpg) contained in the Google Earth map file (.kmz). [file mmc6.zip › IMG_4139.JPG]

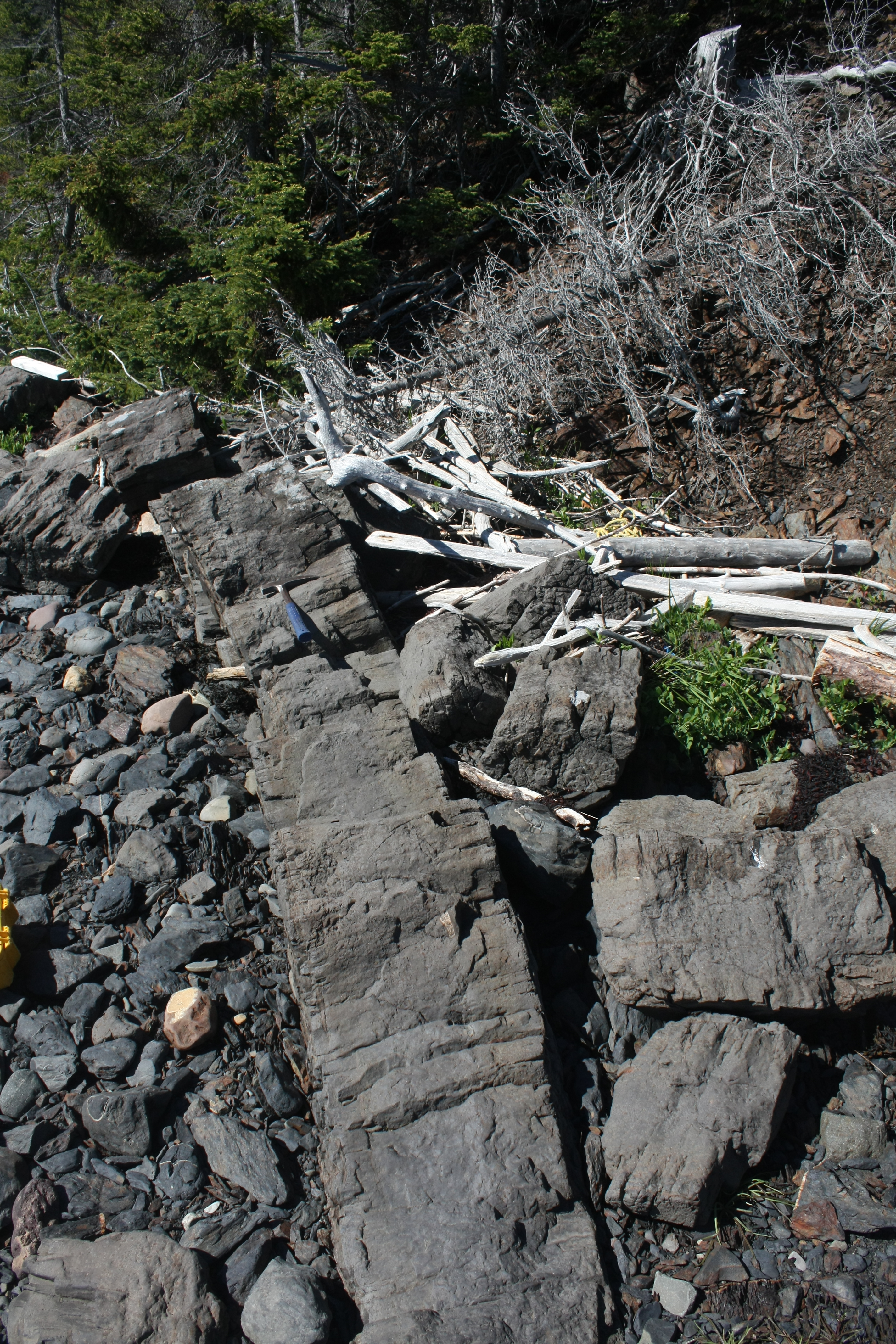

Supplement: Supplementary file 5 — Higher resolution version of field photographs (.jpg) contained in the Google Earth map file (.kmz). [file mmc6.zip › IMG_4174.JPG]

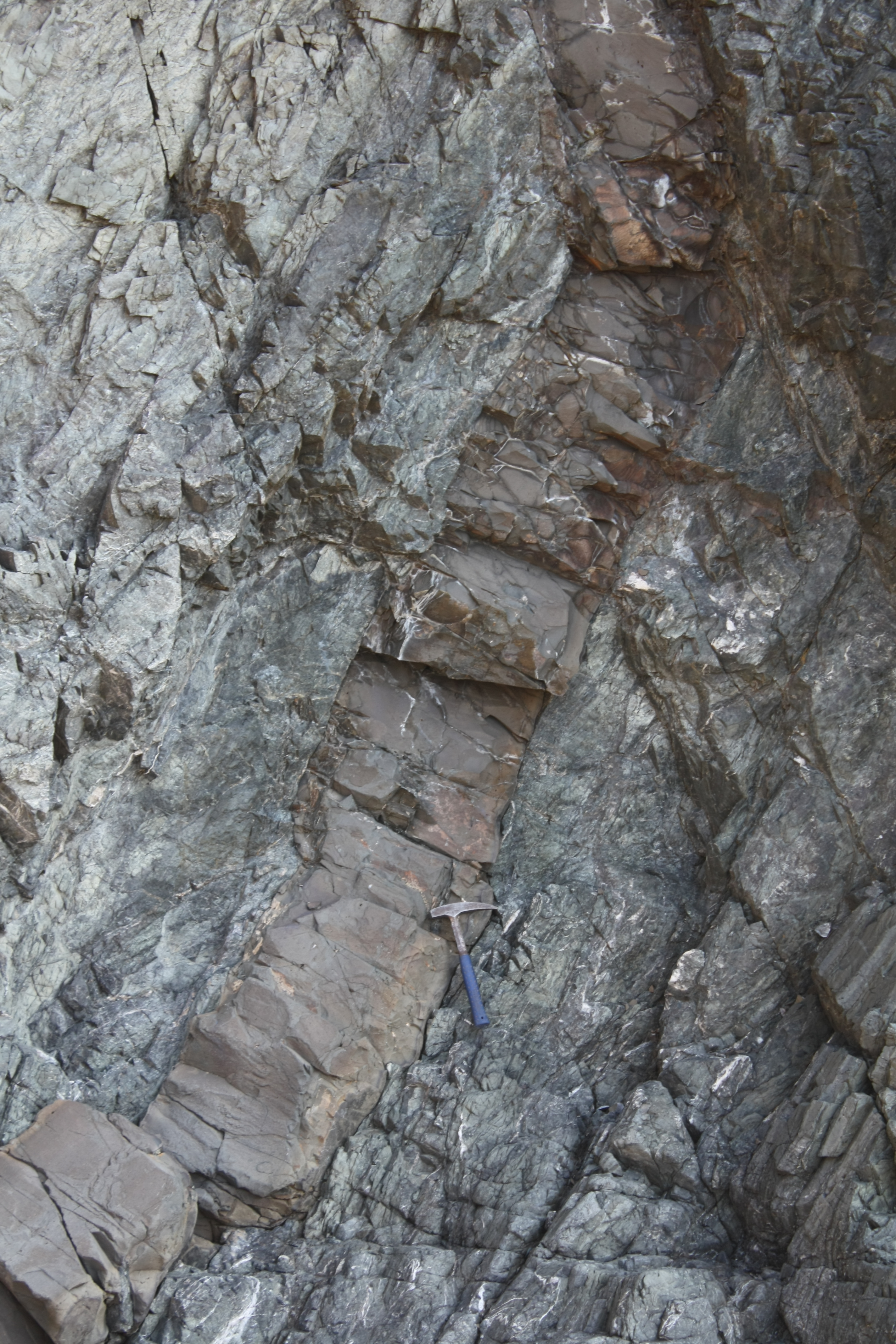

Supplement: Supplementary file 5 — Higher resolution version of field photographs (.jpg) contained in the Google Earth map file (.kmz). [file mmc6.zip › IMG_4541.JPG]

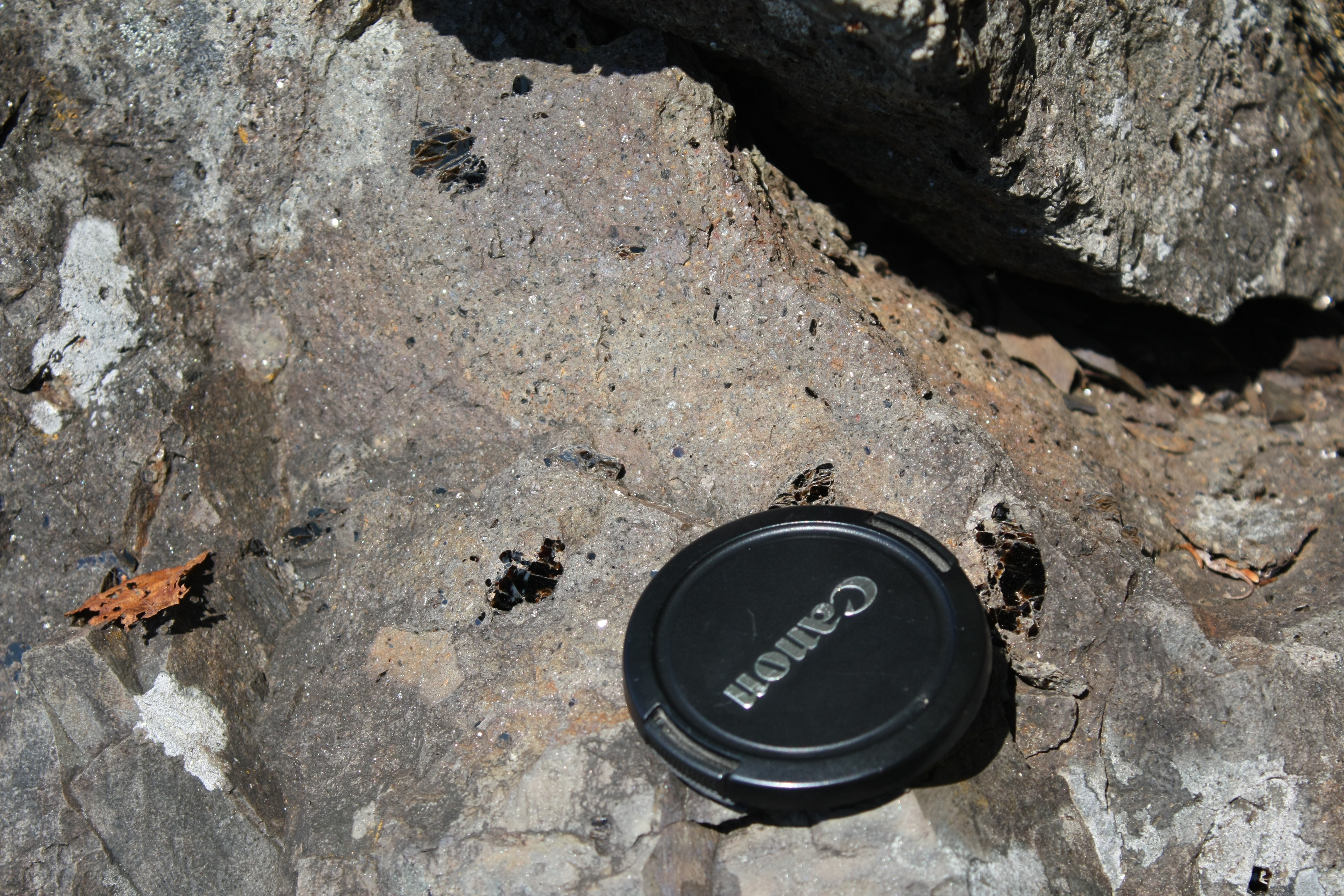

Supplement: Supplementary file 5 — Higher resolution version of field photographs (.jpg) contained in the Google Earth map file (.kmz). [file mmc6.zip › IMG_4938.JPG]

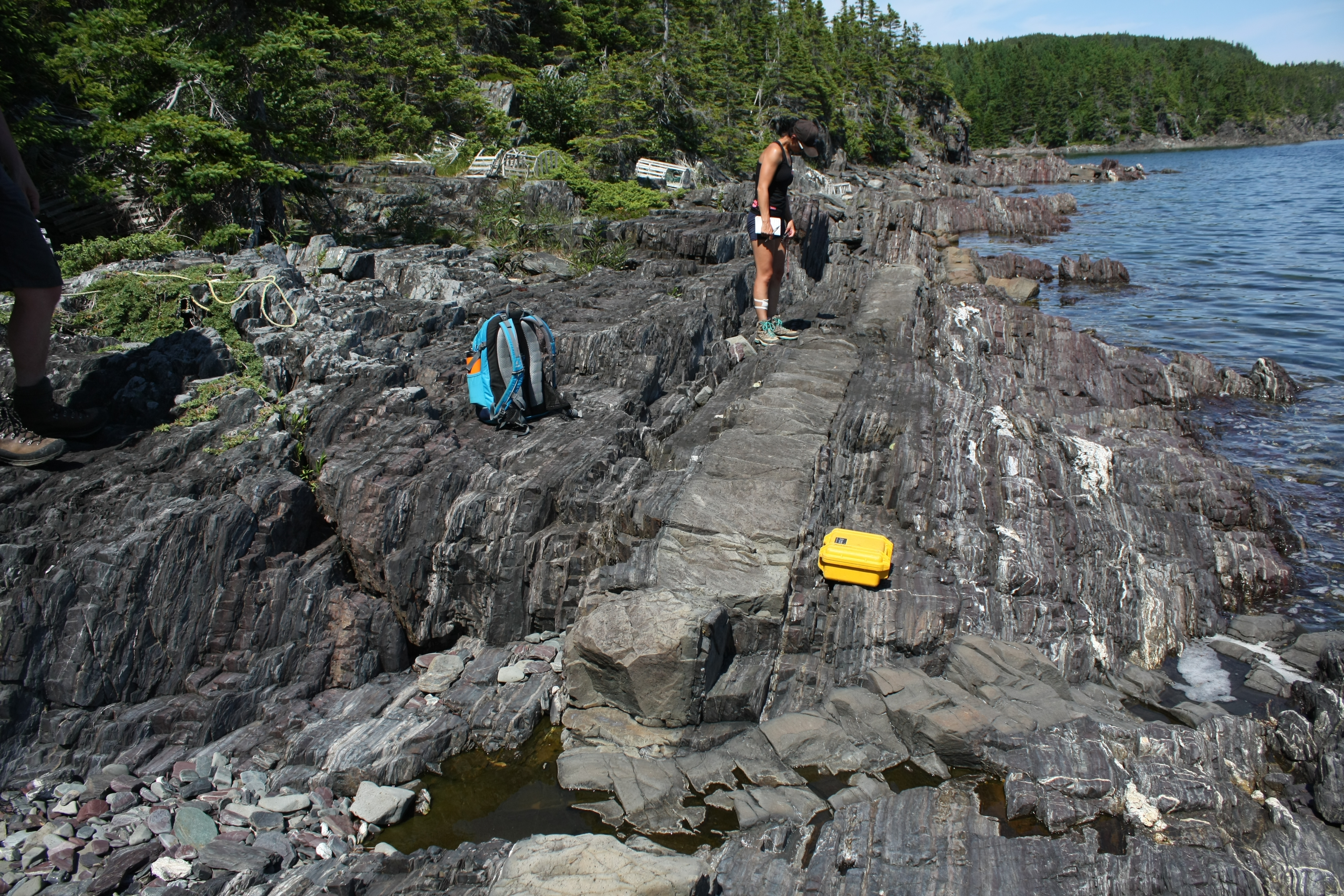

Supplement: Supplementary file 5 — Higher resolution version of field photographs (.jpg) contained in the Google Earth map file (.kmz). [file mmc6.zip › IMG_5050.JPG]

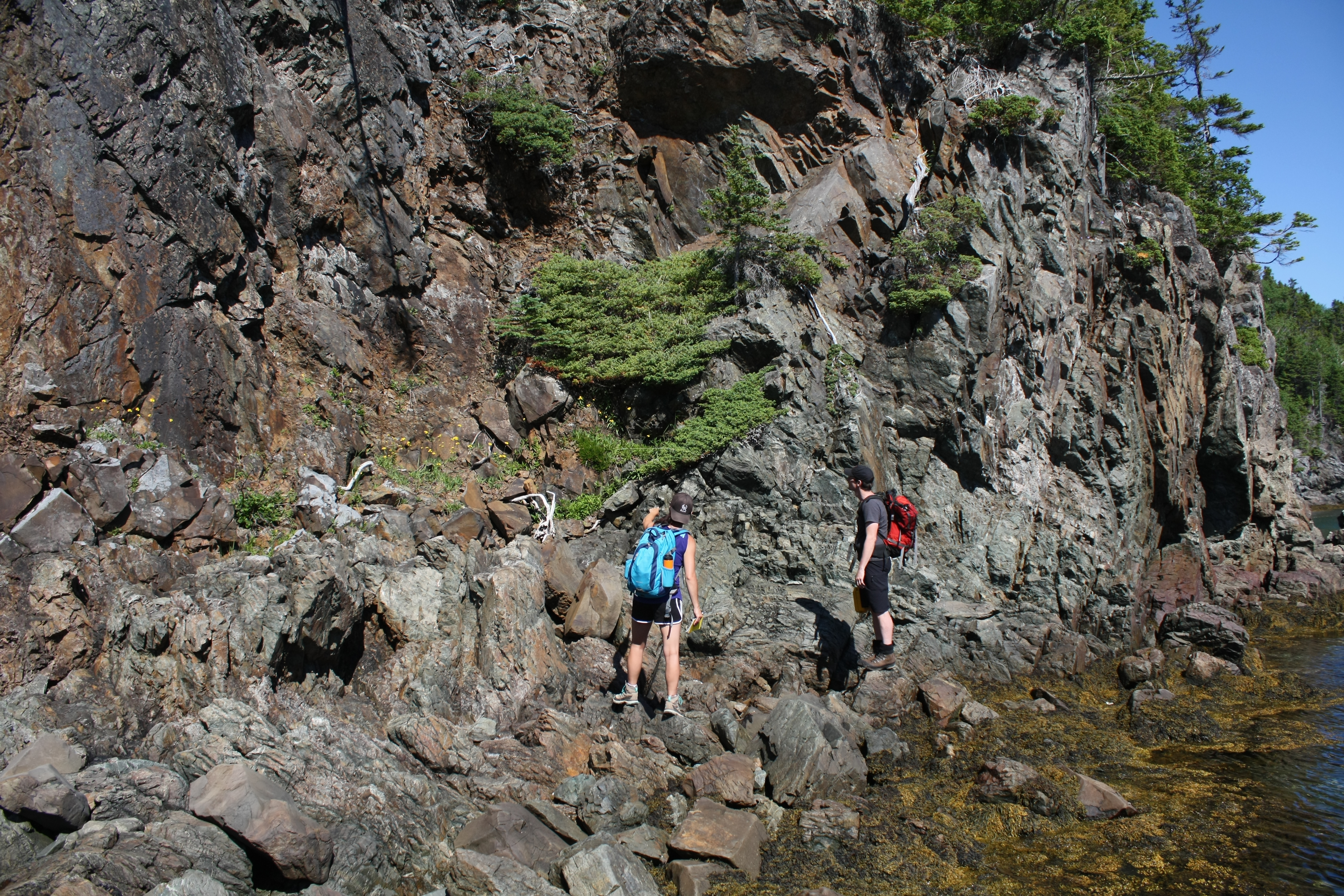

Supplement: Supplementary file 5 — Higher resolution version of field photographs (.jpg) contained in the Google Earth map file (.kmz). [file mmc6.zip › IMG_5125.JPG]

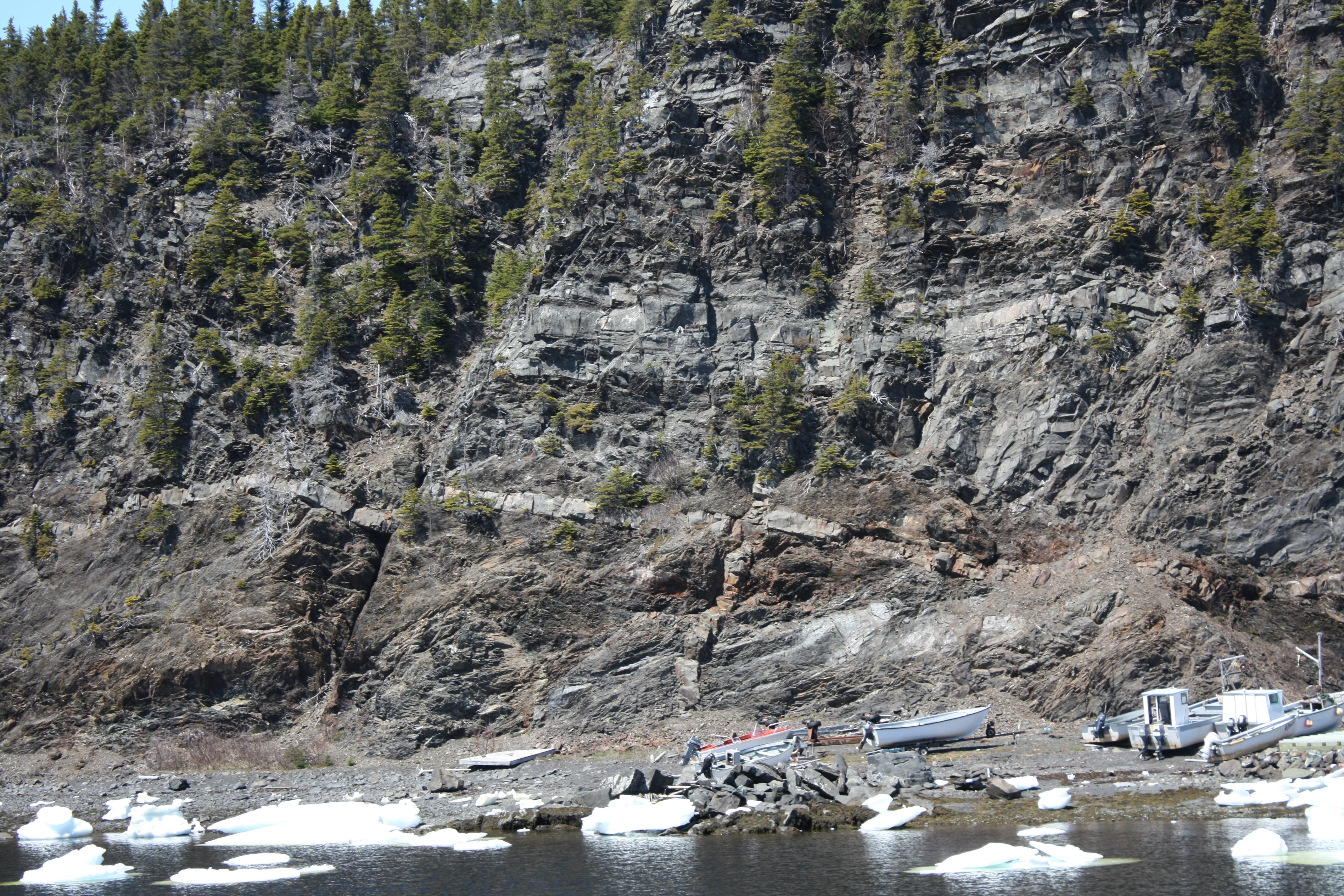

Supplement: Supplementary file 5 — Higher resolution version of field photographs (.jpg) contained in the Google Earth map file (.kmz). [file mmc6.zip › IMG_3472.JPG]

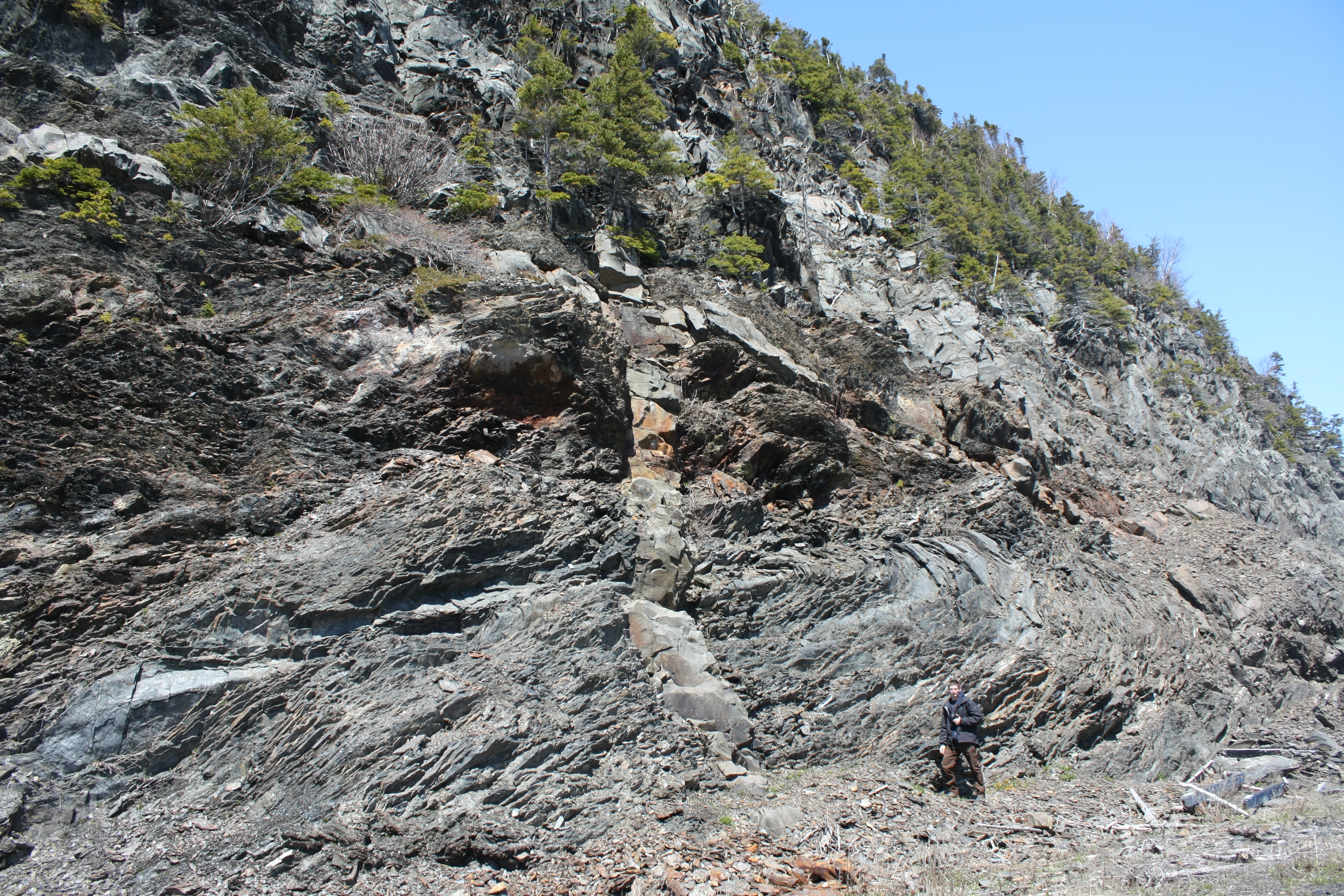

Supplement: Supplementary file 5 — Higher resolution version of field photographs (.jpg) contained in the Google Earth map file (.kmz). [file mmc6.zip › IMG_3479.JPG]

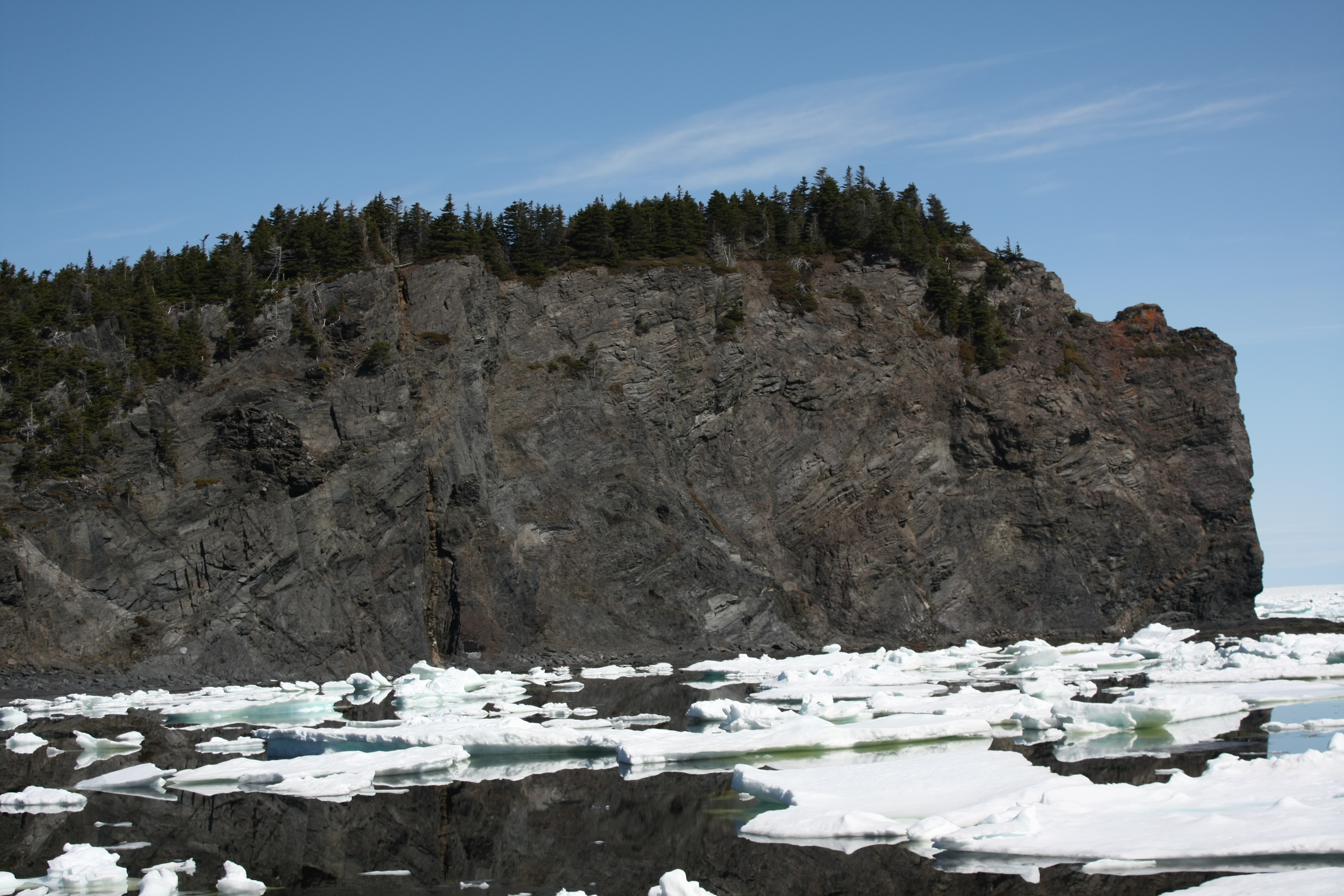

Supplement: Supplementary file 5 — Higher resolution version of field photographs (.jpg) contained in the Google Earth map file (.kmz). [file mmc6.zip › IMG_3516.JPG]

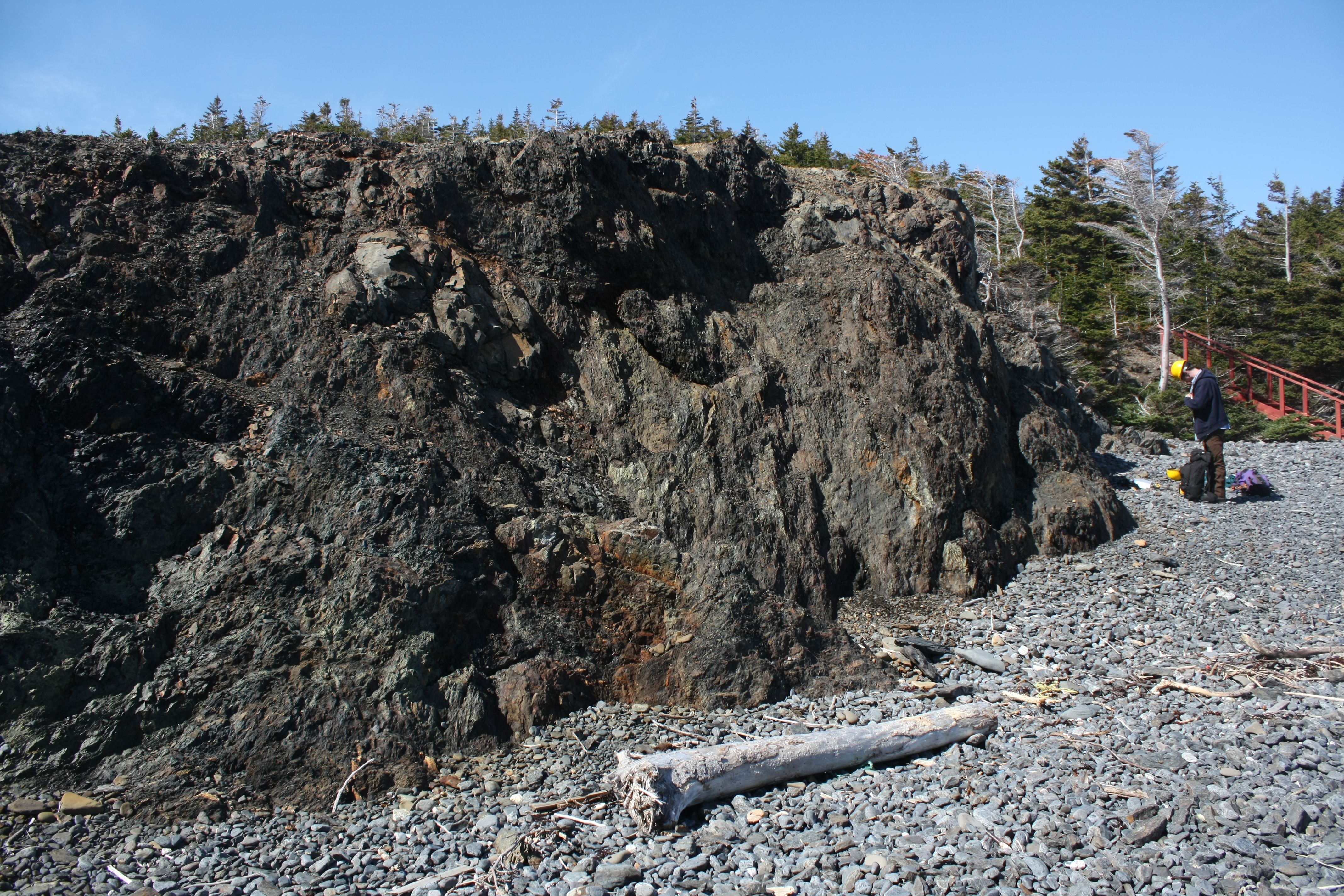

Supplement: Supplementary file 5 — Higher resolution version of field photographs (.jpg) contained in the Google Earth map file (.kmz). [file mmc6.zip › IMG_3521.JPG]

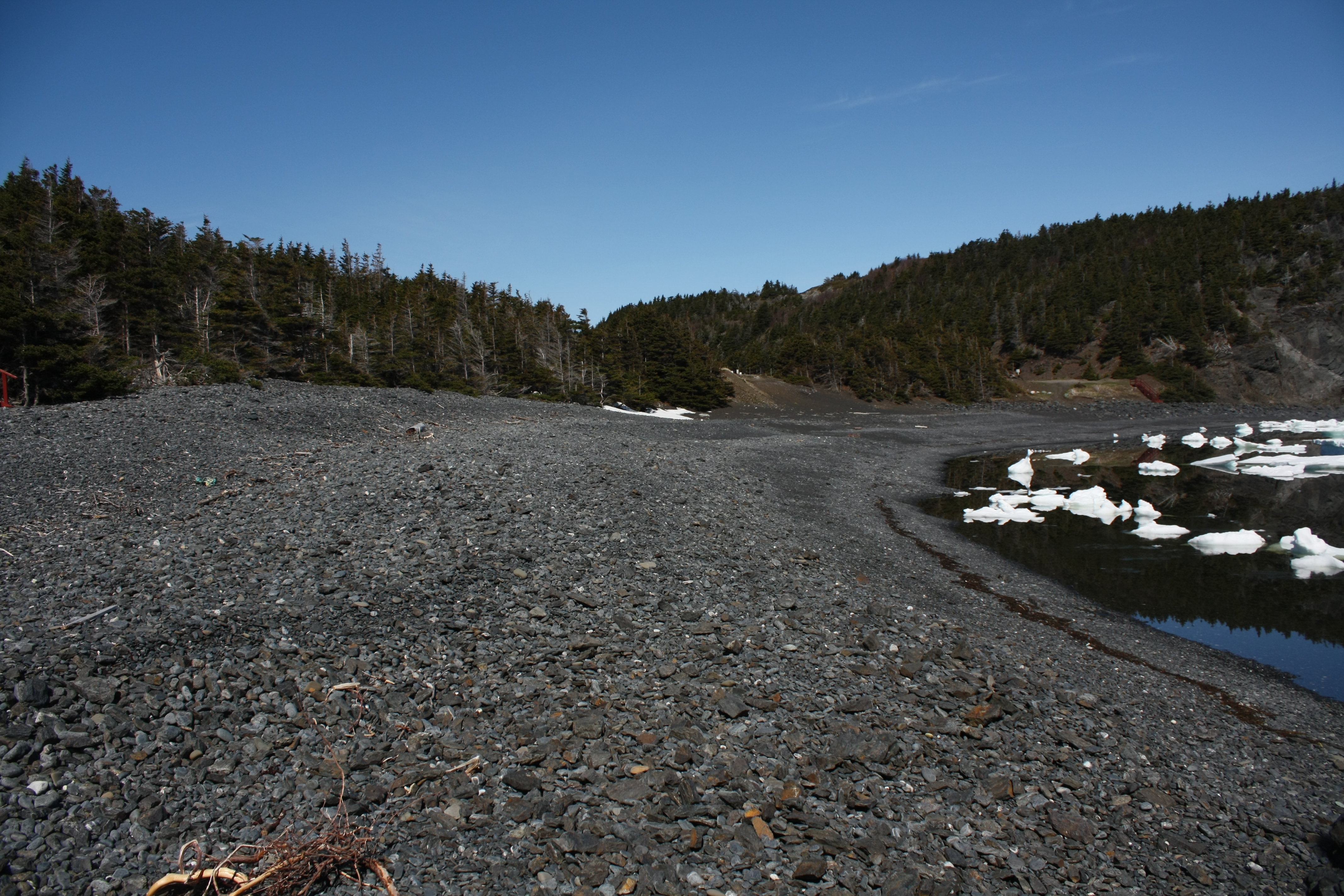

Supplement: Supplementary file 5 — Higher resolution version of field photographs (.jpg) contained in the Google Earth map file (.kmz). [file mmc6.zip › IMG_3531.JPG]

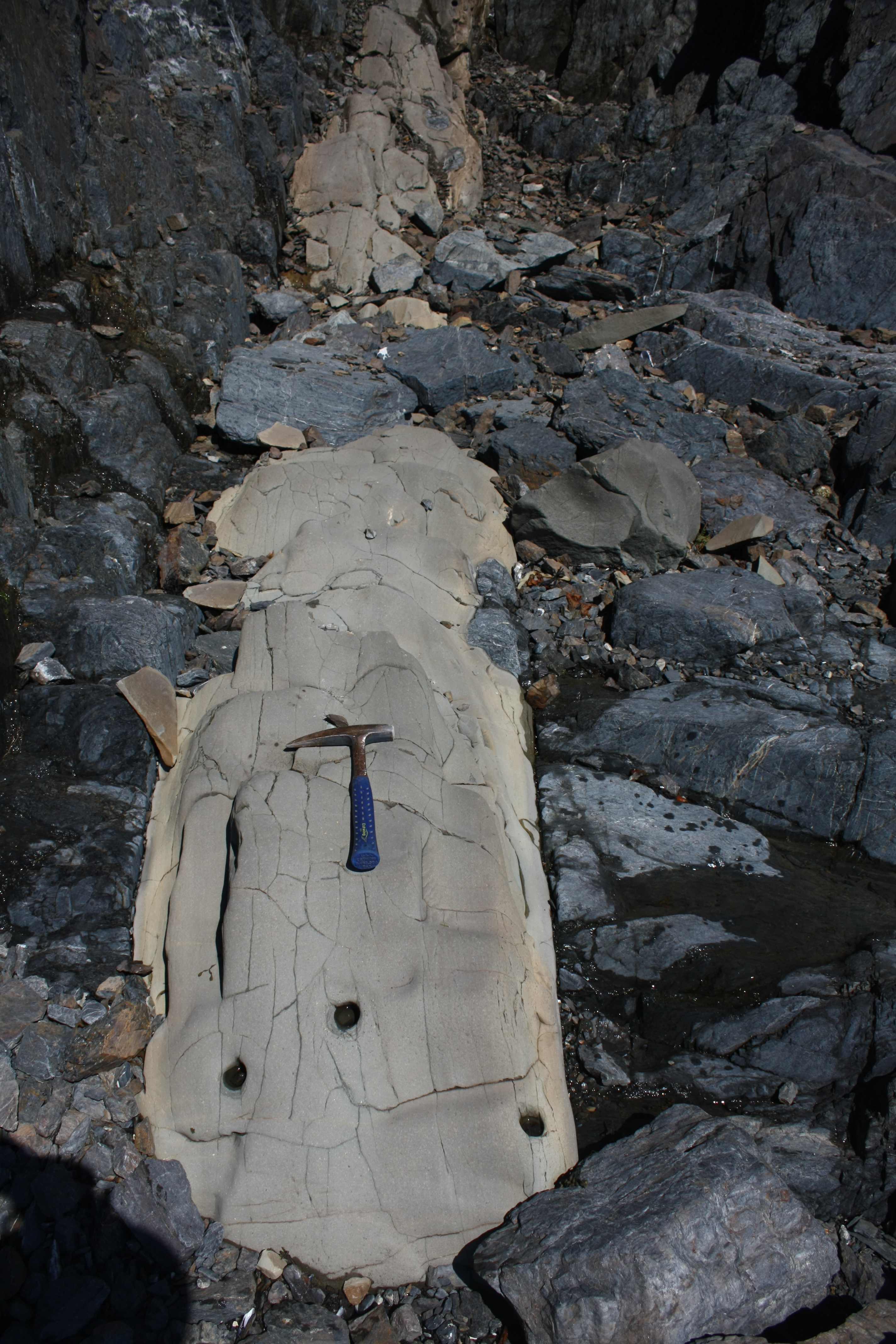

Supplement: Supplementary file 5 — Higher resolution version of field photographs (.jpg) contained in the Google Earth map file (.kmz). [file mmc6.zip › IMG_3543.JPG]

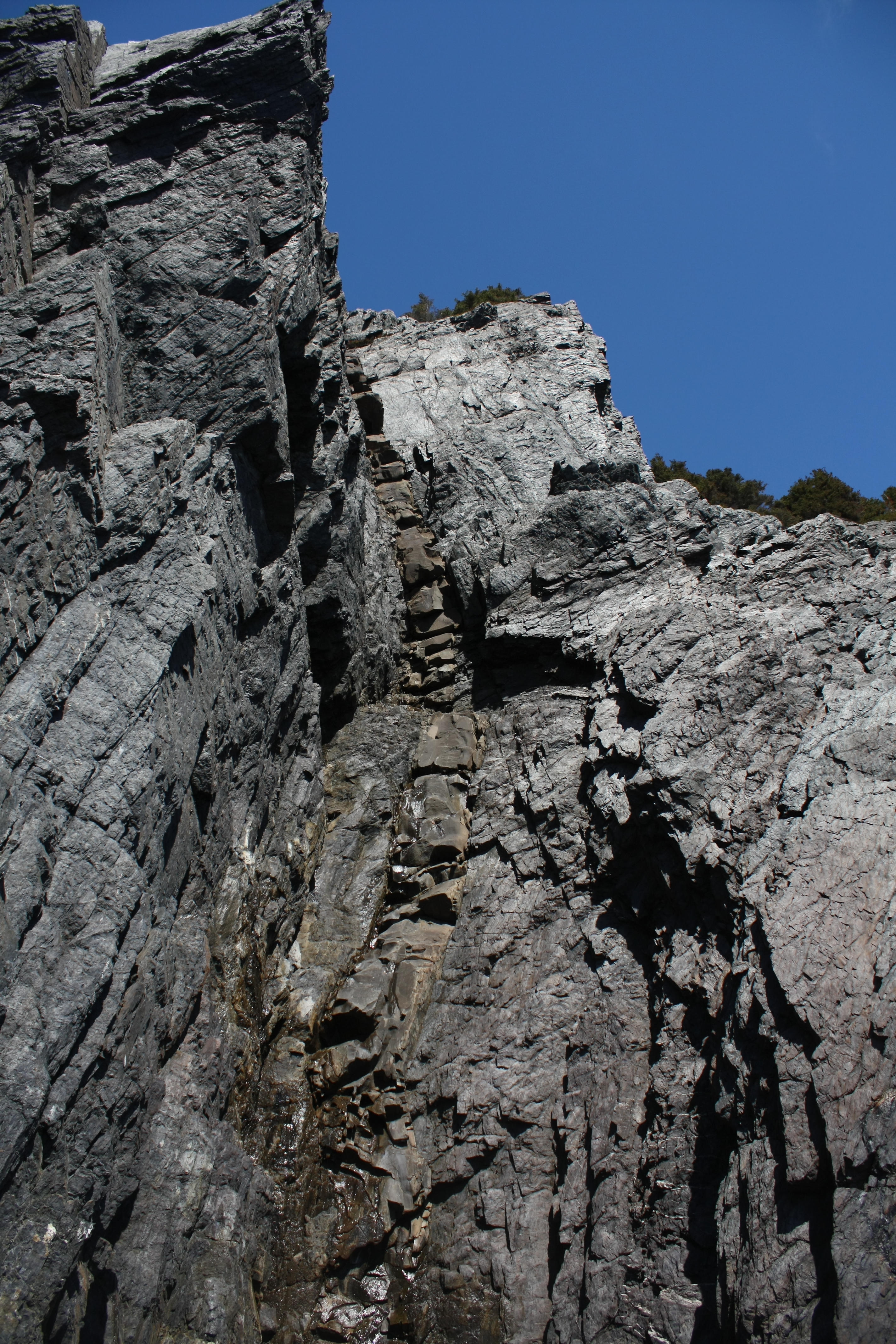

Supplement: Supplementary file 5 — Higher resolution version of field photographs (.jpg) contained in the Google Earth map file (.kmz). [file mmc6.zip › IMG_3546.JPG]

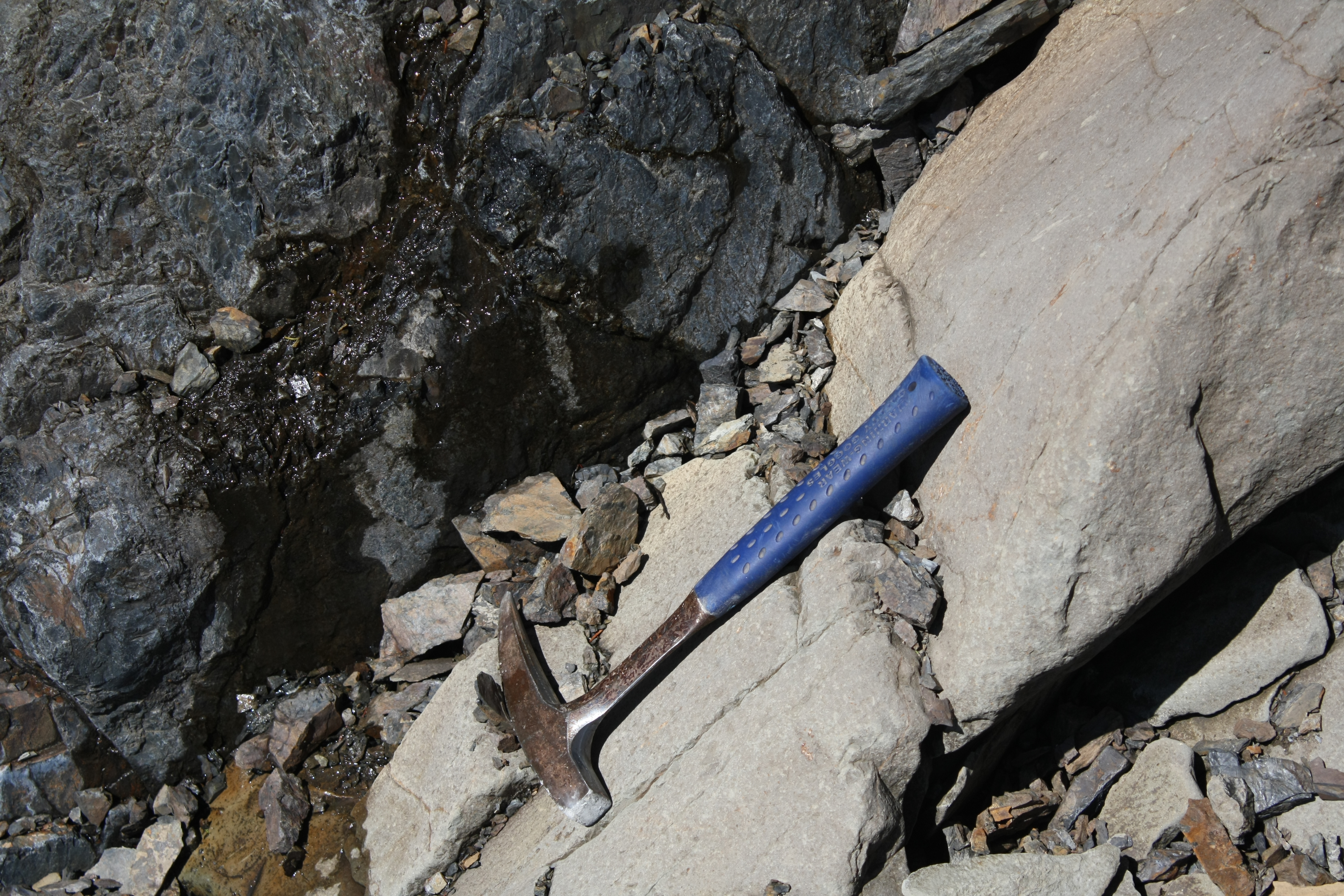

Supplement: Supplementary file 5 — Higher resolution version of field photographs (.jpg) contained in the Google Earth map file (.kmz). [file mmc6.zip › IMG_3548.JPG]

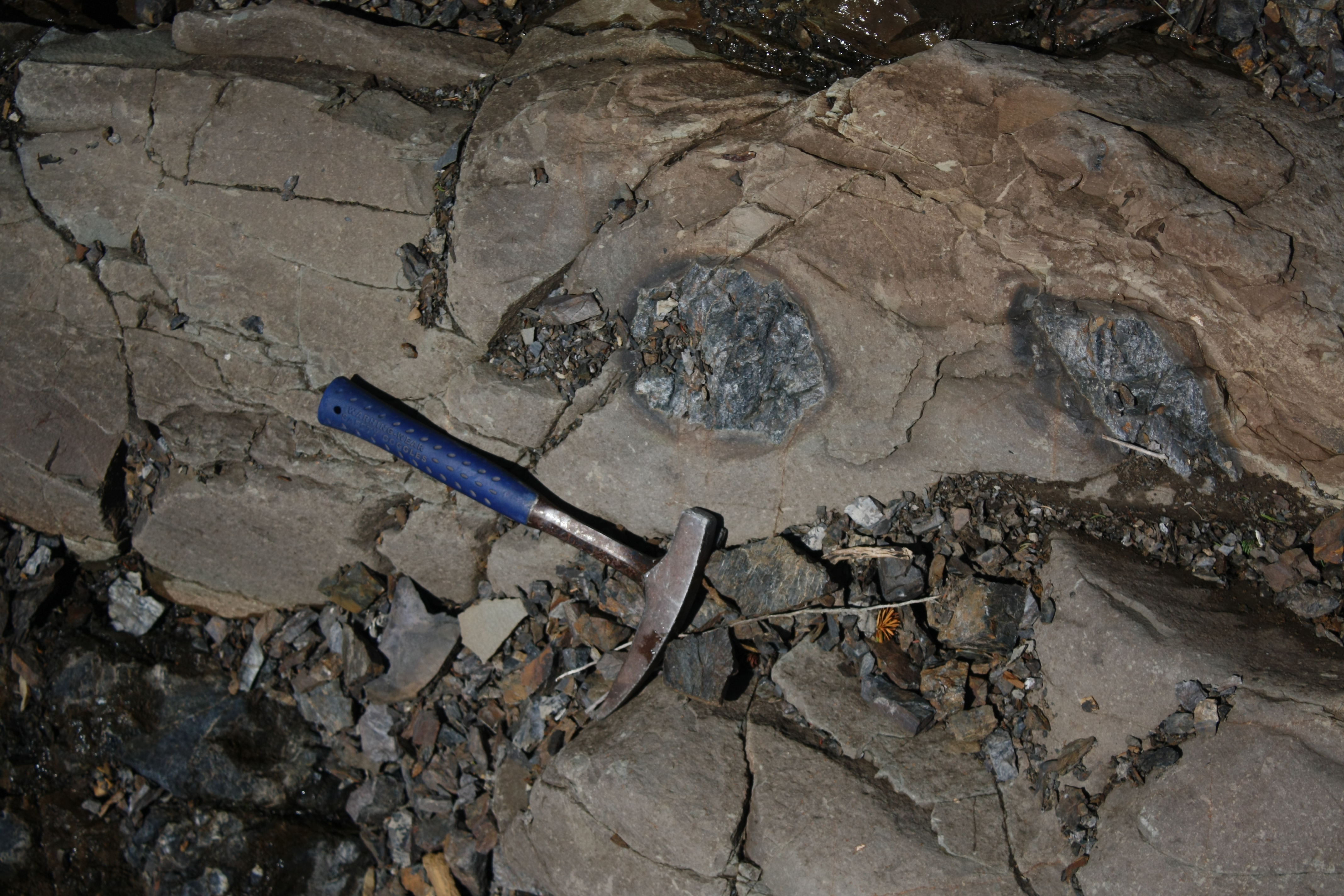

Supplement: Supplementary file 5 — Higher resolution version of field photographs (.jpg) contained in the Google Earth map file (.kmz). [file mmc6.zip › IMG_3550.JPG]

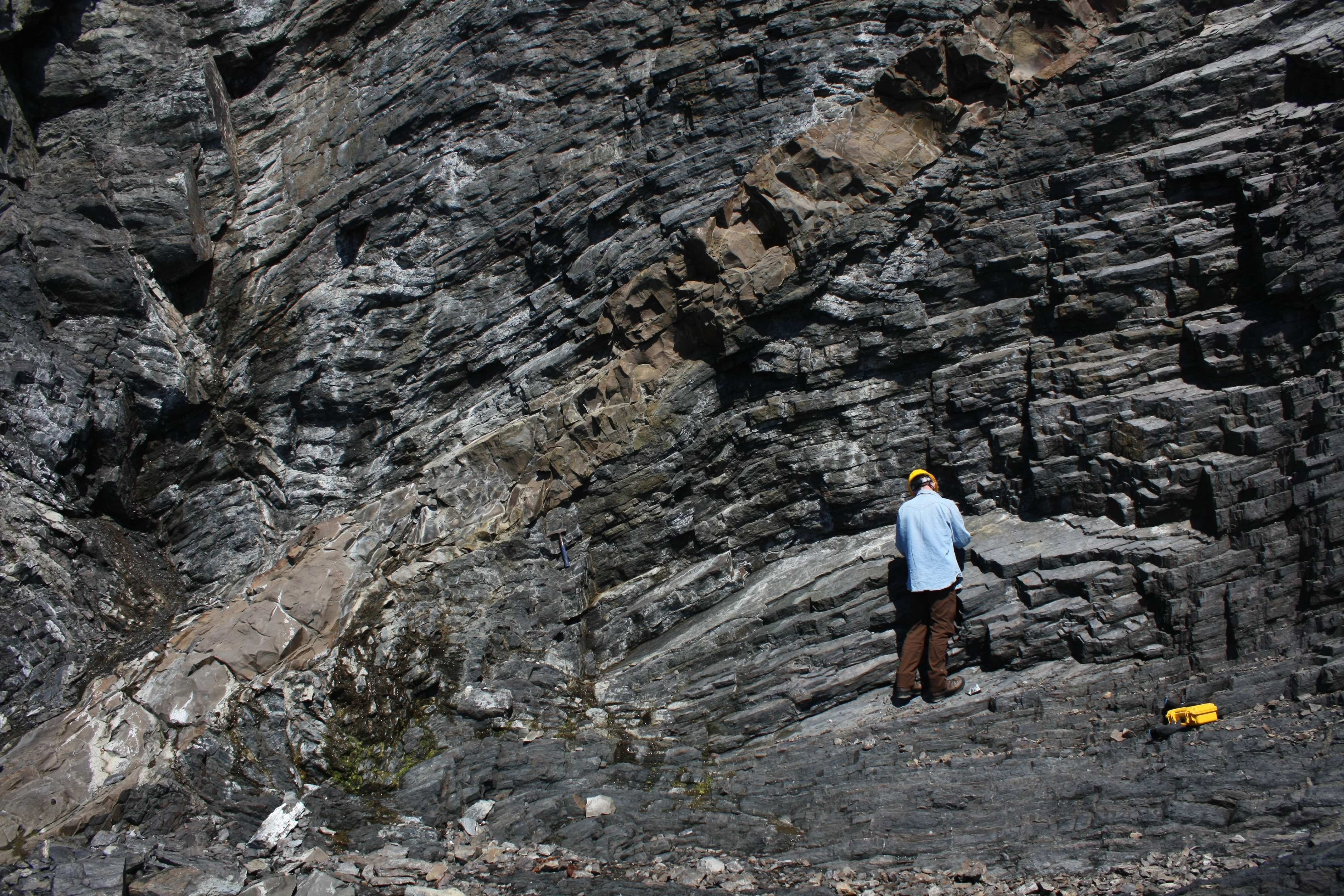

Supplement: Supplementary file 5 — Higher resolution version of field photographs (.jpg) contained in the Google Earth map file (.kmz). [file mmc6.zip › IMG_3556.JPG]

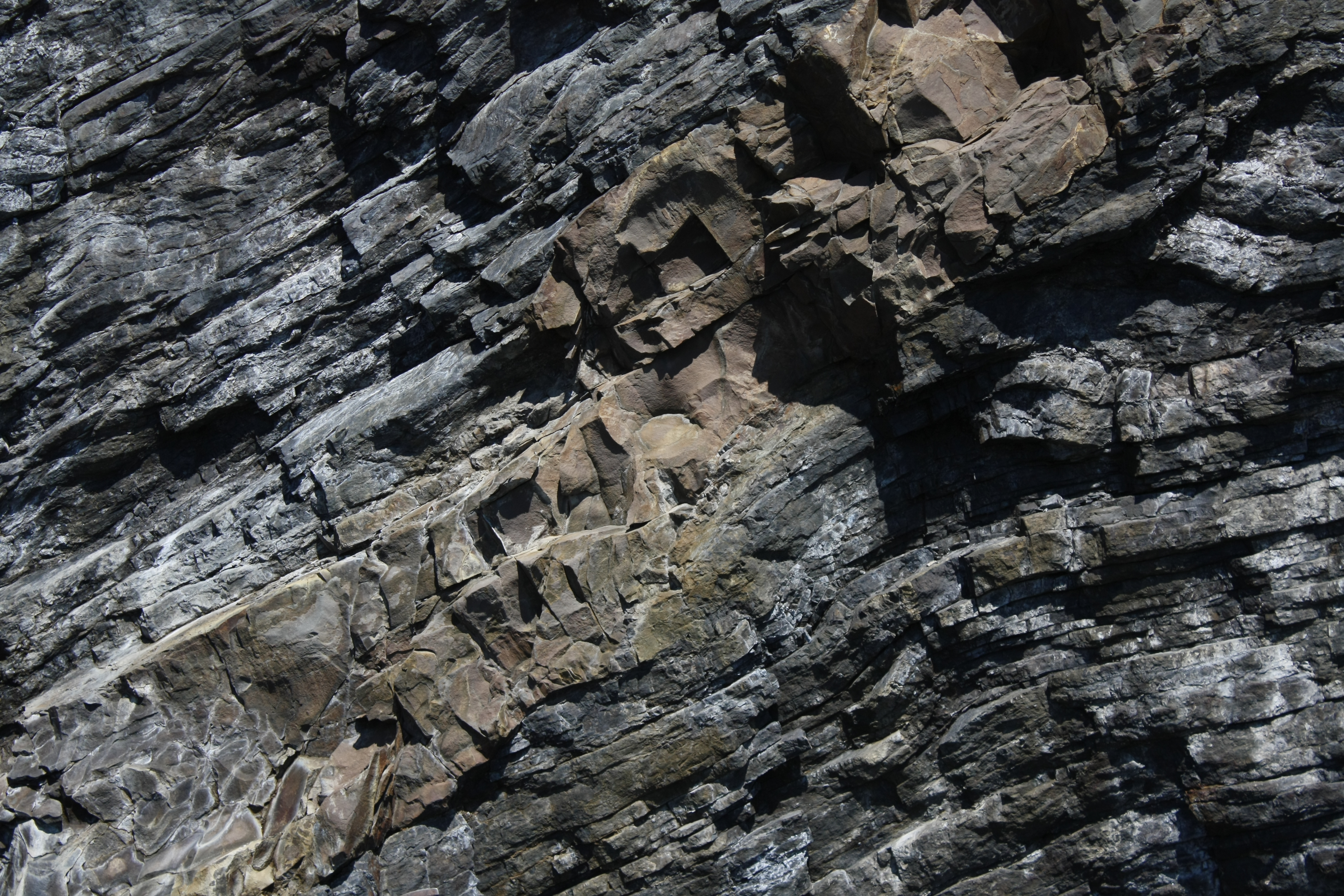

Supplement: Supplementary file 5 — Higher resolution version of field photographs (.jpg) contained in the Google Earth map file (.kmz). [file mmc6.zip › IMG_3557.JPG]

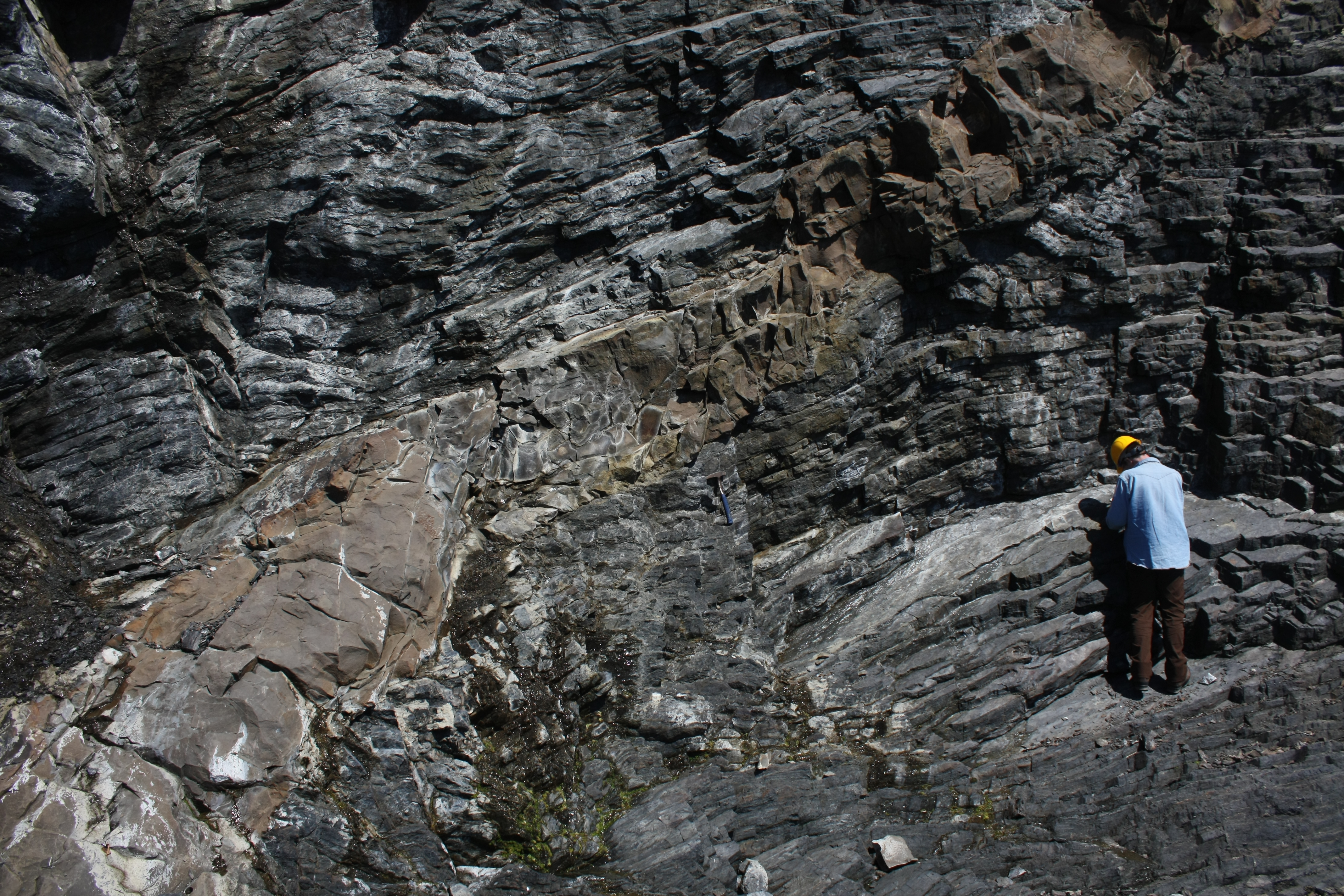

Supplement: Supplementary file 5 — Higher resolution version of field photographs (.jpg) contained in the Google Earth map file (.kmz). [file mmc6.zip › IMG_3558.JPG]

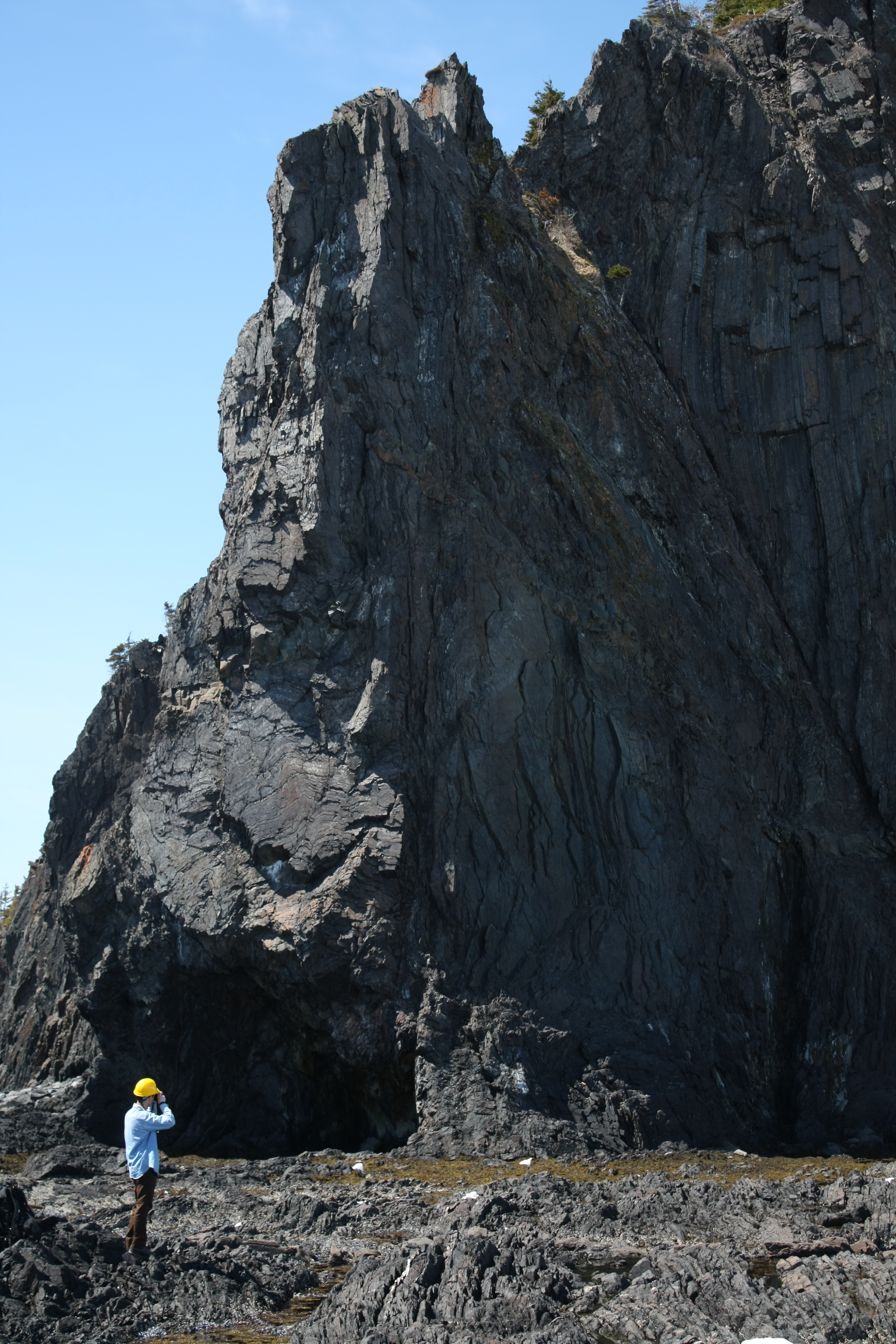

Supplement: Supplementary file 5 — Higher resolution version of field photographs (.jpg) contained in the Google Earth map file (.kmz). [file mmc6.zip › IMG_3574.JPG]

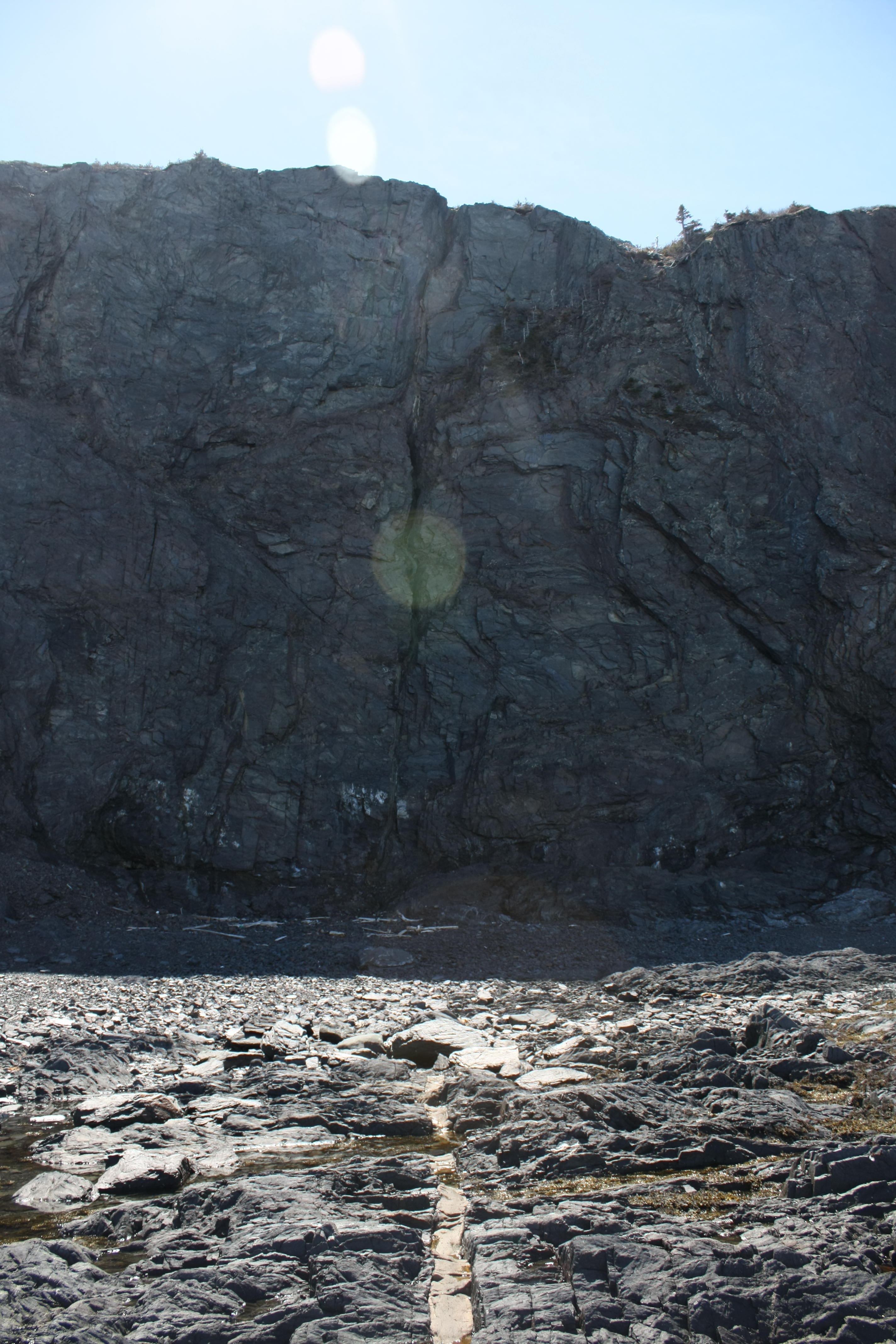

Supplement: Supplementary file 5 — Higher resolution version of field photographs (.jpg) contained in the Google Earth map file (.kmz). [file mmc6.zip › IMG_3607.JPG]

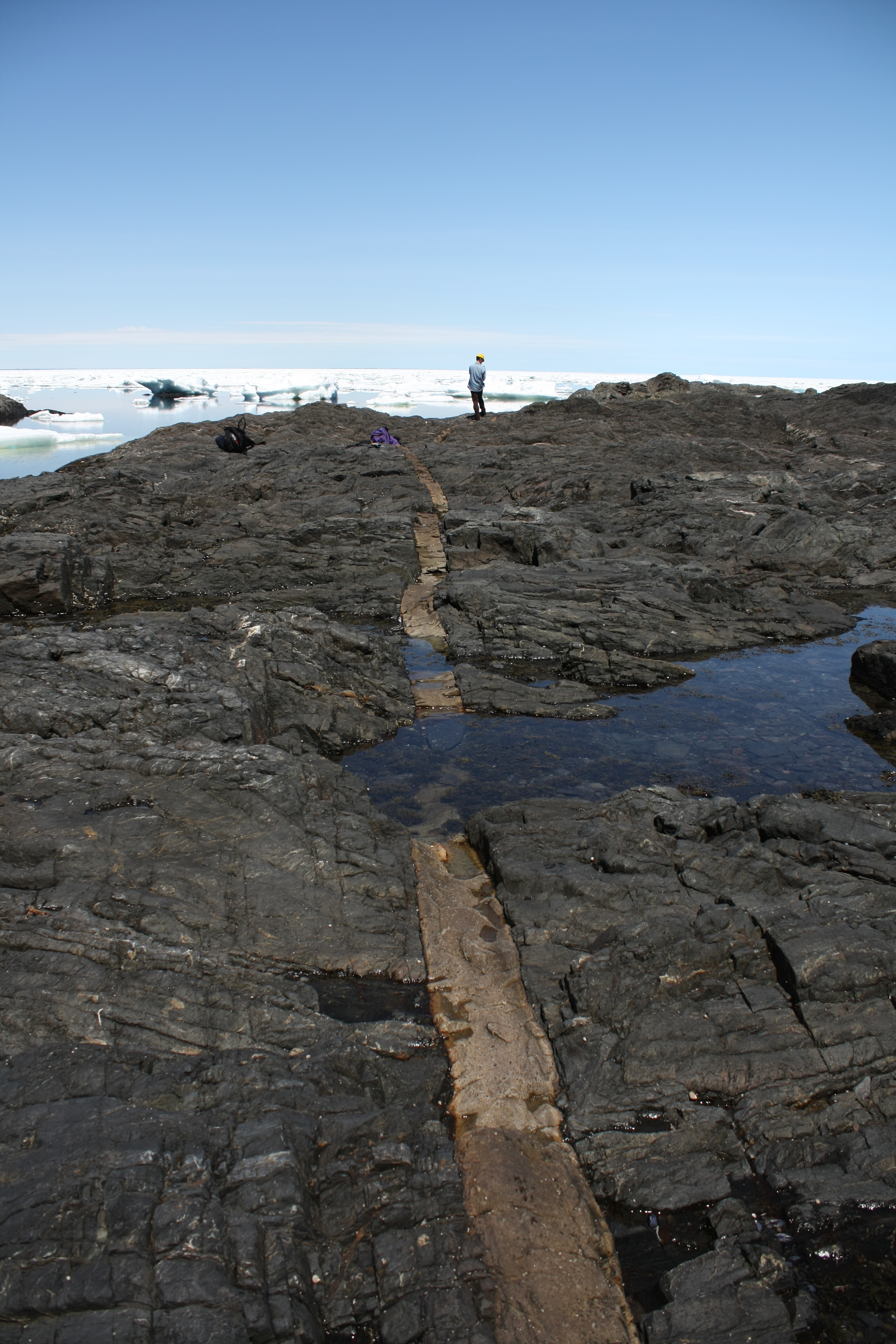

Supplement: Supplementary file 5 — Higher resolution version of field photographs (.jpg) contained in the Google Earth map file (.kmz). [file mmc6.zip › IMG_3609.JPG]

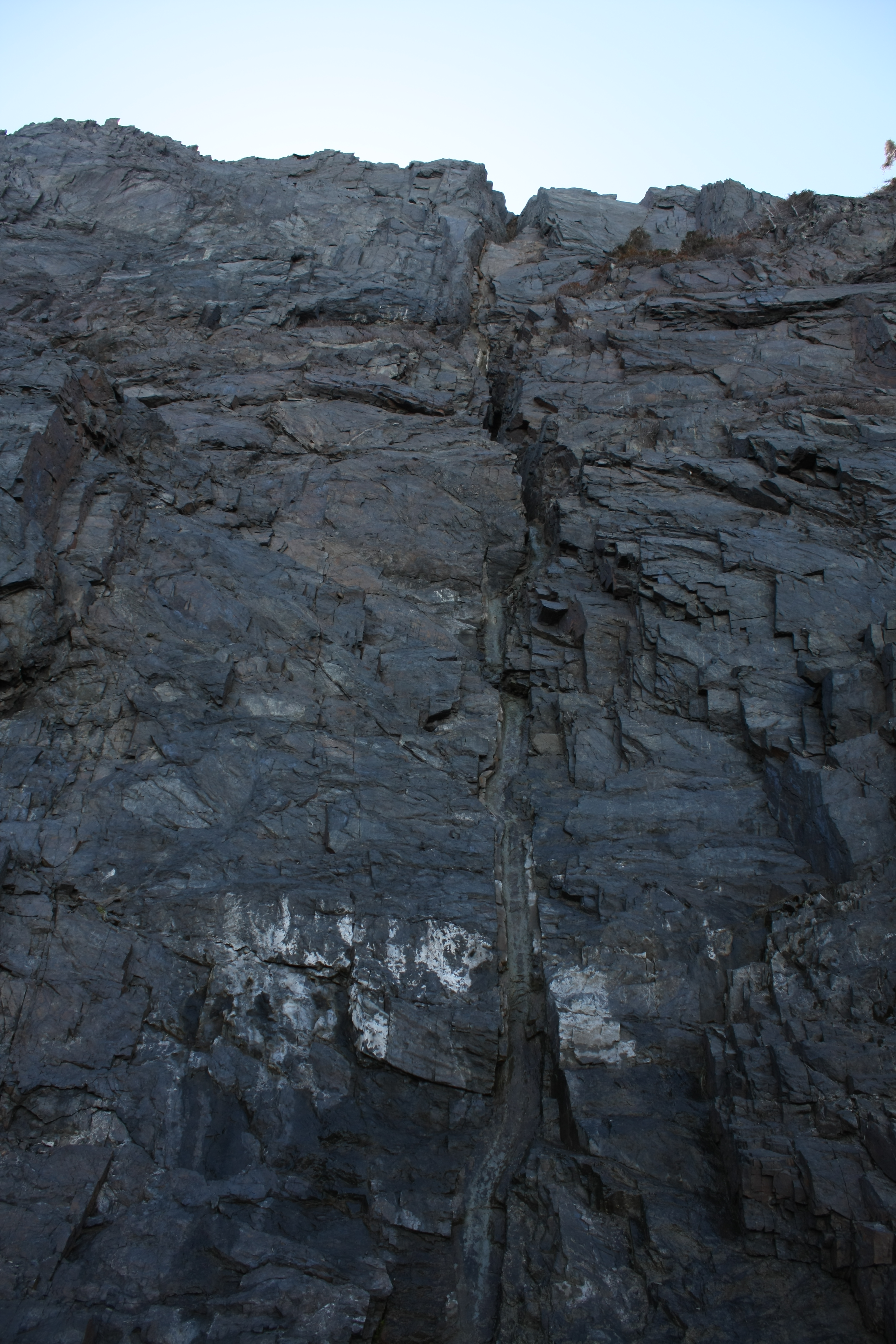

Supplement: Supplementary file 5 — Higher resolution version of field photographs (.jpg) contained in the Google Earth map file (.kmz). [file mmc6.zip › IMG_3612.JPG]

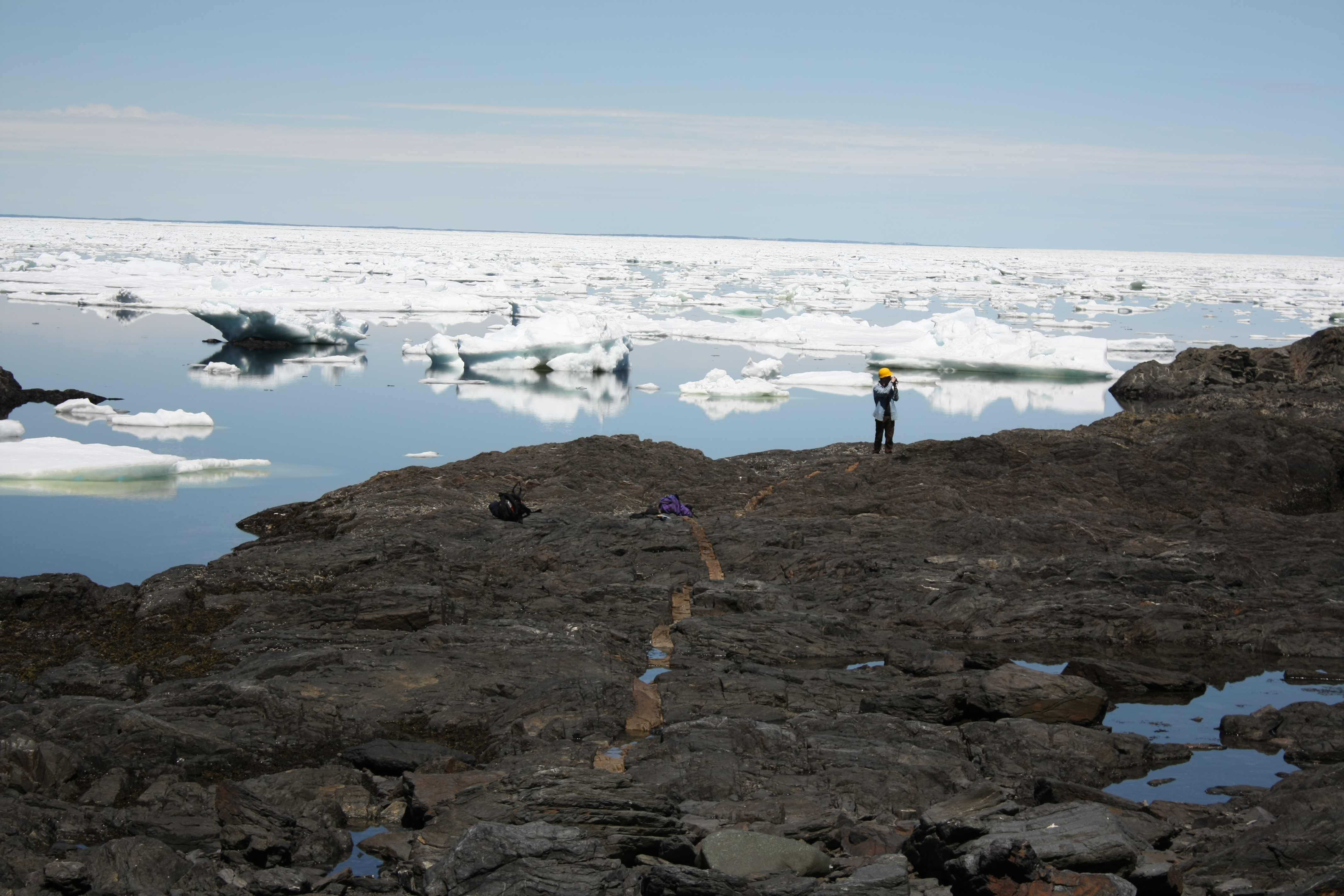

Supplement: Supplementary file 5 — Higher resolution version of field photographs (.jpg) contained in the Google Earth map file (.kmz). [file mmc6.zip › IMG_3617.JPG]

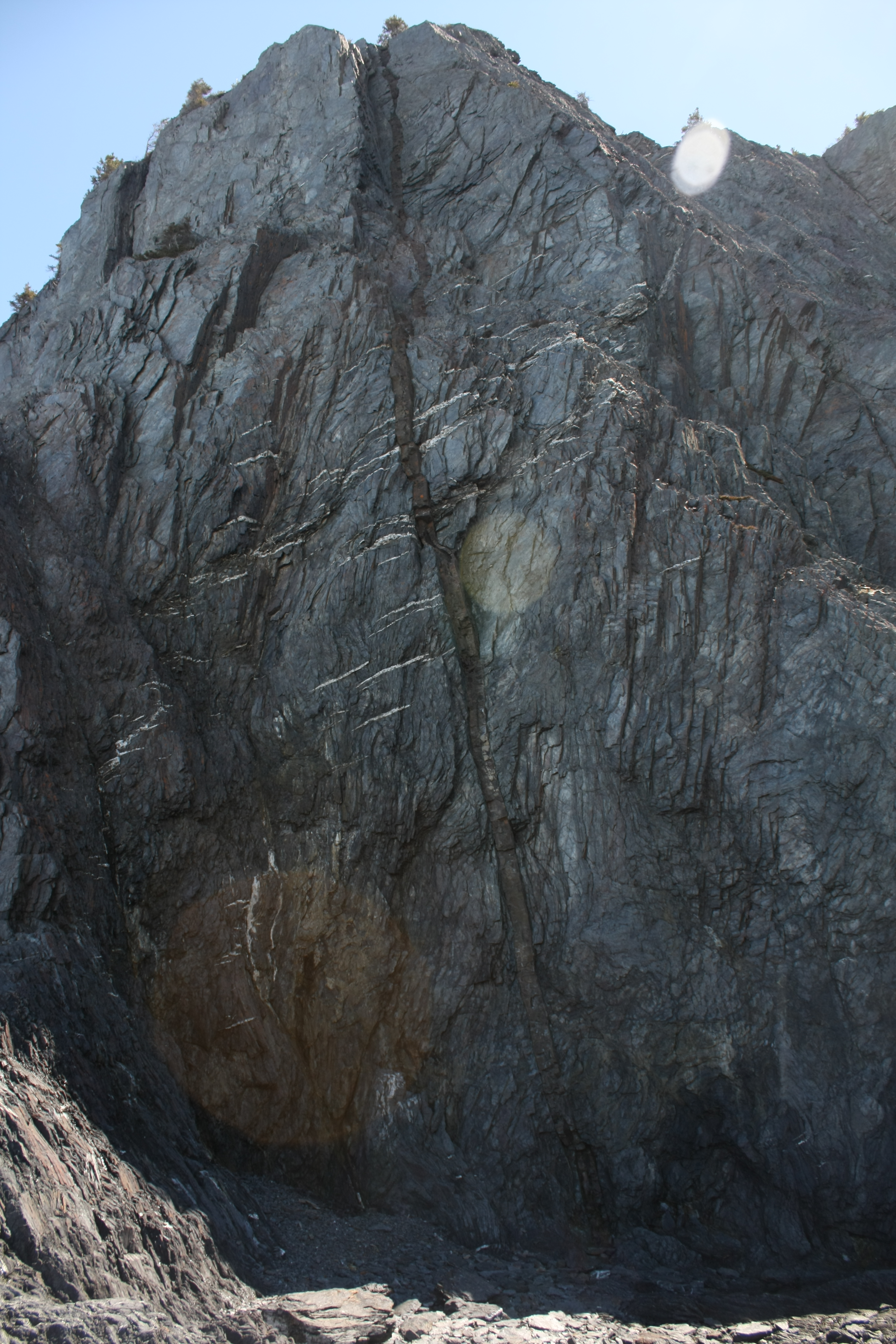

Supplement: Supplementary file 5 — Higher resolution version of field photographs (.jpg) contained in the Google Earth map file (.kmz). [file mmc6.zip › IMG_3621.JPG]

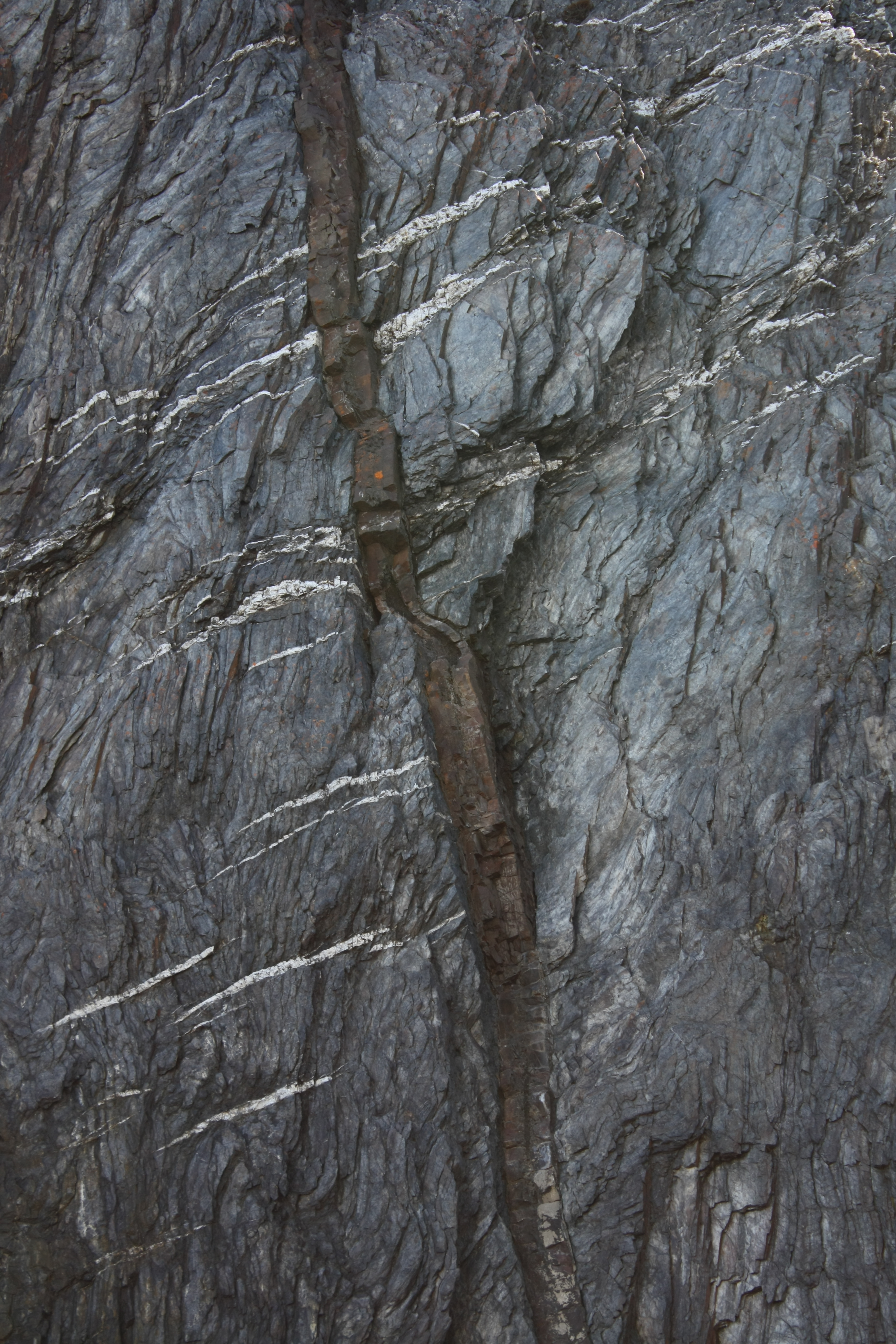

Supplement: Supplementary file 5 — Higher resolution version of field photographs (.jpg) contained in the Google Earth map file (.kmz). [file mmc6.zip › IMG_3622.JPG]

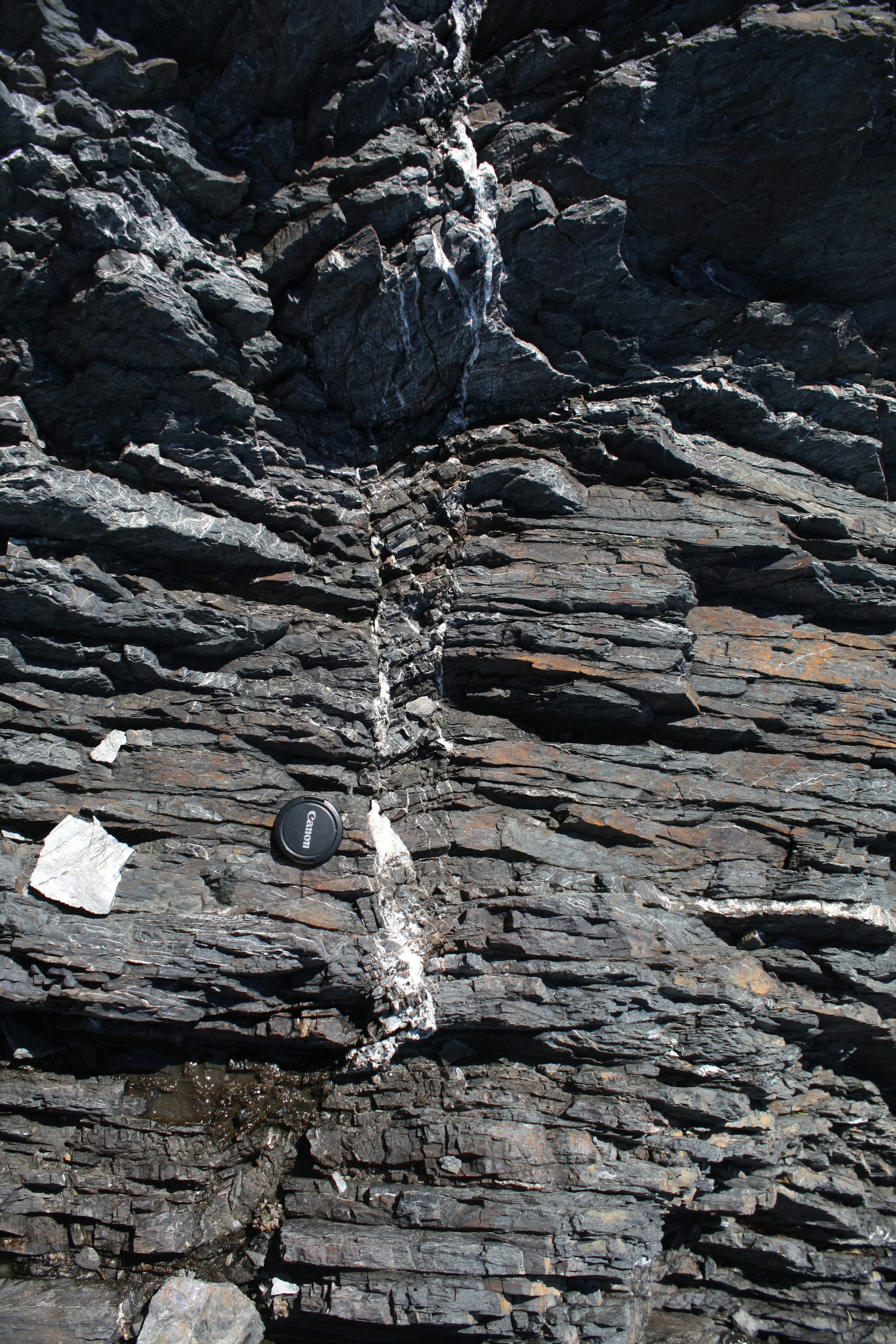

Supplement: Supplementary file 5 — Higher resolution version of field photographs (.jpg) contained in the Google Earth map file (.kmz). [file mmc6.zip › IMG_3640.JPG]

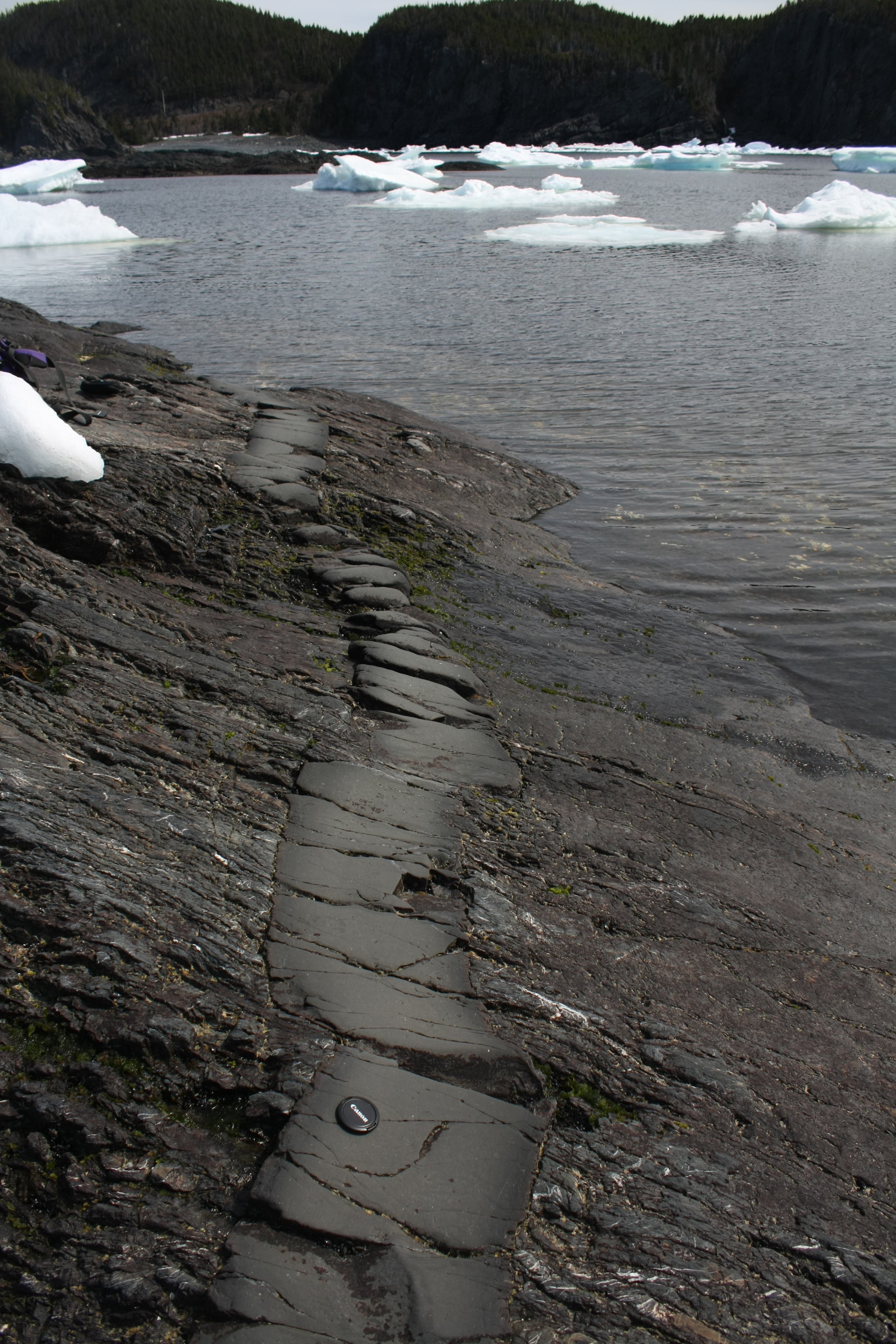

Supplement: Supplementary file 5 — Higher resolution version of field photographs (.jpg) contained in the Google Earth map file (.kmz). [file mmc6.zip › IMG_3672.JPG]

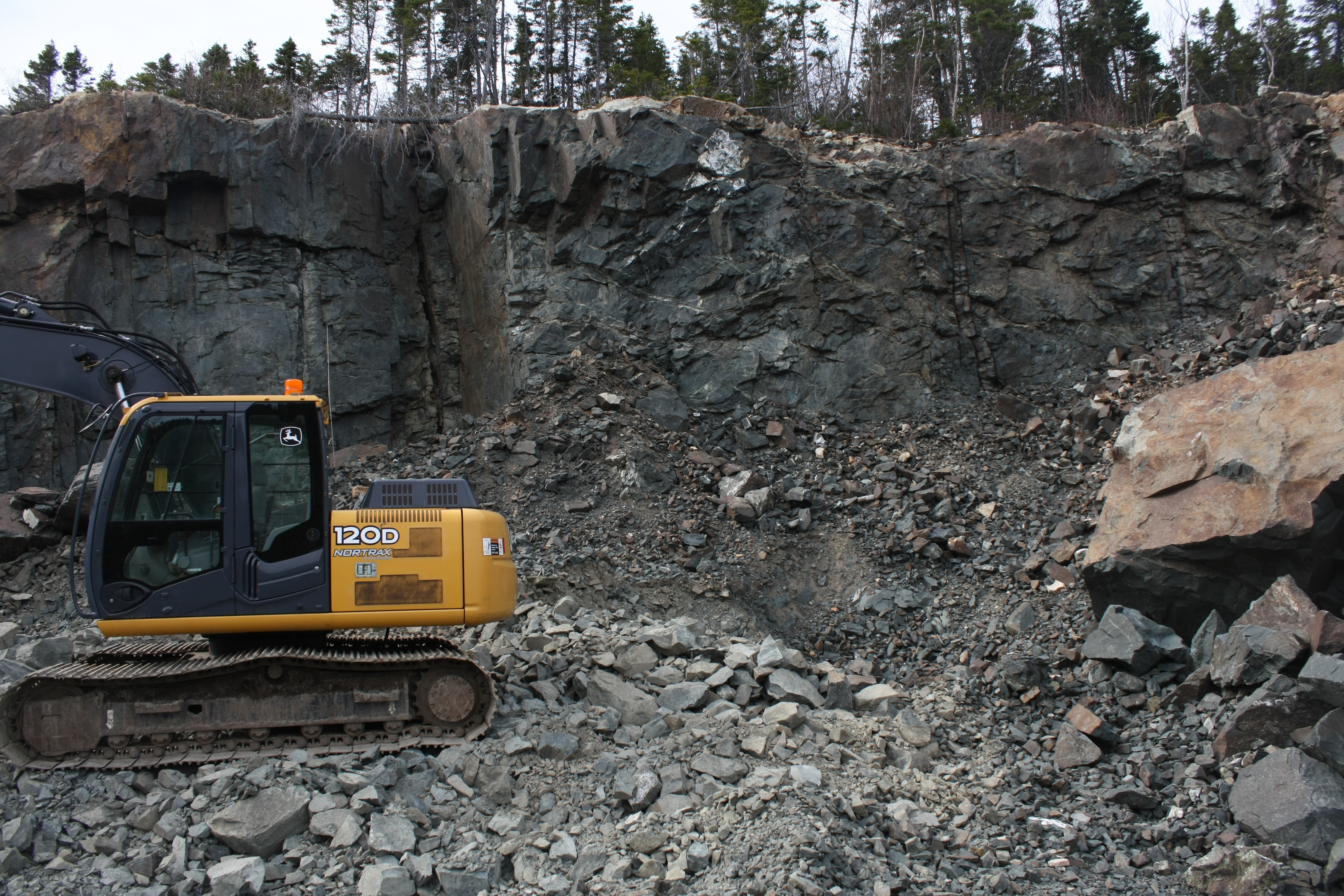

Supplement: Supplementary file 5 — Higher resolution version of field photographs (.jpg) contained in the Google Earth map file (.kmz). [file mmc6.zip › IMG_3681.JPG]

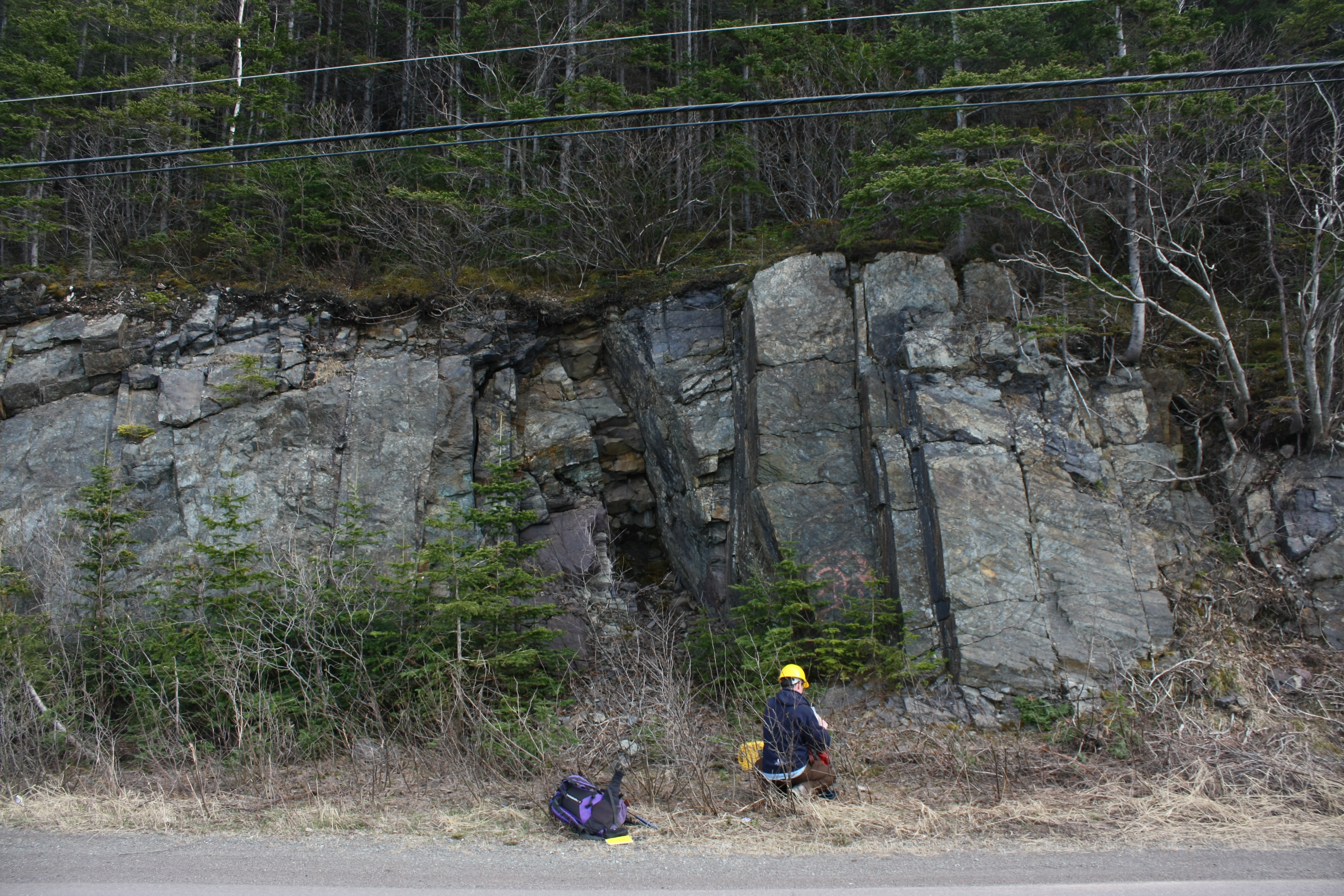

Supplement: Supplementary file 5 — Higher resolution version of field photographs (.jpg) contained in the Google Earth map file (.kmz). [file mmc6.zip › IMG_3693.JPG]

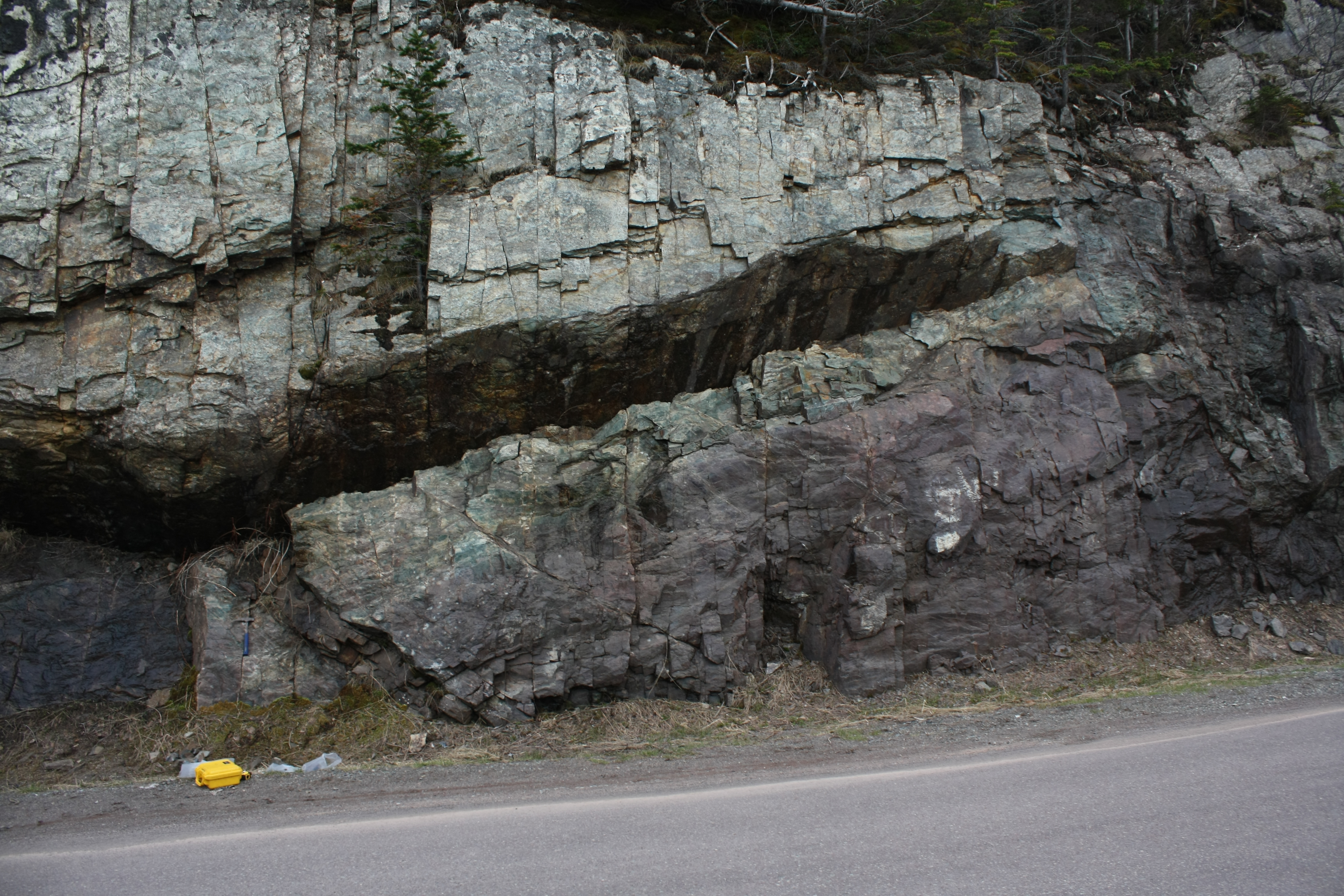

Supplement: Supplementary file 5 — Higher resolution version of field photographs (.jpg) contained in the Google Earth map file (.kmz). [file mmc6.zip › IMG_3718.JPG]

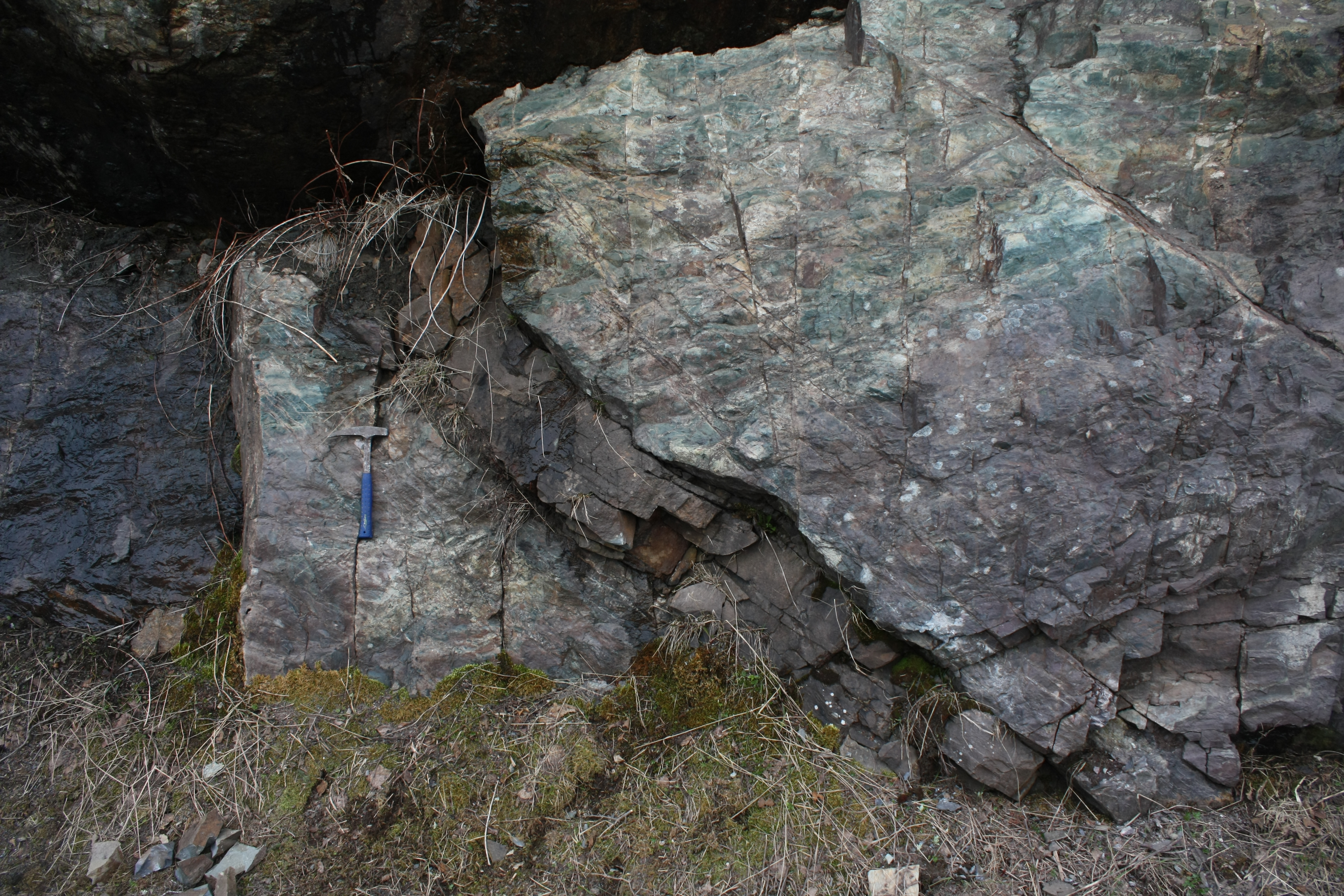

Supplement: Supplementary file 5 — Higher resolution version of field photographs (.jpg) contained in the Google Earth map file (.kmz). [file mmc6.zip › IMG_3722.JPG]

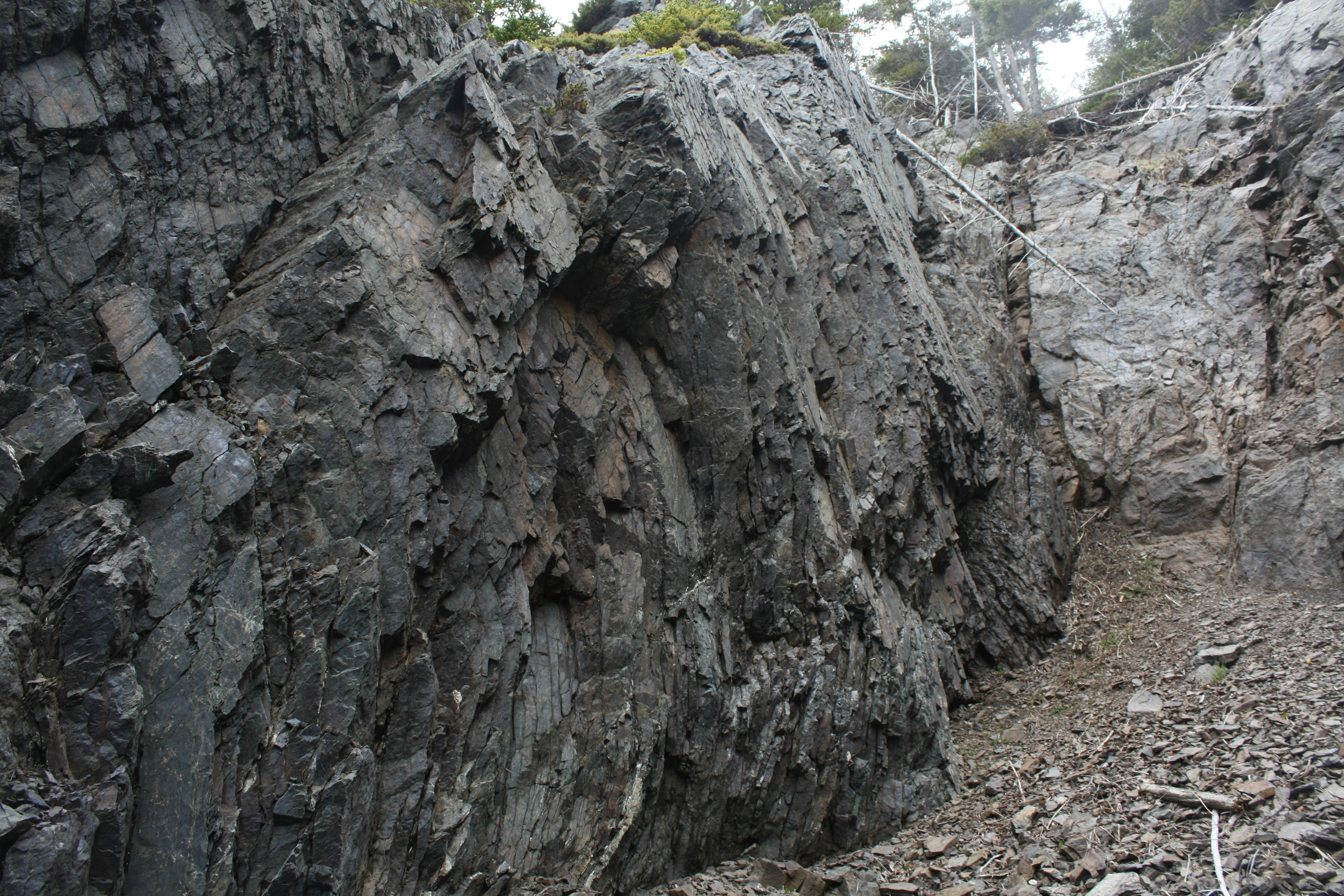

Supplement: Supplementary file 5 — Higher resolution version of field photographs (.jpg) contained in the Google Earth map file (.kmz). [file mmc6.zip › IMG_3741.JPG]

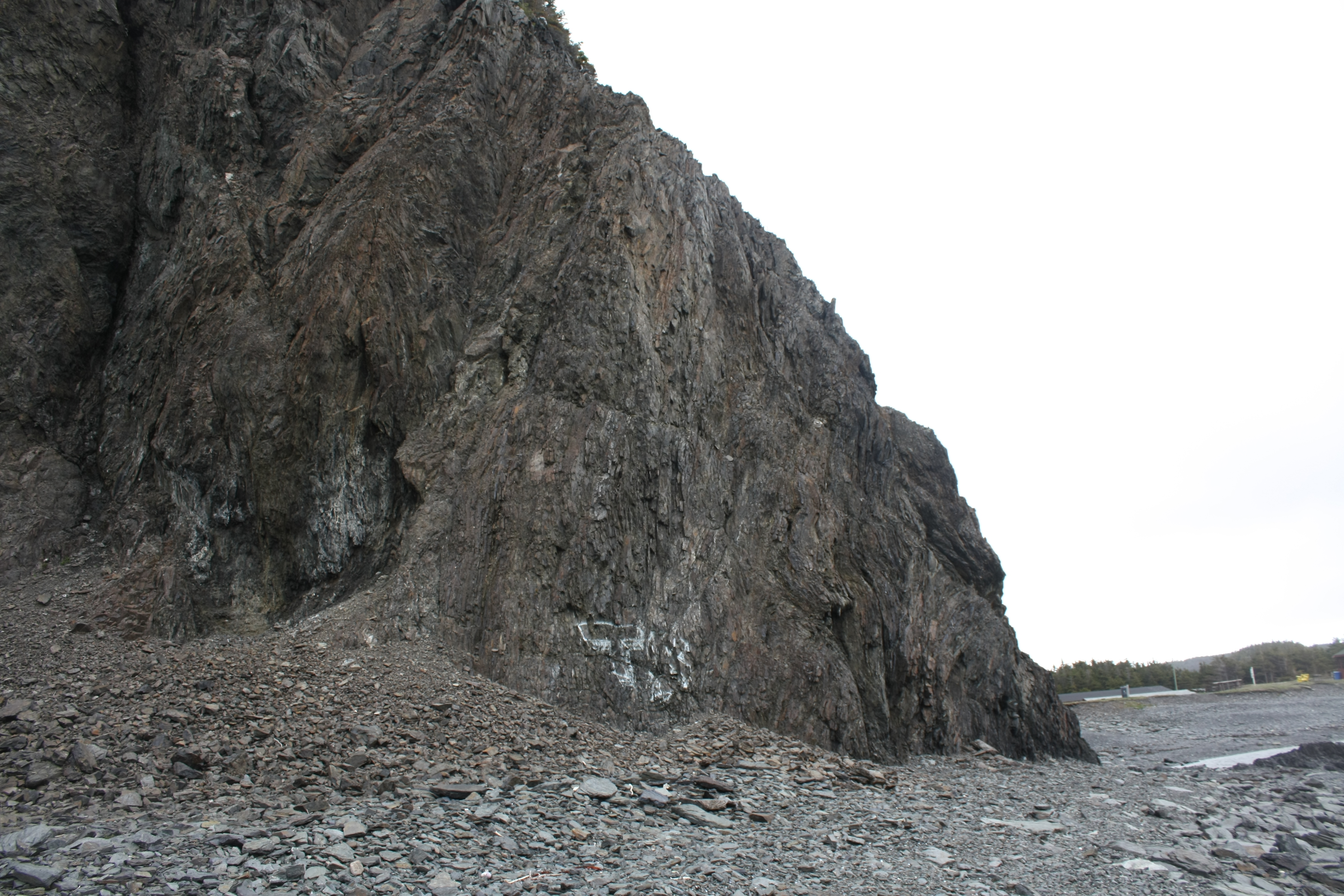

Supplement: Supplementary file 5 — Higher resolution version of field photographs (.jpg) contained in the Google Earth map file (.kmz). [file mmc6.zip › IMG_3776.JPG]

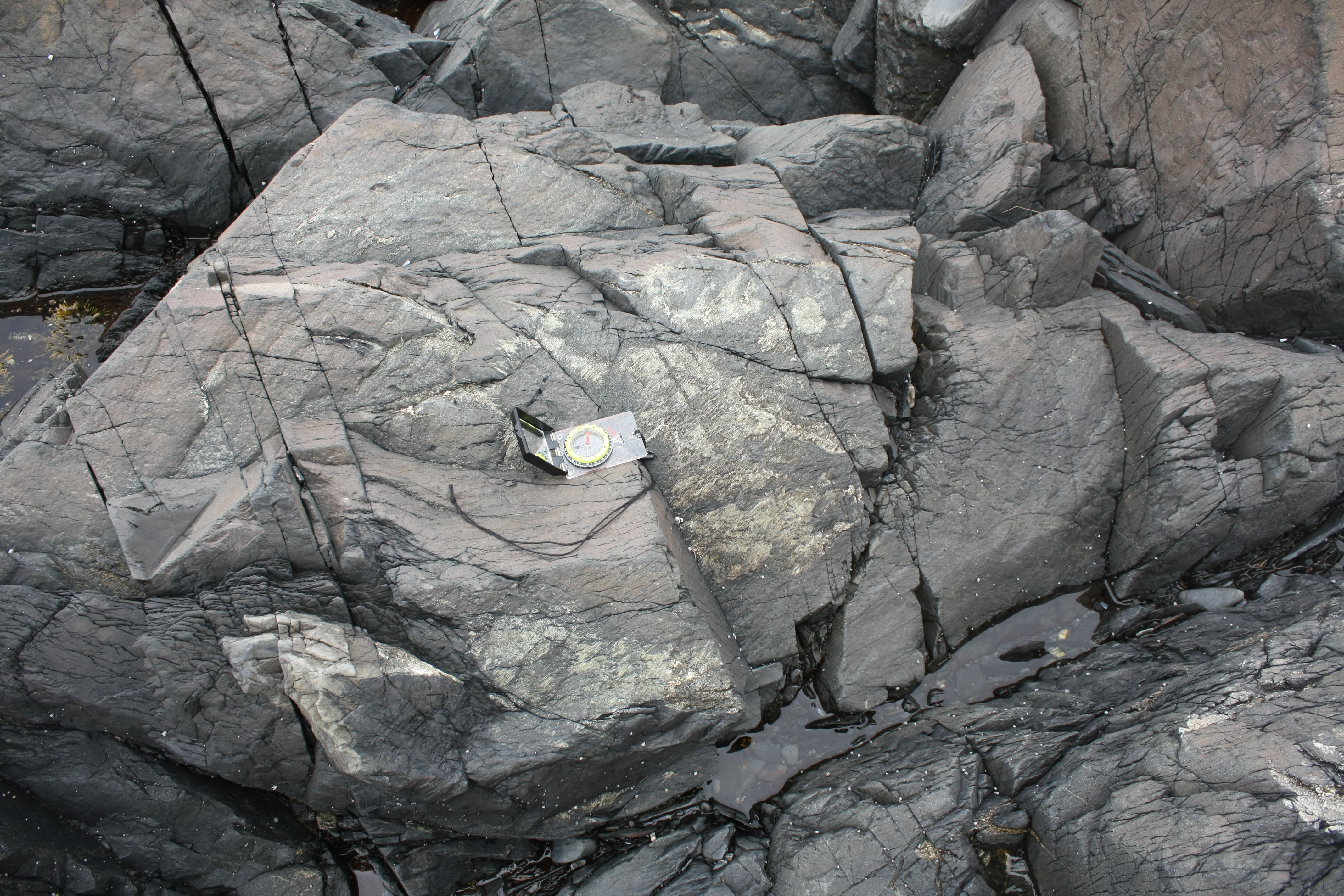

Supplement: Supplementary file 5 — Higher resolution version of field photographs (.jpg) contained in the Google Earth map file (.kmz). [file mmc6.zip › IMG_3791.JPG]

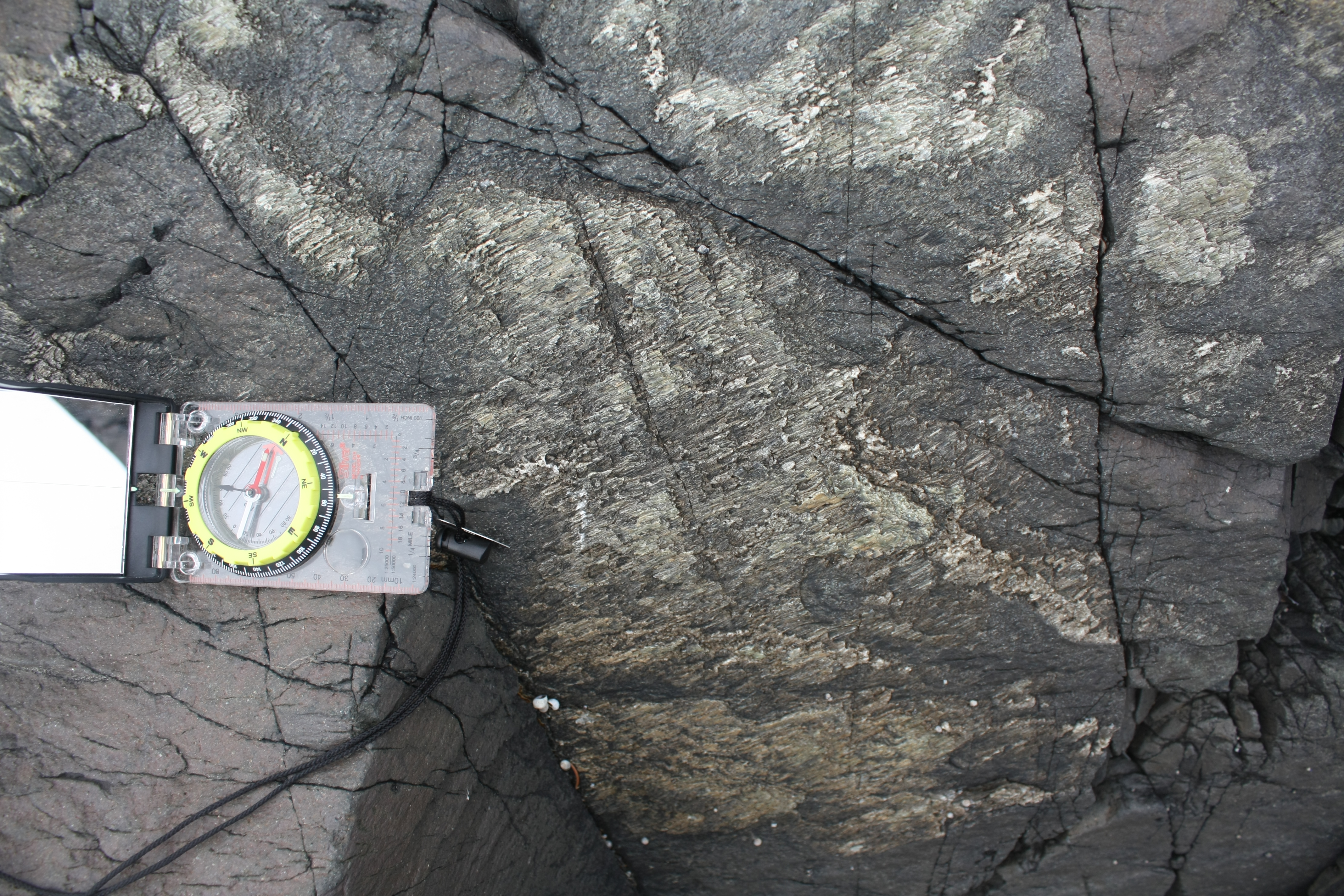

Supplement: Supplementary file 5 — Higher resolution version of field photographs (.jpg) contained in the Google Earth map file (.kmz). [file mmc6.zip › IMG_3796.JPG]

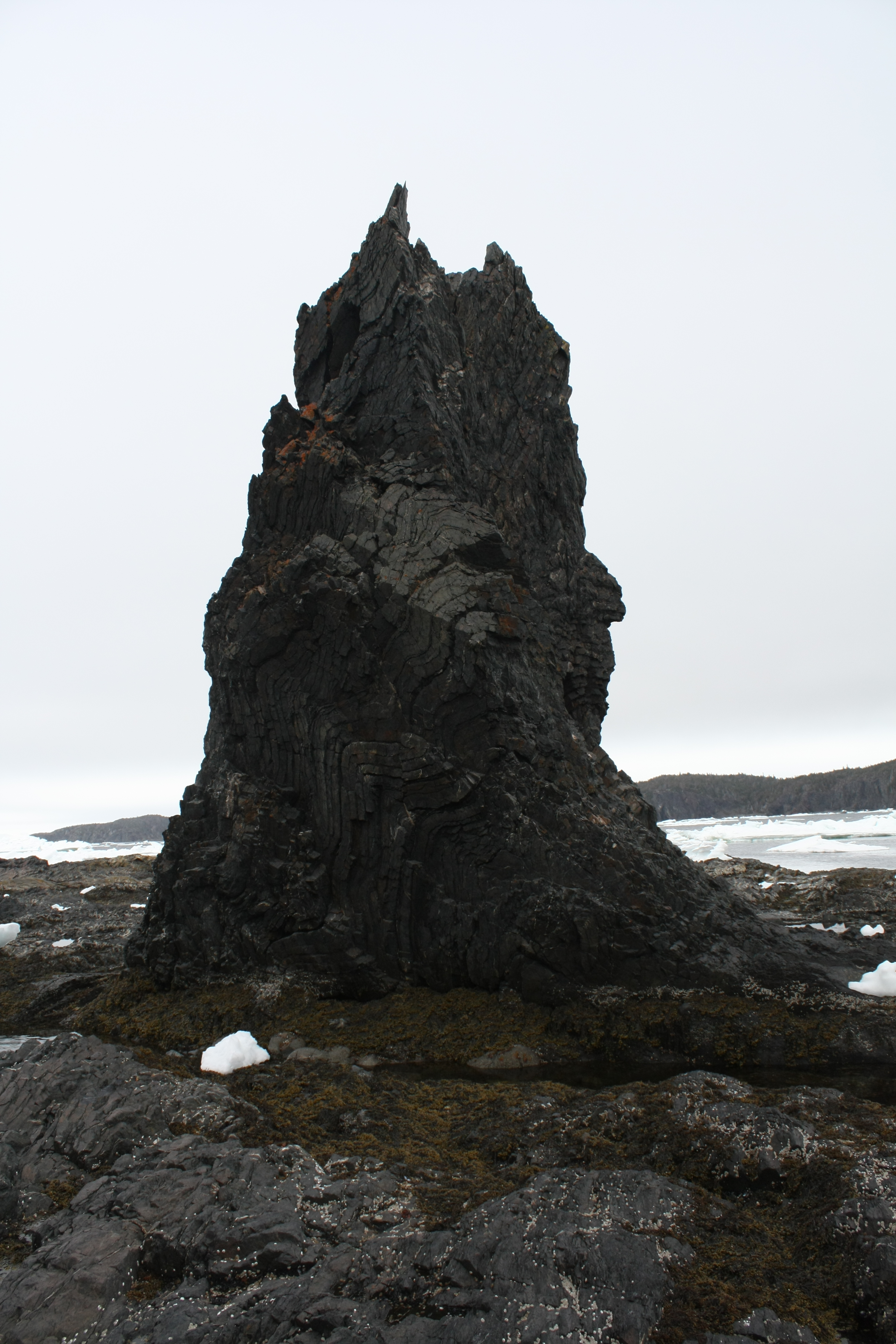

Supplement: Supplementary file 5 — Higher resolution version of field photographs (.jpg) contained in the Google Earth map file (.kmz). [file mmc6.zip › IMG_3828.JPG]

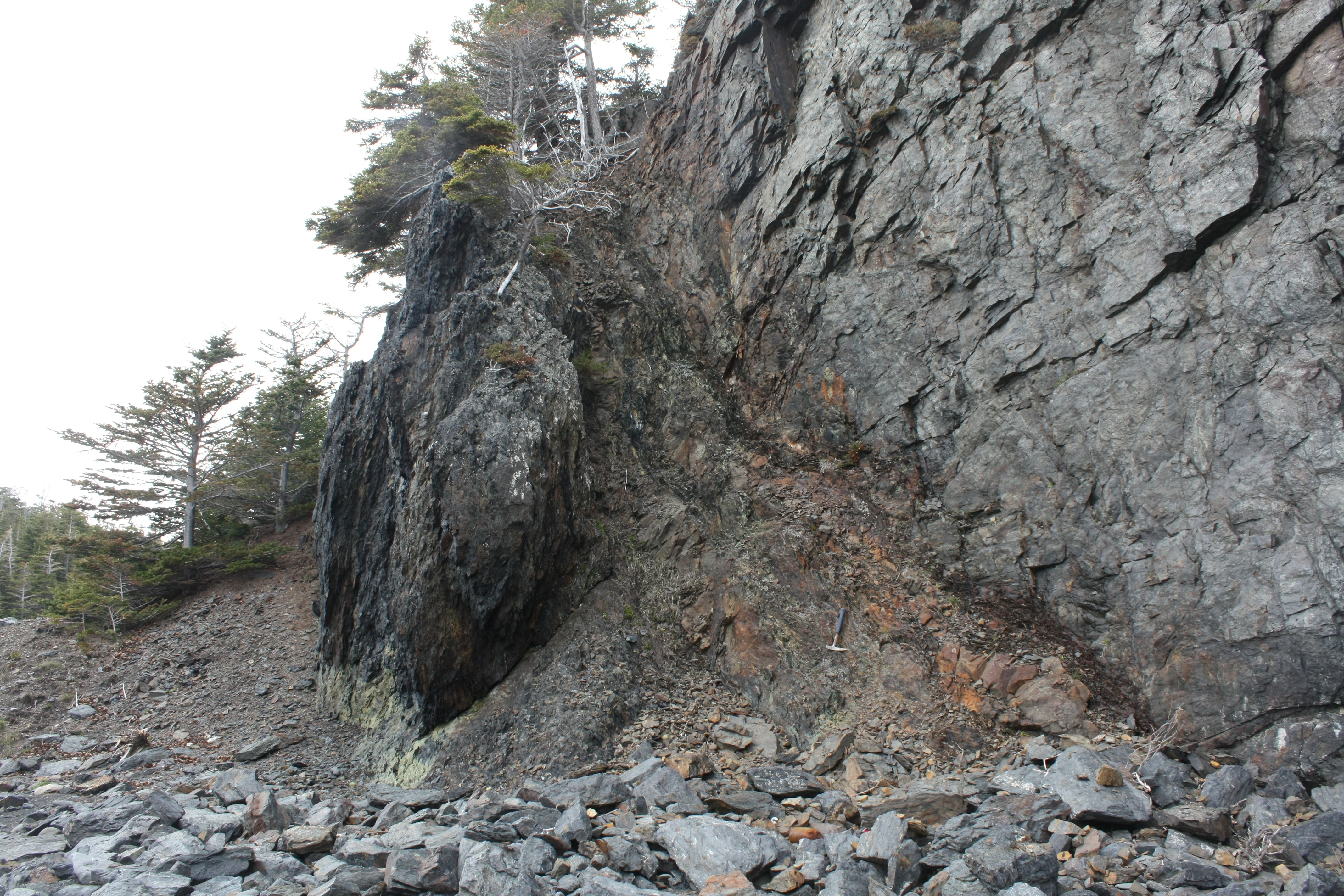

Supplement: Supplementary file 5 — Higher resolution version of field photographs (.jpg) contained in the Google Earth map file (.kmz). [file mmc6.zip › IMG_3833.JPG]

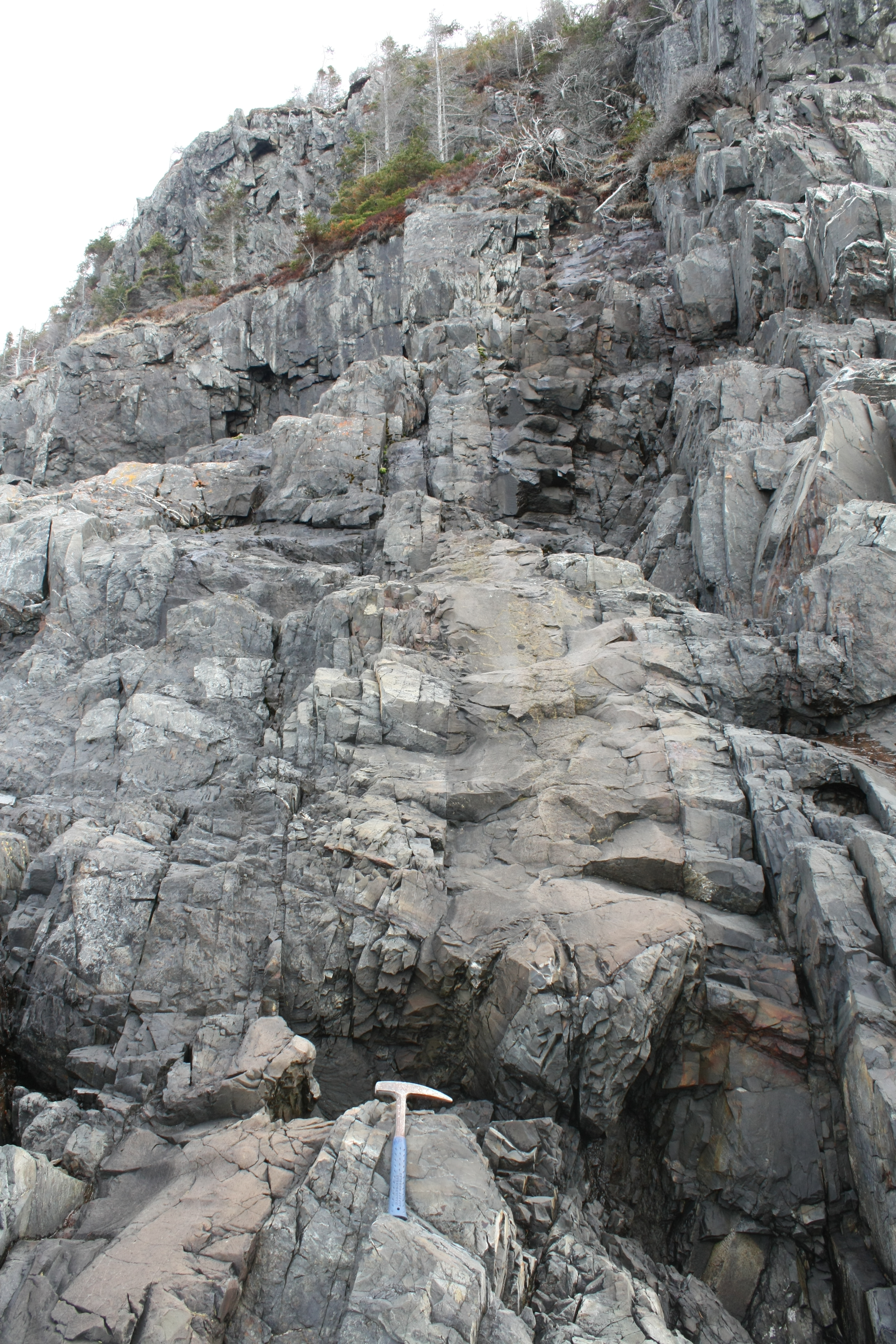

Supplement: Supplementary file 5 — Higher resolution version of field photographs (.jpg) contained in the Google Earth map file (.kmz). [file mmc6.zip › IMG_3844.JPG]

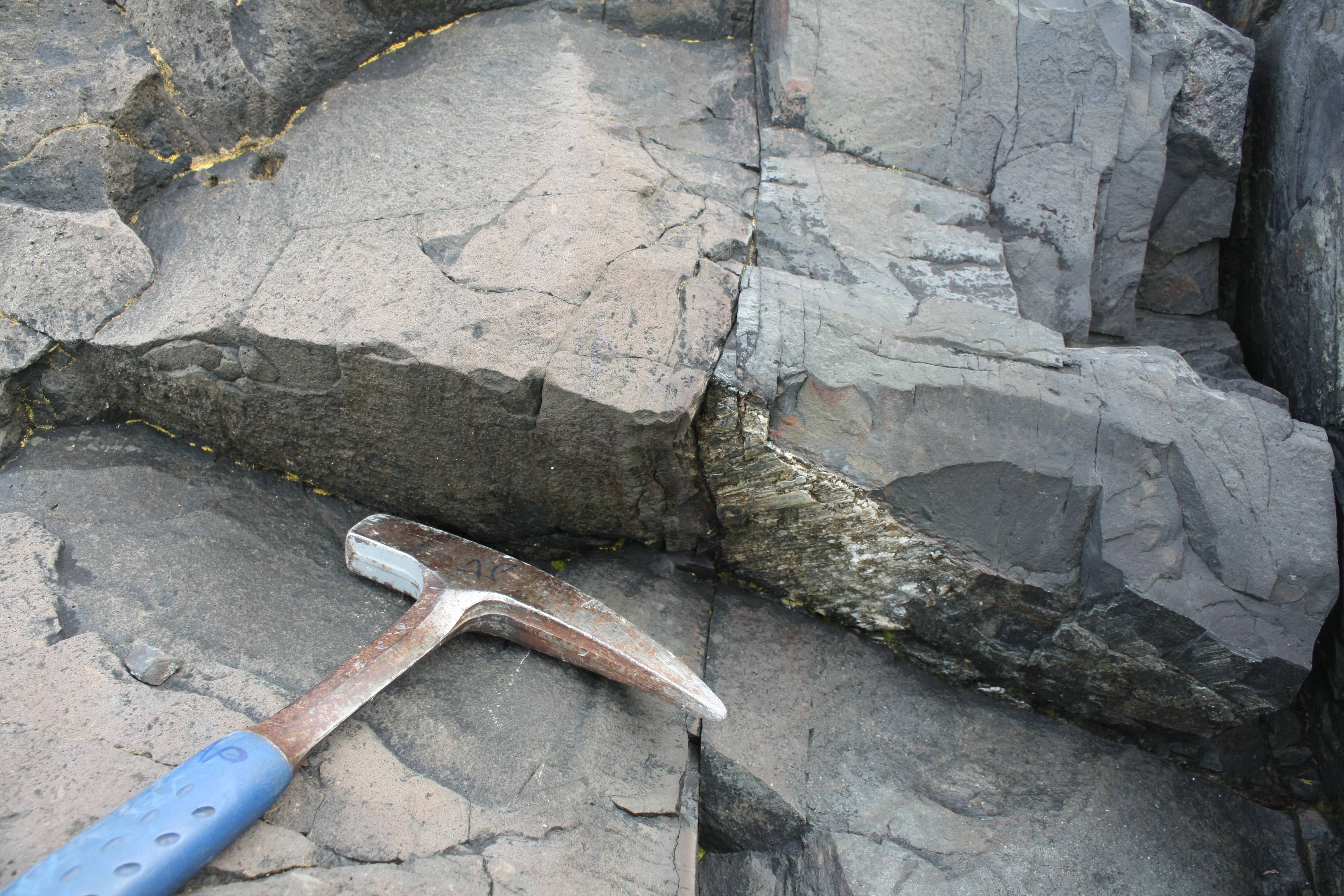

Supplement: Supplementary file 5 — Higher resolution version of field photographs (.jpg) contained in the Google Earth map file (.kmz). [file mmc6.zip › IMG_3849.JPG]
